# Efficacy of Rituximab in Refractory Inflammatory Myopathies Associated with Anti- Synthetase Auto-Antibodies: An Open-Label, Phase II Trial

**DOI:** 10.1371/journal.pone.0133702

**Published:** 2015-11-05

**Authors:** Yves Allenbach, Marguerite Guiguet, Aude Rigolet, Isabelle Marie, Eric Hachulla, Laurent Drouot, Fabienne Jouen, Serge Jacquot, Kuberaka Mariampillai, Lucile Musset, Philippe Grenier, Herve Devilliers, Adrian Hij, Olivier Boyer, Serge Herson, Olivier Benveniste

**Affiliations:** 1 Département de Médecine Interne et Immunologie Clinique, Centre de Référence des Pathologies Neuromusculaires Paris Est, UPMC, APHP, INSERM, UMR 974, DHU i2B, Hôpital Pitié-Salpêtrière, Paris, France; 2 Département de Biostatistique, UMRS 943, UPMC, INSERM, Paris, France; 3 Département de Médecine Interne, Hôpital Charles Nicole, Rouen, France; 4 Centre de Référence pour les maladies auto-immunes systémiques rares (Sclérodermie) Hôpital Claude Huriez, Université Lille 2, Lille, France; 5 Département d’Immunologie, U905, Université Rouen Normandie, INSERM, Hôpital Universitaire de Rouen, Rouen, France; 6 Laboratoire d'Immunochimie, Hôpital Pitié-Salpêtrière, UPMC, APHP, Paris, France; 7 Département de radiologie générale, Hôpital Pitié-Salpêtrière, UPMC, APHP, Paris, France; 8 Département de médecine Interne, Hôpital Universitaire Dijon, Dijon, France; 9 Département de Médecine Interne et Pathologie Vasculaire, Hôpital Saint Louis, Université Paris 7, APHP, Paris, France; Imperial College, London, UNITED KINGDOM

## Abstract

**Objective:**

Anti-synthetase syndrome (anti-SS) is frequently associated with myositis and interstitial lung disease (ILD). We evaluated prospectively, in a multicenter, open-label, phase II study, the efficacy of rituximab on muscle and lung outcomes.

**Methods:**

Patients were enrolled if they were refractory to conventional treatments (prednisone and at least 2 immunosuppressants). They received 1 g of rituximab at D0, D15, and M6. The primary endpoint was muscular improvement based on manual muscular testing (MMT10, Kendall score in 10 muscles) at M12. Secondary endpoints were normalization of creatine kinase (CK) level, ILD improvement based on forced vital capacity and/or diffuse capacity for carbon monoxide, and number and/or doses of associated immunosuppressants.

**Results:**

Twelve patients were enrolled, and 10 completed the study. Only 2 patients presented an improvement of at least 4 points on at least two muscle groups (primary end-point). Overall, seven patients had an increase of at least 4 points on MMT10. CK level decreased from 399 IU/L (range, 48–11,718) to 74.5 IU/L (range, 40–47,857). Corticosteroid doses decreased from 52.5 mg/d (range, 10–70) to 9 mg/d (range, 7–65) and six patients had a decrease in the burden of their associated immunosuppressants. At baseline, all 10 patients presented with ILD. At M12, improvement of ILD was observed in 5 out of the 10 patients, stabilization in 4, and worsening in 1.

**Conclusions:**

This pilot study of rituximab treatment in patients with refractory anti-SS provided data on evolution of muscular and pulmonary parameters. Rituximab should now be evaluated in a larger, controlled study for this homogenous group of patients.

**Trial Registration:**

Clinicaltrials.gov NCT00774462.

## Introduction

Anti-histidyl-tRNA synthetase (anti-Jo-1) auto-antibodies (aAbs) are found in approximately 25% to 30% of patients with idiopathic inflammatory myopathies [1]. Since anti-Jo-1 aAbs were first characterized, 7 other anti-aminoacyl-tRNA synthetase aAbs have been identified to date. Anti-Jo-1 aAbs occur most frequently, followed by anti-threonyl-tRNA synthetase (anti-PL-7) and anti-alanyl-tRNA synthetase (anti-PL-12) aAbs. In addition to being associated with myositis, anti-aminoacly-tRNA synthetase aAbs are usually associated with interstitial lung disease (ILD), which is the major determinant of morbidity and mortality [2]. Arthritis, Raynaud phenomenon, and mechanic’s hands are also frequently observed in patients with anti-synthetase syndrome (anti-SS) [3]. Along their follow-up, more than two-thirds of patients with anti-SS need increased prednisone doses, adjunct therapy, and/or a change to a different immunosuppressive or modulator drugs, *i*.*e*., disease-modifying anti-rheumatic drugs, because initial treatment was ineffective [2]. No particular combination can be recommended because none appears to prevent treatment intensification [2].

Since Jo-1 antigen and anti-Jo-1 aAbs may have a role in the pathophysiology of anti-SS [4–7], the use of B-cell targeted treatment may be of interest, as it was previously shown to be effective in anti-neutrophil cytoplasmic aAbs–associated vasculitis [7] and rheumatoid arthritis [8].

At the initiation of this study, only case reports [9,10] and retrospective case series [11] indicate that B-cell depletion using monoclonal anti-CD20 antibody rituximab may be beneficial. Since then, a prospective randomized clinical trial of rituximab for the treatment of refractory myositis from different origins (juvenile and adult dermatomyositis, polymyositis, anti-signal recognition particle necrotizing myopathies, and anti-SS) was published [12], but no clinical trial, except this one, has addressed the efficacy of rituximab in a prospective fashion in a homogenous group of patients with anti-SS refractory to conventional therapies.

## Patients and Methods

### Patients

Patients with anti-SS were eligible to participate in this pilot, open-label, prospective, multicenter (this study was conducted in 4 French adult internal medicine departments), phase II study if they were considered refractory to conventional treatments. Patients were defined as having anti-SS if they had myositis based on the 119th European Neuro Muscular Centre criteria [13], including a proximal myopathy with weakness, a subacute or insidious onset over 18 years, myogenic syndrome diagnosed via electromyogram, and muscle fibre necrosis and regeneration and/or inflammatory cell infiltrate diagnosed via muscle biopsy, associated with the presence of anti-Jo-1 or anti-PL-7 or anti-PL-12 aAbs. Anti-Jo-1, anti-PL-7, anti-PL-12 aAbs were detected using an immunodot assay (Euroline Myositis Profile 3, Euroimmun, Lübeck, Germany). Refractory anti-SS was defined as intolerance or inadequate response to glucocorticoids and at least 2 other immunosuppressive or immunomodulatory agents, *e*.*g*., azathioprine, methotrexate, mycophenolate mofetil, cyclosporine, tacrolimus, cyclophosphamide, or intravenous immunoglobulin (IVIg). These treatments had to have been unsuccessful for more than 6 months before study start. Inadequate response was defined by the absence and/or worsening of one of the following parameters: muscle strength assessed by MMT10 score using Kendall score, creatine kinase (CK) level (> 3 fold the upper limit of the normal range, controlled twice), and/or pulmonary function tests (PFT).

Exclusion criteria were myositis associated with cancer or with connective tissue disease, pregnancy, severe cardiac dysfunction (ejection fraction ≤ 30%), severe respiratory dysfunction (forced vital capacity < 1000 mL and/or ≤ 30% of the predicted value), severe adverse reaction after monoclonal aAbs infusion, active infectious disease including bacterial or viral infections (such as human immunodeficiency virus or hepatitis B virus), anaemia (haemoglobin level < 8g/dL), neutropenia (absolute neutrophil count < 1500 × 10^3^/μL), and immunoglobulin G and/or M < 5.0 or 0.40 mg /mL, respectively.

The patients were screened during a clinic visit or hospitalization and recruited after verification of eligibility criteria. The inclusion period was 3 years and started in January 2008. The last patient was enrolled in April 2010. The patients participated for a period of 18 months.

### Ethics statement

Written informed consent was obtained from each study patient. The protocol was approved by the Pitié-Salpêtrière Hospital ethics committee (June 2007) and the French Health Products Safety Agency (Afssaps) (ref. Afssaps: A70399-45; EudraCT: 2006-005900-15; clinicalTrials.gov no. NCT00774462). The authors confirm that all ongoing and related trials for this drug/intervention are registered.

### Treatments

Patients received two 1 g infusions of rituximab separated by 2 weeks, followed by 1g infusion 6 months after the day 15 injection was performed. Depending on the their history of immunosuppressant resistance, patients were allowed to receive prednisone with dose enhancement up to 1 mg/kg/day, sometimes preceded by a bolus of methylprednisolone maintenance or change of other immunosuppressive drugs, plasma exchange before rituximab infusion, and/or IVIg injection (Table 1).

**Table 1:** Patients characteristics, muscular evaluation, and treatment administration at baseline and month 12.

#	Sex	Age	Disease	Traitements	Treatments	Kendall score/100		CK (I.U./L)	
		(yrs)	duration (yrs)	before enrollement	at inclusion	M0	M12	M0	M12
1	M	20	2	CT, MTX, AZA, MMF, RTX	CT, AZA	94	100	11718	58
2	M	56	1	CT, MTX, AZA, IVIg, MMF	CT, MMF	87	100	53	72
3	M	54	0.5	CT, AZA, CYC, IVIg	CT	75	86	605	148
4	M	30	1	CT, AZA, IVIg	CT, MTX, IVIg	94	100	3704	43
5	W	58	4	CT, IVIg, MMF	CT, MMF	91	77	822	470
6	W	46	3	CT, IVIg, MMF	CT	99	84	75	47857
7	M	42	4	CT, MTX, AZA, IVIg	CT, AZA	86	91	48	53
8	M	64	8	CT, MTX, AZA, IVIg	CT, AZA	82	90	193	40
9	M	58	8	CT, AZA, IVIg	CT, AZA, IVIg	99	100	141	77
10	M	59	2	CT, MTX, AZA, IVIg	CT	95	100	2840	99

M: man

W: woman

CT: corticosteroid

MTX: methotrexate

AZA: azathioprine

MMF: mycophenolate mofetil

IVIg: intravenous immunoglobulins

CYC: cyclophophamide.

### Assessments

Study visits occurred at baseline, D7, D15, D21, M6, M12, and M18 after the first rituximab injection. Muscle weakness was determined on the basis of manual muscular testing using the Kendall test (score ranged from 0 to 10; 0 = paralysis, 10 = normal strength) on 10 muscle groups, including axial (neck flexor), proximal (deltoid, brachial triceps and biceps, psoas, gluteus medius and maximus, and quadriceps) and distal muscles (wrist extensor and ankle dorsiflexor) on the dominant side (maximum score: 100) [14]. Serum CK levels were measured on the same dates. Health-related quality of life was scored with the use of the Rand Medical Outcomes Study 36-Item Short-Form Health Survey (SF-36) [15–17].

Lung involvement was assessed by using PFT at M0, M6, and M12. Computed tomography (CT) of the chest with multidetector (16–64) was performed at M0 and M12. Anti-Jo-1 antibody levels were determined using ALBIA (Addressable Laser Bead Immuno Assay) technology (Fidis-Connective Profile, Theradiag-BMD, Marne La Vallée, France).

Immunophenotyping of peripheral blood mononuclear cells was performed by flow cytometry. For peripheral blood B lymphocyte count, blood cells were stained with anti-CD19 (B4, mouse IgG1) fluorescein isothiocyanatelabelled monoclonal aAbs (Beckman Coulter, Miami, Florida, USA) with the Coulter PrepPlus and TQPrep cell preparation systems and were analyzed on an Epics XL flow cytometer (Beckman Coulter) with the System II and Expo 32 software. Absolute lymphocyte counts were determined using Flow-Count Fluorospheres (Beckman Coulter).

When B cells were detected, additional characterization of B cell subsets (CD38^high^ CD24^high^ transitional B cells, CD27^+^ IgD^-^ switched memory B cells and CD27^+^ IgD^+^ marginal zone-like B cells) was performed with CD19 (J4119, mouse IgG1) ECD-labelled, CD24 (ALB9, mouse IgG1)-PE, CD38 (LS198-4-3, mouse IgG1)-PC5, CD27 (1A4, mouse IgG1)-PC5 monoclonal antibodies from Beckman Coulter and anti-IgD rabbit serum-PE from Dako (Glostrup, Denmark).

### End points

The primary end point, was defined as an improvement in muscle strength characterized by an increase of at least 2 Kendall points in at least 2 different muscle groups at M12.

Secondary end points, measured at M12, were: an improvement (increase of 4 points on Kendall score) or worsening (decrease of 4 points on Kendall score) of muscle strength in at least one muscle group, a decrease in CK levels, a decrease in the titre of anti-Jo-1 aAbs, an improvement in ILD, an improvement of SF-36 score (+10 and +5 points for multi-item scales and the physical and mental components summaries, respectively [17]), and a decreased need for immunosuppressive treatment. A decreased need for immunosuppressive treatment was defined as the discontinuation of at least 1 immunosuppressant/immunodulatory drug and/or a ≥ 50% decrease in prednisone dose at M12 compared with M0. On the other hand, the addition of an immunosuppressant/immunodulatory drug was considered as treatment intensification, and no change in treatment was considered as treatment stabilization. These 2 categories defined patients who had no decrease in treatment.

An improvement in ILD was defined as an increase of 10% in FVC (with or without a concomitant change in diffusing capacity for carbon monoxide adjusted on haemoglobin level, DlCOcor) or an increase of 15% in DlCOcor (with or without a concomitant change in FVC) at M12 compared with M0 [18]. ILD worsening was defined as a decrease of 10% in FVC and/or 15% of DlCOcor values at M12 compared with M0 [18].

### Adverse events

The data safety and monitoring board monitored overall safety independent of the participating institutions.

### Statistical analysis

In this pilot study, no calculation of sample size was made. Based on the initial provision of inclusion of 12 patients, observation of an improvement in muscle strength among six patients would have resulted in a success rate estimate above 25% (inferior limit of 90% confidence interval).

Descriptive statistics were displayed using median and range, or n and percentage with 95% confidence interval. Variations of continuous variables from baseline to M12 or M18 were studied using a Wilcoxon signed-rank test of match pairs under the null hypothesis that the median of the differences is zero.

The trial study protocol is available in S1 Appendix (in the original language) and S2 Appendix (in English).

Data underlying the findings in the FORCE study are freely available in the S1 Table. The Consolidated Standards of Reporting Trials checklist is available in the S3 Appendix.

## Results

### Patient characteristics at baseline

**Fig 1:**
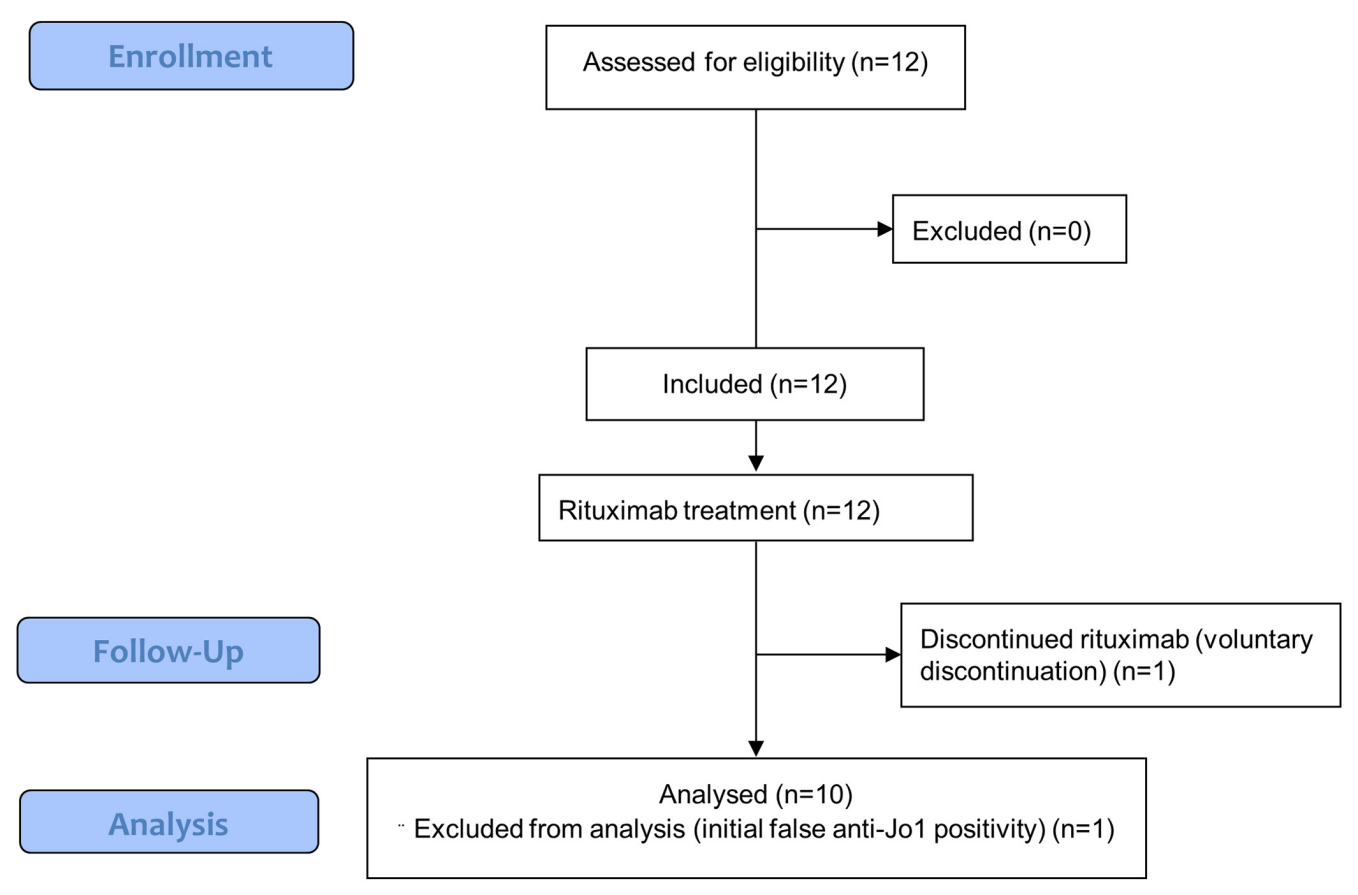
Flowchart.

Patient characteristics are presented in Table 1. Twelve patients were enrolled in the study (in 27 months), but 1 patient withdrew at week 3 (after the second rituximab infusion) because he decided to be followed-up elsewhere); a second patient was excluded because the presence of anti-synthetase aAbs was not confirmed during the study (initial false anti-Jo-1 positivity) (Fig 1). Ten patients completed the study: 8 men and 2 women. Nine patients were anti-Jo-1 aAbs positive and 1 patient was anti-PL-7 positive. The median age was 51 years (range, 18–57 years) at diagnosis of anti-SS and 55 years (20–64 years old) at the date of entry into the study. Before enrolment, patients previously received a median of 3 immunosuppressive drugs and/or immunomodulatory treatments (Table 1) in addition to corticosteroids. The median MMT10 score was 92.5 (range, 75–97) and the median CK level was 399 IU/L (range, 48–11,718), and 4 of the 10 patients had normal CK levels. All patients had ILD confirmed by CT scan showing a nonspecific interstitial pneumonia (n = 9) and/or organized pneumonia (n = 1). Before enrolment, lung function deteriorated in 5 patients with ILD, as measured by PFT, realized 6 months prior to enrolment, 3 had no significant change, and 2 had no PFT data available at this date. At baseline, median CV, and DLCO, were 72% (range, 46–117) and 45% (range, 22–65). At baseline, SF-36 scores were low, with a median physical functioning norm based score of 38.1 (range, 31.7–54.9), with a median physical component summary score of 37.7 (range, 15.2–54.3) and a median mental component summary score of 36.2 (range, 10–54.8), knowing that 50 is considered as normal.

### Strength change

**Fig 2:**
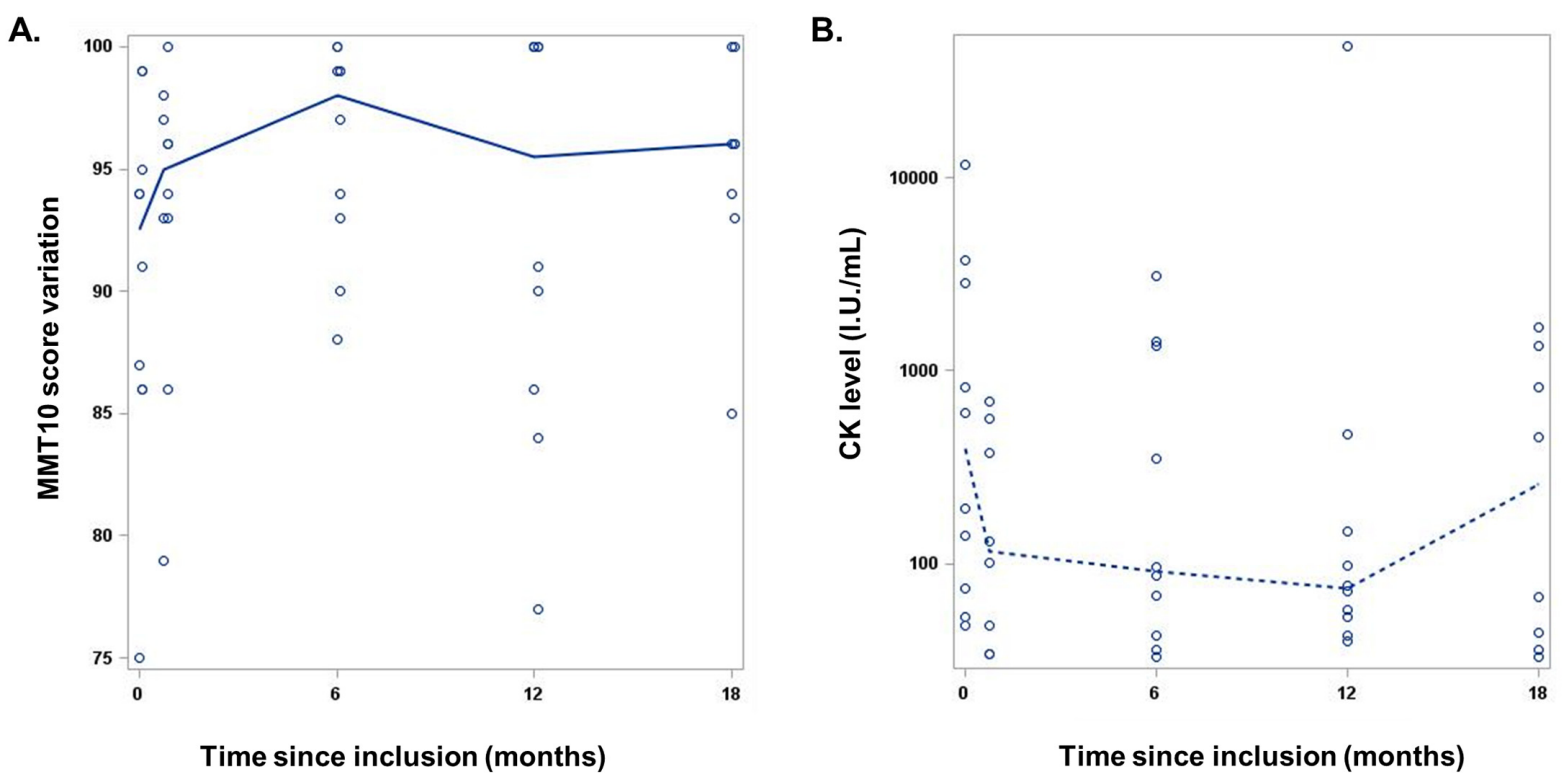
Evolution of strength and CK levels from baseline to months 18. (A) MMT10 using Kendall score. (B) CK level. The continuous line and the dotted line represent the median Kendall score and the median CK level, respectively.

At M12, only 2 patients achieved the primary end point, which required improvement in 2 different muscle groups. The median MMT10 score increased from 92.5 (range, 75–97) at baseline to 95.5 (range, 77–100) at M12 with a median variation of +5 points (range, -15 to +13; p = 0.43). Overall 7 of 10 patients had an increase of at least 4 points (70%, 95%CI, 35–93%). One patient was stabilized and 2 worsened (Table 1 and Fig 2).

At M12, median CK level decreased to 74.5 IU/L (range, 40–47,857) compared with the baseline value of 399 UI/L (range, 48–11,718), with a median variation of -252.5 UI/L (range, -11660 to +47782; p = 0.16) (Table 1 and Fig 2). All patients with improving MMT10 score had a normal CK level at M12 (Table 1).

At M18, over the two patients who presented with an improvement on two muscle groups at M12, only one maintained this improvement with an additional improvement on a third muscle group. At M18, the median MMT10 was 96 (n = 8; range, 85–100) and the median variation was + 4 (range, -3 to +10). The median CK level was 260 IU/L (range, 33–1683), and 2 patients had increased levels. Overall, the median variation was -56 IU/L (range, -11266 to +745).

### Treatment modification

At M12 treatment was decreased for 6 patients (Table 1). The median corticosteroid dose was 52.5 mg/d (range, 10–70) at month 0, 9 mg/d (range, 7–65) at M12. Two patients had treatment intensification (1 had 6 cyclophosphamide pulses followed by tracrolimus, and 1 had methotrexate introduction after M6). Two patients had stabilized treatment (1 with corticosteroids combined with azathioprine, 1 with corticosteroids alone).

At M18, treatment was decreased for 4 patients compared to baseline and the median dose of corticosteroid was 7 mg/d (range, 4–35).

### Extramuscular involvement change

**Fig 3:**
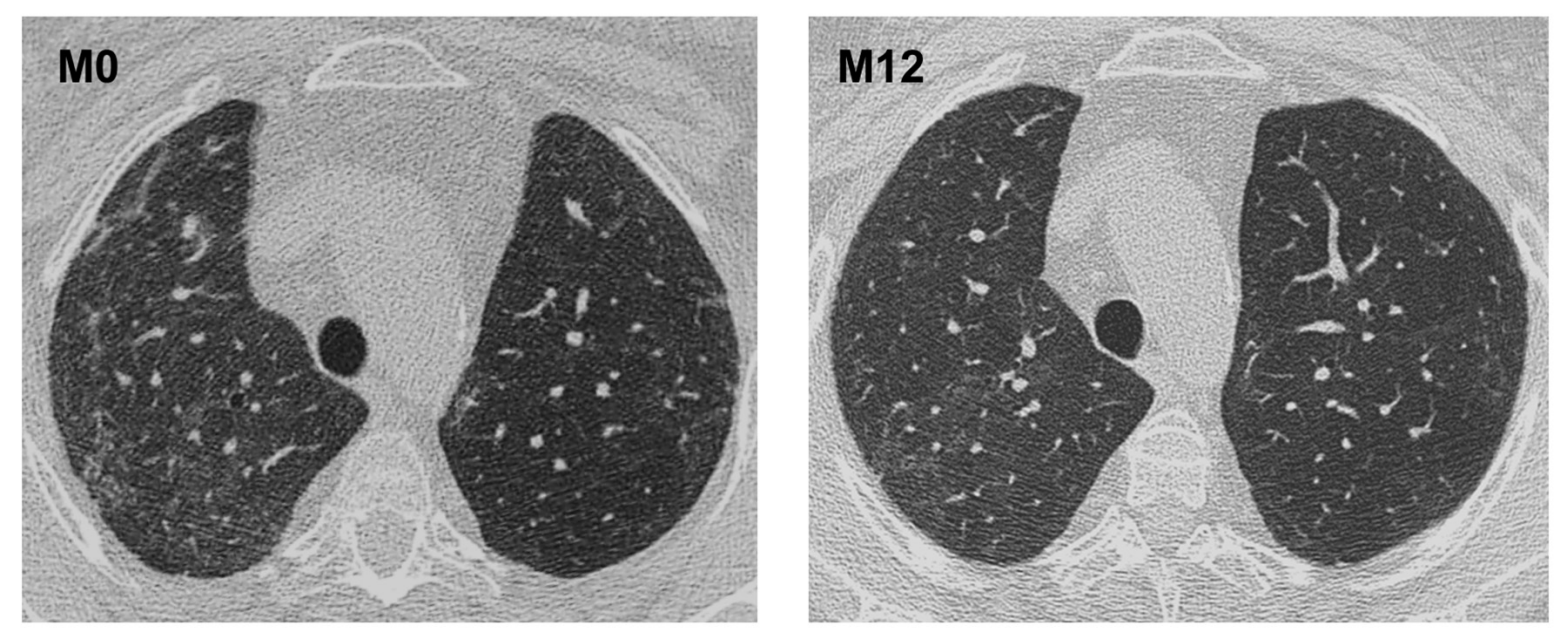
Interstitial lung disease evolution. Lung CT scans, before enrolment (M0) and 1 year later (M12), of the only patient with and nonspecific interstitial pneumonia improving after rituximab infusions.

At M12, effort dyspnea continued in 4 out of the 10 patients (vs. 6 at baseline). Pulmonary CT scan at M12 compared with M0 showed a clear reduction of interstitial infiltrates in only 1 patient (Fig 3) and an aggravation in 1 other patient, whereas no significant change was observed for the remaining patients.

**Fig 4:**
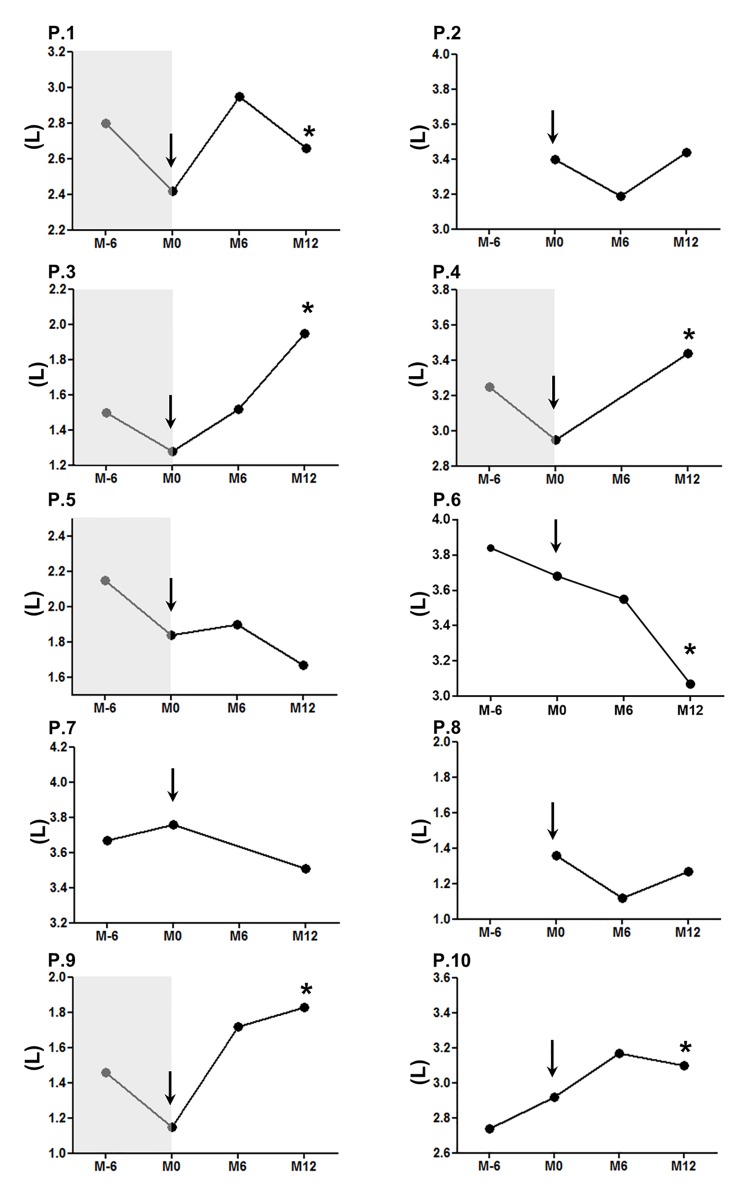
Forced vital capacity evolution. Forced vital capacity (FVC) is represented for each patient who terminated the study and had interstitial lung disease (ILD). FVC is represented 6 months before enrolment (M−6), when available, and 6 and 12 months (M6 and M12) after the first rituximab infusion (arrow, M0). The grey area represents a decrease of ≥ 10% in absolute FVC at baseline compared with M−6. (*) Represents an increase or a decrease of ≥ 10% of absolute FVC or ≥ 15% of DLCOcor at M12 compared with M0.

At M12, median variation of FVC was 5% (range, -16% to +59%; p = 0.23); improvement of FVC was observed in 4 patients, stabilization in 5, and worsening in 1 (Fig 4). Only 1 patient with increased FVC showed also an improvement of DLCOcor. In addition, 1 patient had an improvement of DLCOcor without significant change for FVC (data not shown). Finally 5 patients had improving ILD measured by PFT (50%; 95% CI, 19–81).

**Fig 5:**
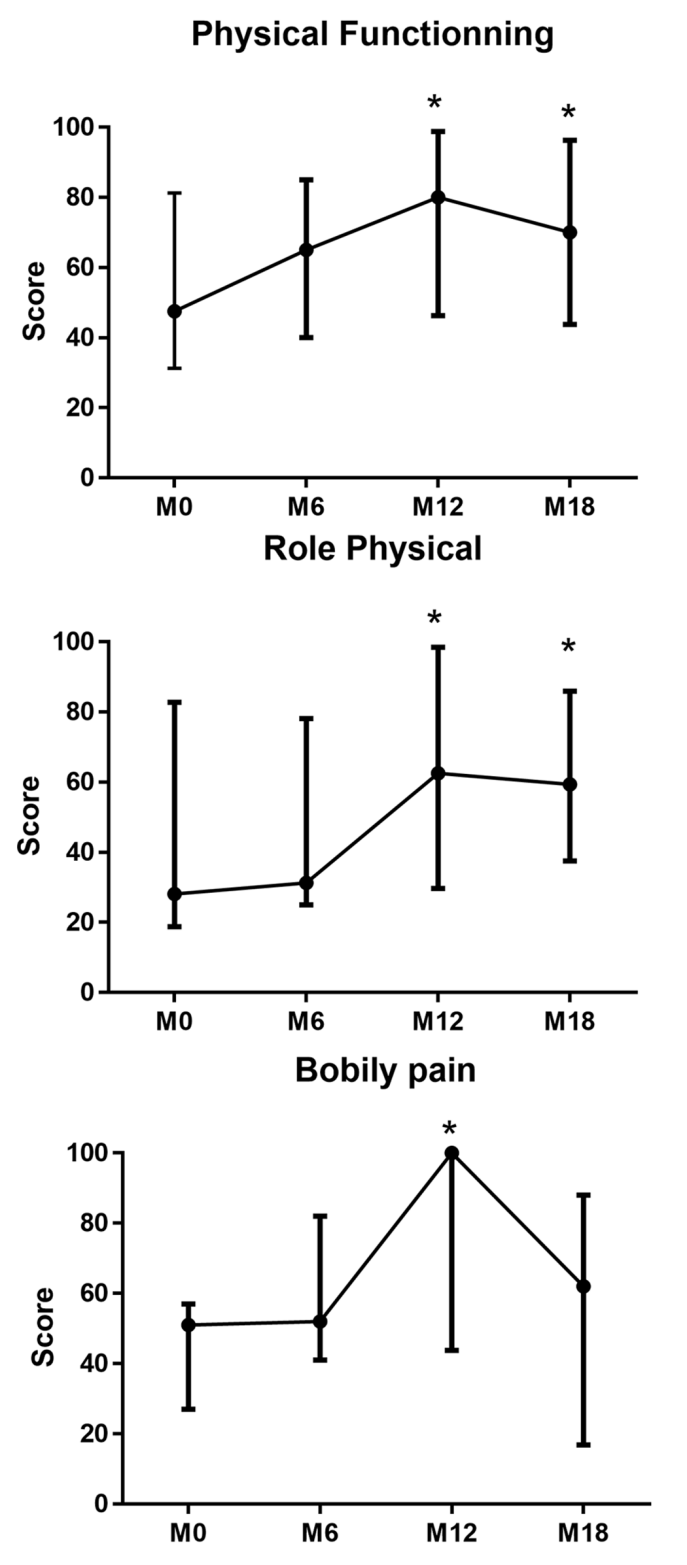
SF-36 scores measuring parameters of mobility. For each score (ranging from 0 to 100) the mean values (± SD) are represented at different time points: baseline (M0); months 6, 12 and 18 after rituximab infusion (M6, M12 and M18). The asterisk represents a significant increase (more than 10 points) compared with baseline.

At M12, median values for the SF36 components were 48.6 (range, 29.5–57) and 47.7 (range, 41.3–59.5) for the physical functioning norm based score and the mental component summary score, respectively, whereas the physical component summary remained stable (median 37.0; range, 27.6–58.4). In addition to physical functioning, SF-36 scores measuring mobility parameters such as role physical and bodily pain increased with median variations between M0 and M12 of +25, +19, and +49, respectively (Fig 5).

### Safety analysis

The patient who withdrew at week 3 (after the second rituximab infusion) did not experience any particular side effect, and we continued to receive news from him attesting that he is in a good condition. One other patient experienced acute respiratory distress syndrome justifying transient assisted invasive mechanical ventilation 8 weeks after the first rituximab infusion and 6 weeks after administration of methotrexate therapy weekly. This severe adverse event was related to methotrexate (which was withdrawn), because no pathogen was identified, and patient improved after methotrexate withdrawn. Nevertheless, since a role for rituximab cannot be formally ruled out, the third injection (at M6) was not performed, but the patient was still followed up, as scheduled in the trial protocol. Furthermore, 6 possibly infectious adverse events occurred during the study period and remained mild to moderate: 1 febrile episode, 2 urinary tract infections, 1 herpes zoster infection, 1 non severe pneumonia (without documented bacteria), and 1 vulvovaginal candidosis. Of note, no severe adverse event occurred during rituximab infusion and no malignancy was detected during the study.

### Immunological analysis

**Fig 6:**
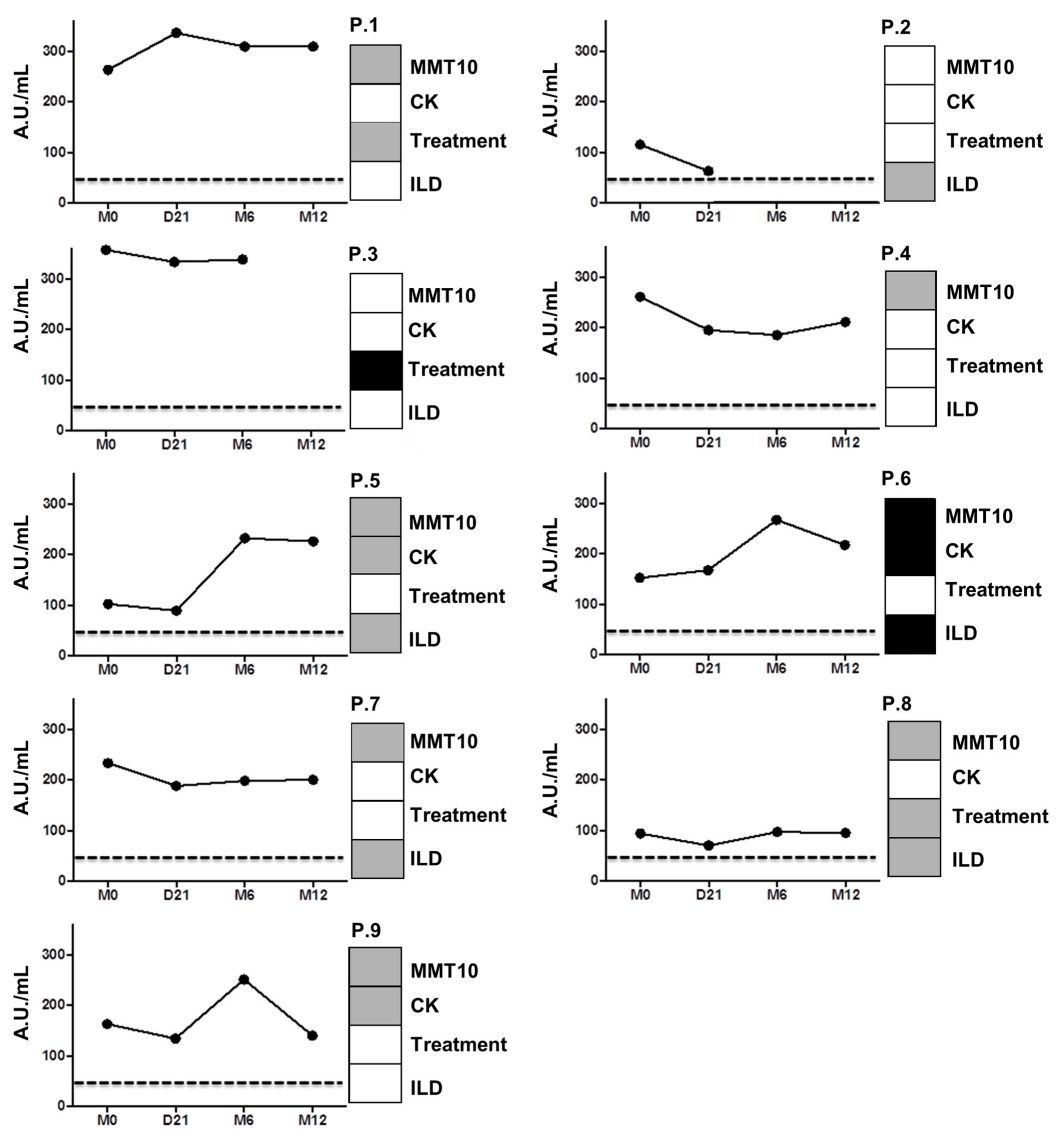
Anti-Jo-1 titer variation and B-lymphocyte depletion. Anti-Jo-1 titer was monitored for the 9 patients who terminated the study (the tenth had anti-PL-7). On the right side, 4 boxes represent the status at M12 (vs M0) concerning manual muscular testing (MM10), creatine kinase level (CK), treatment modification (treatment), and forced vital capacity (FVC). Black represents worsening, grey area represent no change, and white represents an improvement. Concerning CK, black shows an increased level at M12 and white a normal level.

For the nine anti-Jo-1 aAbs–positive patients, it was possible to follow the titer of aAbs (Fig 6). No significant variations among the anti-Jo-1 titers were observed over time (median, 163 AU/mL; range, 94–357) at M0 vs. 211 AU/mL (range, 95–309) at M12 (median variation +1 AU/mL; range, -50 to +124; p = 0.58) (Fig 6). Nevertheless, the 2 patients (patient 5 and 6) who presented at M12 with an important increase of their titer (increases of 42% and 121%, respectively; Fig 6) were those with both MMT10 scores and CK levels lower than at baseline.

Flow cytometry analysis performed at D21 evidenced complete CD19^+^ B-cell depletion (no detectable CD19^+^ cells) for all patients except 1 (0.1% of peripheral mononuclear cells, 1.7 cells/mm^3^). Along the same lines, treatment intensification did not correlate with emergence of a B-cell subpopulation, such as plasmablasts, transitional B cells, or memory-type B cells (not shown). Finally, no significant change was observed in total lymphocyte, red cell, or platelet counts at M12 compared with baseline (data not shown).

## Discussion

This study is a prospective trial that has for the first time exclusively enrolled patients with anti-SS to be treated by rituximab. These patients were considered as refractory because of the number of immunosuppressants they received prior to study entry. At M12, only 2 patients achieved the primary end point of an improvement on two muscle groups even if 7 had improved muscle parameters (MMT10 and CK levels) as well as extramuscular involvements.

The ILD (measured by PFT) improved for 5 of 10 patients and was stabilized (n = 4) or worsened (n = 1) for the 5 remaining patients. Furthermore, 6 of 10 patients were able to decrease treatment, which was associated with an improved quality of life. This beneficial effect seems transient since at M18, 4 had relapsed (based on MM10 score).

To our knowledge, there are only 3 prospective clinical trials using rituximab to treat anti-SS patients with n = 2 [19], n = 7 [20] and n = 32 [12], but inclusion criteria and outcomes differ from one to another, making comparison with our study difficult.

Here, we included patients who had relapsed and presented with ILD. ILD has been shown to be strong predictor for use of DMARD [2] and poor survival [21]. Thus, it was not ethically possible to design a placebo group (control group) considering the long duration of study (18 months), neither a standardized association of immunosuppressants to compare the patients. There is indeed no standard therapeutic recommendation for such patients, and treatments have to be adapted to the past history of these patients. Only the Rituximab in Myositis (RIM) trial had a control placebo group for only 2 months; it was the delay of rituximab administration that was randomized [12]. During this ‘randomized placebo phase design’, the placebo control phase lasted only 8 weeks, leading to the statistical failure of the trial since the mean time to achieve the primary outcome was much longer than 8 weeks (20.2 weeks) [12].

Despite its limited population size, one strength of the present study is that we enrolled prospectively a homogenous group of patients who had well-defined anti-SS syndrome based on a serologic test. Other prospective studies were based on inhomogeneous groups [12,19,20], i.e., including patients with dermatomyositis (sometimes juvenile patients), polymyositis with or without anti-SS, and necrotizing autoimmune myopathies associated with anti-signal recognition particles aAbs. This great heterogeneity of patients makes the choice of outcomes difficult as the interpretation of the results.

End points for clinical trials in myositis are difficult to define because myositis encompasses very heterogeneous groups of patients with variable muscular and extramuscular involvement and because strength-assessing tools are difficult to establish. In 2004, the International Myositis Assessment and Clinical Studies Group (IMACS) [22] recommended tools that we did not use since when the study was designed, the assessments tools it proposed had not been fully validated, and because a visual analogue scale (0–10) was proposed for muscular assessment. Along that line, later in 2010, it was shown that MMT focusing on a subset of eight muscles (Kendall grading scale) was a good outcome measure for myositis (MMT8) [23]. In 2014, an improvement of 20% of the MMT8 score, was an item of a core set measure used as out-come in the RIM trial [12]. Here we used MMT10 with Kendall grading scale. However, the validity of MMT grading scales was recently discussed [24]. The results of our study suggested that monitoring CK level could be more sensitive for muscular assessment.

For chest evaluation, we used PFT, a reproducible and objective tool recommended for ILD assessment [18], and not the core set of measures (including subjective tools with visual analog scales) recommended by IMACS and used in the RIM trial. This is the first time that PFT was used as an end point in a prospective trial that included exclusively anti-SS patients, and the improvements we observed are in line with those observed in a retrospective study [11]. Of note, only 2 patients have an improvement of DLCOcor measurement whereas 5 patients had an in increase of volume. This suggests that PFT improvement may be mainly due to muscular strength reinforcement rather than improvement of ILD. Along that line, only one patient showed a reduction of interstitial infiltrates on CT scan. The presence of anti-Jo-1 aAbs was one of the strongest predictor of clinical improvement in the RIM trial (including adult and paediatric myositis patients) [25]. In addition, a correlation between anti-Jo-1 aAbs titers and parameters of clinical disease activity had been reported [26]. Here, all but one patient were anti-Jo-1 aAbs positive. We did not observe clear pattern of variation of anti-Jo-1 aAbs titers associated with out-comes measures, but the two patients with muscular worsening (MMT10 and CK) had increased titers.

After rituximab infusion, the level of B-cell depletion has been correlated with the risk of relapse in rheumatoid arthritis [27] as in anti-neutrophil cytoplasmic aAbs–associated vasculitis [28], although this has not been confirmed in all studies [29]. The results we obtained concerning B-cell depletion do not allow such a conclusion.

Finally, adverse event related to the rituximab were mainly infections. None of the infections required hospitalization. The acute respiratory insufficiency was probably due to methotrexate toxicity. Infectious was also the most frequent adverse event related to the drug in the RIM study [12]. Since patients we enrolled had concomitant immunosuppressant and/or corticosteroids, the role of rituximab in occurrence of infections cannot be evaluated without control group.

To conclude, this pilot, prospective, phase II study provide data on the effect of rituximab on both muscular and extramuscular manifestations in patients with refractory anti-SS. It supports the development of a prospective, controlled clinical trial including exclusively anti-SS patients to test the efficacy of rituximab and to identify predictors of efficacy.

This work was supported by the French Ministry of Health (PHRC) and the Association Française contre les Myopathies (AFM). Rituximab was kindly provided by Roche.

## Supporting Information

S1 Appendix Trial study protocol (FORCE) in the original language (French). (DOCX)

S2 Appendix Trial study protocol (FORCE) in English. (DOC)

S3 Appendix Consolidated Standards of Reporting Trials checklist. (PDF)

S1 Table Data underlying the findings in the FORCE study. (XLSX)
