# Supplementary material for: Efficacy of Rituximab in Refractory Inflammatory Myopathies Associated with Anti- Synthetase Auto-Antibodies: An Open-Label, Phase II Trial
Source: PLoS One. 2015 Nov 5;10(11):e0133702. doi: 10.1371/journal.pone.0133702 (PMC4634756; doi:10.1371/journal.pone.0133702)
Supplement: S1 Appendix — (DOCX) [file pone.0133702.s001.docx]

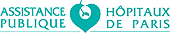


**Rituximab (anticorps monoclonal anti-CD20 dépléteur des lymphocytes B) pour le traitement des myopathies inflammatoires réfractaires avec auto-anticorps spécifiques et des myasthénies généralisées réfractaires.**

**FORCE**

[1- SYNOPSIS 8](#_Toc403643386)

[Schéma de l’étude 9](#_Toc403643387)

[2- JUSTIFICATION SCIENTIFIQUE ET DESCRIPTION GENERALE DE LA RECHERCHE: 10](#_Toc403643388)

[2.1 Généralités 10](#_Toc403643389)

[2.2 Situation de l’équipe de l’investigateur coordonnateur et des autres centres 11](#_Toc403643390)

[2.3 Description des pathologies 11](#_Toc403643391)

[Myopathies inflammatoires 11](#_Toc403643392)

[Myopathies inflammatoires réfractaires associées à des auto-anticorps spécifiques. 11](#_Toc403643393)

[Myopathies inflammatoires primitives : 12](#_Toc403643394)

[Myopathies inflammatoires secondaires : 13](#_Toc403643395)

[Résultats acquis : 13](#_Toc403643396)

[Myasthénie généralisée 14](#_Toc403643397)

[Résultats acquis : 15](#_Toc403643398)

[3 OBJECTIFS DE LA RECHERCHE 15](#_Toc403643399)

[3.1 Hypothèses cliniques 15](#_Toc403643400)

[3.2. Objectifs 15](#_Toc403643401)

[3.3. Bénéfices / risques 16](#_Toc403643402)

[4 CONCEPTION DE LA RECHERCHE 17](#_Toc403643403)

[4.1. Critères d’évaluation 17](#_Toc403643404)

[4.2. Description de la méthodologie 18](#_Toc403643405)

[4.3. Durée de participation et description de la chronologie du suivi des patients 18](#_Toc403643406)

[La durée prévisible des inclusions sera de 2 3 ans. La durée de participation des patients sera de 18 mois et la durée totale de l’étude de 4 5 ans. Les patients ne pourront pas participer à une autre recherche biomédicale pendant les 18 mois de participation à l’étude. 18](#_Toc403643407)

[Visite de pré-inclusion (S-4 à S-1) –Hospitalisation de jour 18](#_Toc403643408)

[Période d’inclusion : Jour – 1(J-1) au jour + 2 (J2). 19](#_Toc403643409)

[Myosites et myasthénies 19](#_Toc403643410)

[Période de suivi : Jours 7, 14, 21 et mois 6,5 (+/-8 jours), 12 et 18 : 20](#_Toc403643411)

[Schéma de l’étude : 21](#_Toc403643412)

[4.4. Règles d’arrêt définitif ou temporaire 21](#_Toc403643413)

[5 Sélection et exclusion des personnes de la recherche 22](#_Toc403643414)

[Myosites 22](#_Toc403643415)

[Critères d’inclusion : 22](#_Toc403643416)

[- hommes ou femmes de 18 ans à 70 ans ; 22](#_Toc403643417)

[- myopathies inflammatoires idiopathiques définie par : 22](#_Toc403643418)

[- Réfractaires aux traitements conventionnels 22](#_Toc403643419)

[Critères de non-inclusion : 22](#_Toc403643420)

[Critères d’inclusion : 24](#_Toc403643421)

[- hommes ou femmes de plus de 18 ans à 70 ans 24](#_Toc403643422)

[- Myasthénie généralisée définie par : 24](#_Toc403643423)

[- Réfractaires aux traitements conventionnels : 24](#_Toc403643424)

[Critères de non-inclusion : 25](#_Toc403643425)

[6 Traitement administré aux personnes qui se prêtent à la recherche 26](#_Toc403643426)

[6.1 Description des traitements nécessaires à la réalisation de la recherche 26](#_Toc403643427)

[6.2. Principaux effets indésirables du rituximab (Mabthera®) : 26](#_Toc403643428)

[6.3. Schéma thérapeutique 27](#_Toc403643429)

[Dose de Rituximab : 27](#_Toc403643430)

[Prémédication : 27](#_Toc403643431)

[Traitements concomitants : en fonction du contexte : 27](#_Toc403643432)

[Evaluation de la toxicité du Rituximab 28](#_Toc403643433)

[Règles d’arrêt (transitoires ou définitives) du Rituximab 28](#_Toc403643434)

[7 Statistiques 29](#_Toc403643435)

[7.1. Plan d’analyse statistique et justification du nombre de patients à inclure : 29](#_Toc403643436)

[7.2. Analyses statistiques 29](#_Toc403643437)

[8. Aspects réglementaires : 30](#_Toc403643438)

[8.1. Méthodes et calendrier prévus pour mesurer, recueillir et analyser les paramètres d'évaluation de la sécurité 30](#_Toc403643439)

[8.1.1.Comité de pilotage 30](#_Toc403643440)

[8.1.2. Comité de surveillance indépendant 30](#_Toc403643441)

[8.2. Description des paramètres d’évaluation de la sécurité 30](#_Toc403643442)

[8.3. Procédures mises en place en vue de l'enregistrement et de la notification des évènements indésirables 31](#_Toc403643443)

[8.3.1 Evènements indésirables non graves : 31](#_Toc403643444)

[8.3.2 Evènements indésirables graves (EIG) : 31](#_Toc403643445)

[8.4. Modalités et durée du suivi des personnes suite à la survenue d'évènements indésirables 33](#_Toc403643446)

[9 DROIT D’ACCES AUX DONNEES ET DOCUMENTS SOURCE 33](#_Toc403643447)

[10 CONTROLE ET ASSURANCE DE LA QUALITE 34](#_Toc403643448)

[10.1 Procédures de monitoring 34](#_Toc403643449)

[10.2 Transcription des données dans le cahier d’observation 34](#_Toc403643450)

[11. CONSIDERATIONS LEGALES ET ETHIQUES 35](#_Toc403643451)

[11.1 Demande d’autorisation auprès de l’Afssaps 35](#_Toc403643452)

[11.2 Demande d’avis au Comité de Protection des Personnes 35](#_Toc403643453)

[11.3 Modifications 36](#_Toc403643454)

[11.4 Déclaration CNIL 36](#_Toc403643455)

[11.5 Note d’information et Consentement éclairé 37](#_Toc403643456)

[11.6 Rapport final de la recherche 37](#_Toc403643457)

[12. TRAITEMENT DES DONNEES ET CONSERVATION DES DOCUMENTS ET DES DONNEES RELATIVES A LA RECHERCHE 37](#_Toc403643458)

[13. ASSURANCE ET ENGAGEMENT SCIENTIFIQUE 38](#_Toc403643459)

[13.1 Assurance 38](#_Toc403643460)

[13.2. Engagement scientifique 38](#_Toc403643461)

[14 REGLES RELATIVES A LA PUBLICATION 38](#_Toc403643462)

[15. Références 40](#_Toc403643463)

# 1- SYNOPSIS

#

Rituximab (anticorps monoclonal anti-CD20 dépléteur des lymphocytes B) pour le traitement des myopathies inflammatoires (MI) réfractaires avec auto-anticorps spécifiques et des myasthénies généralisées réfractaires. « FORCE » / P 05 12 04

**Investigateur Coordonnateur :** Pr Olivier Benveniste, Service de Médecine Interne, Hôpital Pitié Salpêtrière, Paris

Autres centres : Hôpital Pitié-Salpêtrière (Médecine interne 2, Rhumatologie, Institut de Myologie, Réanimation Neurologique), Hôpital Cochin, Garches, Hôpital St Louis, Hôpital K Bicêtre, CH Lyon-Sud, Lille, Nice, Rouen, Marseille

**Traitement étudié :** Rituximab 1000 mg, 2 fois (aux jours 0 et 14), suivi d’une injection unique (1000 mg) 6 mois après la 2è injection (mois 6,5 +/- 8 jours).

**Objectif principal de l’étude :** Evaluer l’efficacité du rituximab pour traiter des patients atteints de Myopathies Inflammatoires associées à des auto-anticorps (AAC) spécifiques (anti-synthétases ou anti-SRP), ou de myasthénies (avec anti-AchR), réfractaires aux traitements conventionnels.

**Critères de sélection :**

Patients des 2 sexes, âgés de 18 à 70 ans, atteints de MI primitives (définies selon la définition du “119^th^ European Neuromuscular Centre workshop”) ou de myasthénies généralisées (définies selon le “Texas Clinical Classification System”) associées à la présence d’AAC spécifiques (anti-synthétases (JO1, PL7 ou PL12) ou anti-SRP pour les MI, et anti-AchR pour les myasthénies) et réfractaires aux traitements conventionnels.

**Nombre de patients :**36 patients :

- 24 patients présentant une MI primitive (12 avec des AAC anti-synthétases, 12 avec anti-SRP),

- 12 avec une myasthénie généralisée.

**Durée de la recherche** : La durée de l'étude est de 5 ans : durée d'inclusion de 3 ans et un suivi ou participation de 18 mois (36 + 18 = 54 mois). Les 6 mois restant sont prévus pour l'analyse statistique.

**Durée de participation**: La durée de participation par sujet est de 18 mois.

**Méthode :** étude pilote, ouverte, prospective, multicentrique, de phase II

**Critères d'évaluation :**

Principal : Testing musculaire (échelle de Kendall pour les MI et score des myasthénies) à M12

Secondaires: Testing musculaire à J21, M 6,5 et M18

Qualité de vie (questionnaire SF 36)

Evolution du taux des CPK et du titre des AAC

Tolérance: évènements indésirables

**Résultats attendus:**

Si ce traitement est un succès pour au moins 6 patients par groupe (c’est-à-dire 6 patients présentant une MI avec anti-synthétases, 6 présentant une MI avec anti-SRP et 6 présentant une myasthénie généralisée), il sera alors possible de conclure que le taux de réponse est supérieur à 25% (inférieur à 90%, intervalle de confiance pour la réponse observée à 50%).

**Déroulement de l'essai:**

S-4 à S-1 : procédure diagnostique, signature du consentement, examen clinique avec testing, analyse biologique.

J-1 : examen clinique avec testing, qualité de vie, capacité vitale pour les myasthénies, analyse biologique avec dosage des ß-hCG (pour les femmes),.

J0 : 1^ère^ administration de rituximab, analyse biologique, examen clinique, tolérance

J1 : examen clinique, tolérance.

J7 : analyse biologique, examen clinique, tolérance

J14 : 2^ème^ administration de rituximab, analyse biologique, examen clinique, tolérance

J21 : examen clinique avec testing, analyse biologique, tolérance

M6,5: 3^ème^ administration de rituximab, examen clinique avec testing, analyse biologique avec dosage des ß-hCG (pour les femmes), tolérance

M12 : examen clinique avec testing, analyse biologique, tolérance, qualité de vie, capacité vitale pour les myasthénies

M18 : examen clinique avec testing, analyse biologique, tolérance

Les études ancillaires proposées nécessiteront la participation des patientes à S-4 à S-1, J-1, J21, M6,5, M12 et M18.

**Schéma de l’étude**

|  | S-4 à S-1 | J-1 | J0 | J1 | J7 | J14 | J21 | M6 | M12 | M18 |
| --- | --- | --- | --- | --- | --- | --- | --- | --- | --- | --- |
| Procédure diagnostique (avec ou sans biopsie musculaire) | ⦁ |  |  |  |  |  |  |  |  |  |
| Signature du consentement | ⦁ |  |  |  |  |  |  |  |  |  |
| Rituximab (1000 mg) |  |  | ⦁ |  |  | ⦁ |  | ⦁ |  |  |
| Examen clinique | ⦁ | ⦁ | ⦁ | ⦁ | ⦁ | ⦁ | ⦁ | ⦁ | ⦁ | ⦁ |
| Testing neuro-musculaire | ⦁ | ⦁ |  |  |  |  | ⦁ | ⦁ | ⦁ | ⦁ |
| Qualité de vie (SF 36) |  | ⦁ |  |  |  |  |  |  | ⦁ |  |
| CV (myasthénie) |  | ⦁ |  |  |  |  |  |  | ⦁ |  |
| Biologie standard (CPK …) | ⦁ | ⦁ |  |  | ⦁ | ⦁ | ⦁ | ⦁ | ⦁ | ⦁ |
| ß-hCG (pour les femmes) |  | ⦁ |  |  |  |  |  | ⦁ |  |  |
| Evts intercurrents |  |  | ⦁ | ⦁ | ⦁ | ⦁ | ⦁ | ⦁ | ⦁ | ⦁ |
| Etudes ancillaires | ⦁ | ⦁ |  |  |  |  | ⦁ | ⦁ | ⦁ | ⦁ |

# JUSTIFICATION SCIENTIFIQUE ET DESCRIPTION GENERALE DE LA RECHERCHE:

## 2.1 Généralités

Le Rituximab est un anticorps monoclonal chimérique spécifique du CD20 humain, qui cible donc les lymphocytes B. Il a été initialement développé (et a d’ailleurs reçu l’AMM) pour le traitement des lymphomes B (1). Dans cette indication, plus de 300 000 patients ont déjà reçus cette drogue avec une très bonne tolérance (1).

Plus récemment, de nouveaux essais ont suggéré l’intérêt de cette molécule pour le traitement de maladies auto-immunes médiées par une réponse humorale. En effet, le rôle central des cellules B a été montré non seulement pour la sécrétion d’auto-anticorps ou la présentation d’auto-antigènes, mais aussi pour la production de cytokines pro-inflammatoires et la régulation des fonctions des cellules d’endritiques (2). Le Rituximab pourrait donc être considéré comme une nouvelle biothérapie pour les maladies auto-immunes, tout spécialement pour les patients réfractaires aux traitements conventionnels.

A ce jour, quelques équipes ont déjà commencé à l’utiliser pour traiter par exemple, la polyarthrite rhumatoïde réfractaire aux corticoïdes et aux immunosuppresseurs conventionnels (2) dans un essai prospectif, contrôlé, randomisé, incluant 161 patients, ou bien dans des études ouvertes concernant d’autres maladies auto-immunes, comme : 25 patients avec un purpura thrombocytopénique idiopathique (3), 13 patients avec un lupus érythémateux (4), 11 patients avec des vascularites systémiques (5), 6 patients avec un syndrome de Gougerot Sjögren primitif (4) ou 6 patients atteints de dermatomyosite (6). Plus de 70 % d’efficacité est alors observée (2, 4, 6) renforçant l’intérêt de cette biothérapie. Le Rituximab vient d’ailleurs d’obtenir l’AMM dans l’indication de la polyarthrite rhumatoïde.

En tant que centre de référence pour les maladies neuromusculaires, nous avons un recrutement important de patients avec des myopathies inflammatoires et des myasthénies généralisées. Bien que la physiopathologie de ces deux maladies soit différente (voir plus loin), leur prise en charge thérapeutique est très proche.

Dans les formes sévères de ces deux maladies, la mortalité peut atteindre 70 % en l’absence de traitement efficace (7, 8). Les traitements habituels dans ces deux maladies sont les immunosuppresseurs débutant initialement par les corticoïdes. Néanmoins, plus de 70 % des patients traités par corticoïdes présentent une réponse incomplète, voire pour 10 à 30 % d’entre eux, l’absence de réponse (8, 9).

Le traitement par corticoïdes est également associé à d’importants effets secondaires, comme la surcharge pondérale, l’intolérance au glucose, la cataracte, l’ostéoporose, la myopathie induite par les corticoïdes et le retard de croissance chez les enfants.

Chez les patients qui présentent une réponse incomplète ou des effets secondaires importants aux corticoïdes, l’ajout d’immunosuppresseurs est la règle, tel que l’azathioprime, le methotrexate, le cyclophosphamide et/ou la ciclosporine A. L’efficacité de ces molécules a été montrée dans des essais cliniques comprenant un nombre limité de patients, et avec une efficacité parfois modérée (8, 10). Les immunoglobulines intraveineuses ou les échanges plasmatiques peuvent également apporter un effet bénéfique dans ces formes réfractaires de maladies, bien que leur efficacité soit de courte durée (11, 12).

Puisque certains patients restent réfractaires à tous ces traitements, et comme certains d’entre eux vont développer des effets secondaires, de nouvelles approches thérapeutiques sont donc nécessaires.

**Nous proposons donc d’évaluer dans une étude pilote, ouverte, prospective, multicentrique, de phase II, l’efficacité du Rituximab pour le traitement de patients avec une myopathie inflammatoire associée à des auto-anticorps spécifiques ou de patients atteints de myasthénie généralisée, réfractaires aux traitements conventionnels.**

Cette première étape est nécessaire avant un essai de phase III, comparant les corticoïdes au Rituximab dans ces indications.

## 2.2 Situation de l’équipe de l’investigateur coordonnateur et des autres centres

Le service de Médecine Interne 1 (Professeur Serge HERSON) est associée à l’Institut de Myologie de la Pitié-Salpétrière (Professeur Bruno EYMARD) et est labellisé Centre de référence pour les maladies rares neuromusculaires.

Dans ce centre, plus de 300 patients avec une myopathie inflammatoire et 100 avec une myasthénie généralisée sont suivis. Le service de Médecine Interne comprend en outre, le centre de thérapie génique constitué d’une chambre de niveau de confinement P2 et d’un laboratoire P2 attenant (dirigé par le Professeur David KLATZMANN).

Des essais de biothérapie ont d’ores et déjà eu lieu dans cette structure (13, 14).

L’investigateur coordonnateur (Olivier BENVENISTE) travaille comme clinicien dans les deux départements, il a également développé une thématique de recherche sur les myopathies inflammatoires dans le laboratoire de David KLATZMANN à la Pitié-Salpétrière (15, 16) et il a mené des travaux de recherche sur la myasthénie à Oxford dans le laboratoire d’Angela VINCENT (17).

Cet essai représente les efforts coordonnés d’investigateurs de différents services (médecine interne, rhumatologie, neurologie, myologie).

Ces investigateurs ont accumulé au cours des années passées une expertise complémentaire dans la connaissance physiopathologie et le développement d’essais cliniques pour les maladies auto-immunes.

Cette étude concerne 2 pathologies distinctes (les myopathies inflammatoires, et la myasthénie généralisée). Néanmoins, le schéma thérapeutique et le suivi seront communs.

## 2.3 Description des pathologies

## Myopathies inflammatoires

## Myopathies inflammatoires réfractaires associées à des auto-anticorps spécifiques.

Les myopathies inflammatoires peuvent être primitives (idiopathiques) ou secondaires à d’autres connectivites. Mais toutes sont caractérisées par la survenue d’une faiblesse musculaire progressive (qui peut éventuellement atteindre les muscles de la déglutition et les muscles respiratoires et menacer alors le pronostic vital) et par un infiltrat inflammatoire sur la biopsie musculaire.

### Myopathies inflammatoires primitives :

Les myopathies inflammatoires primitives peuvent être classées en 5 catégories, selon leurs aspects cliniques et immuno-histologiques (7, 18) : 1) les dermatomyosites (DM), une microvascularite médiée par les anticorps caractérisées par le dépôt du complexe d’attaque membranaire C5b9 dans les capillaires du derme et du muscle entraînant des phénomènes d’ischémie ; 2) la polymyosite (PM) ; et 3) la myosite à inclusions (MI), 2 maladies associées à une hyper-expression de l’HLA de classe I sur les fibres musculaires entraînant leur lyse par des lymphocytes T CD8 positifs, cytotoxiques, dirigés contre un antigène jusque là inconnu ; 4) les myosites non spécifiques et 5) les myopathies nécrosantes à médiation immune.

Jusqu’à 50 % de ces patients présentent des auto-anticorps. Le plus fréquemment rencontrés sont aspécifiques à type de facteur anti-nucléaire non typé ou anti-RNP, anti-SSA, anti-PMScl, etc… Néanmoins, des auto-anticorps spécifiques associés aux myopathies inflammatoires sont retrouvés dans 10 à 30 % des DM ou PM et plus rarement chez les MI. Les plus évocateurs de myopathies inflammatoires sont dirigés contre la synthétase des amino-acyl-ARNt qui connectent chaque amino-acyl à son ARN de transfert durant la synthèse protéique. Il s’agit des anticorps anti-JO-1 (histidyl – ARNt) (19), PL-7 (threonyl - ARNt), PL-12 (Alanine – ARNt), OJ (isoleucil - ARNt), et EJ (glycyl – ARNt). Ils sont donc retrouvés dans 10 à 30 % des cas, le plus souvent chez des patients atteints de polymyosites avec des mains de mécaniciens, des arthrites, des pneumopathies interstitielles, et un phénomène de Raynaud constituant ainsi le syndrome des anti-synthétases (20).

Cette forme particulière de myopathie inflammatoire est sévère, du fait de sa localisation aux poumons et de la résistance aux traitements corticoïdes.

Plus récemment, d’autres auto-anticorps spécifiques ont mis en évidence chez 4 à 6 % des patients atteints de myopathie inflammatoire et dirigés contre le « signal recognition particle (SRP) » (21). De nouveau, ils représentent un syndrome particulier, associant une myopathie très rapidement évolutive, un haut niveau des CPK, une atteinte myocardique fréquente, un haut degré de nécrose régénération au niveau des fibres musculaires et une résistance aux traitements par les corticoïdes (21).

En juillet 2005, une nouvelle classification des myopathies inflammatoires a été proposée (22), basée sur des caractéristiques clinico-sérologiques des patients. Les auteurs ont proposé de classer les patients comme ayant une pure PM ou une pure DM, une myosite associée au cancer, ou une myosite de chevauchement. Cette dernière forme est la plus fréquemment retrouvée dans leur cohorte (70,5 %). Elle est définie sur la clinique par l’existence de signes extra musculaires ou cutanés (syndrome de Raynaud, arthrite, sclérodactylie, mains de mécanicien, fièvre, pneumopathie interstitielle, HTAP, syndrome pseudo-occlusif…) et sur des critères sérologiques (présence d’anticorps anti-synthétases, anti-SRP, anti-nucléoporine, et/ou auto-anticorps associés aux sclérodermies systémiques).

Là, de nouveau, les anticorps anti-synthétase ou anti-SRP sont des marqueurs d’une myopathie inflammatoire chronique et réfractaire aux traitements par les corticoïdes (22).

Les myopathies inflammatoires associées à des auto-anticorps spécifiques, anti-synthétase ou anti-SRP sont donc cliniquement particulières en terme de sévérité et de corticorésistance.

Plusieurs types d’arguments, sont en faveur de la médiation humorale :

1. du syndrome des anti-synthétases où la biopsie musculaire faite chez 11 patients avec des anti-JO 1 est différente de celle décrite pour les PM/DM ou MI (23).
   1. elle contient des régions de fragmentation et de raréfaction périmysiale du tissu conjonctif, où les cellules inflammatoires sont localisées dans le périmysium de façon prédominante,
   2. la plupart des cellules inflammatoires sont des macrophages et en de rares isolats focaux dans le périmysium, on retrouve une population contenant quelques lymphocytes,
   3. les anticorps anti-JO1 sont détectés dans le sérum des patients, bien que leurs rôles physiopathologiques restent à ce jour incertain (23).
2. Pour le syndrome des « anti-SRP » :
   1. Il n’y a pas d’argument pour que des cellules T CD8+ cytotoxiques puissent jouer là un rôle, puisqu’il n’y a pas d’hyper-expression sur les fibres musculaires de l’HLA de classe I et également les analyses en « immunoscopes » ont révélé un répertoire lymphocytaire normal chez un de nos patients (24), comme cela a été rapporté au cours de la DM, ce qui contraste avec les sévères perturbations du répertoire lymphocytaire T, observé au cours de la PM qui est donc une maladie médiée par les lymphocytes TCD 8+ (15).
   2. Le dépôt du complexe d’attaque membranaire C5 b9 est présent à la fois au niveau des capillaires endomysiaux et des capillaires non nécrotiques du sarcolemne (24).
   3. Les anticorps anti-SRP sont détectés dans le sérum des patients, bien que, également à ce jour, leur rôle physio-pathologique reste incertain (25).

La synthèse de ces résultats constitue donc un rationnel suffisant pour penser qu’il y ait une médiation humorale rentrant dans la physiopathogènie du syndrome des anti-synthétases et des anti-SRP et nous incite donc à traiter ces patients par le Rituximab.

### Myopathies inflammatoires secondaires :

De plus, certaines myopathies inflammatoires sont secondaires à d’autres connectivites comme la sclérodermie, le syndrome de Gougerot Sjögren, le lupus érythémateux… Dans ce contexte, la myopathie est associée aux caractéristiques spécifiques de la connectivite sous-jacente (y compris la présence d’auto-anticorps spécifiques). De la sorte, elle pourrait être également accessible à un traitement par le Rituximab (4), mais cette population de patients n’étant pas homogène, nous l’avons exclu de cette étude.

## Résultats acquis :

Six patients atteints de DM, parmi lesquels 2 avaient des anticorps anti-JO1 (6) et 2 autres atteints de PM avec également des anticorps anti-JO1 (4) ont été traités par Rituximab avec succès et ont été rapportés récemment dans la littérature. Nous avons également rapportés 2 autres cas de myopathies inflammatoires avec des anticorps anti-SRP où nous avons observé un effet spectaculaire du Rituximab (24).

## Myasthénie généralisée

La myasthénie est une maladie auto-immune associée à des auto-anticorps anti-récepteurs à l’acetylcholine (anti-RAch) chez 80% des patients ou à des anticorps anti-kinase muscle spécifique (anti-Musk, 10 % des patients) (8). Ces auto-anticorps induisent une interruption de la transmission neuro-musculaire. Cette dernière est caractérisée par une fatigabilité musculaire et un décrément à l’EMG après des stimulations répétitives. Chez environ 20 % des patients, il s’y associe un thymome malin qui nécessite une ablation chirurgicale.

La caractéristique clinique la plus remarquable est la fatigabilité et la faiblesse musculaire. Elle touche différents muscles : le plus souvent les muscles oculomoteurs, bulbaires, respiratoires puis les ceintures. La sévérité est très variable, allant de formes minimes purement oculaires, jusqu’à des formes sévères généralisées. La myasthénie est considérée comme généralisée quand des muscles extra-oculaires sont affectés. La proportion de patients avec des formes généralisées varie de 40 à 60 % et jusqu’à 10 % des patients vont présenter, à un moment donné de leur maladie, une atteinte respiratoire sévère (encore appelée crise myasthénique).

Les patients avec des formes généralisées, non traités ou de façon insuffisamment efficace, sont à risque d’une détérioration les conduisant vers une crise myasthénique, qui lorsqu’elle survient, peut les conduire en réanimation pour une ventilation mécanique (27). Les immunoglobulines polyvalentes et les échanges plasmatiques sont alors les traitements de ces exacerbations aigues.

En complément des drogues anti-cholynestérasiques, les patients atteints des formes généralisées doivent être traités au long cours par des corticostéroïdes et des drogues immuno-suppressives (azathioprine, mycophénolate mofetil voire cyclosporine, (28-31)). Entre 5 à 10 % des patients restent néanmoins réfractaires à ces drogues (32-33). D’autres immuno-suppresseurs peuvent être alors essayés comme le cyclophosphamide (34), le tacrolimus, ou l’etanercept (36) mais leur efficacité n’a jamais été montrée sur la base d’essais randomisés. Les anti-CD20 ont été récemment proposés comme traitement au long cours pour des myasthénies réfractaires (voir plus bas).

De nombreux arguments plaident en faveur de la nature humorale de la myasthénie.

1. La présence des auto-anticorps conduit à la disparition des récepteurs à l’acétylcholine réduisant l’efficacité de la transmission neuro-musculaire et delà, l’apparition d’une faiblesse musculaire et d’une fatigabilité (8).
2. Le transfert transplacentaire de ces auto-anticorps chez des femmes atteintes de myasthénie peut entraîner une myasthénie fœtale et/ou néonatale sévère chez leurs enfants (8).
3. Les échanges plasmatiques produisent un effet spectaculaire chez les myasthéniques (37), bien que cette amélioration ne soit pas permanente. Le bénéfice clinique est corrélé avec la réduction du titre des anticorps anti-RAch.
4. Le plasma ou les IgG purifiées de patients sont capables de transférer la maladie à la souris (38).
5. L’immunisation contre le récepteur à l’acétylcholine conduit chez l’animal à un syndrome myasthénique (40).
6. Les IgG humaines sont détectées au niveau des plaques motrices chez les patients atteints (39).

L’ensemble de ces arguments permet d’établir que c’est la présence des auto-anticorps anti-Rach qui conduit à la myasthénie. Il s’agit donc là, d’un rationnel suffisant pour prouver la nature humorale de cette maladie et nous inciter à traiter ces patients par le Rituximab.

## Résultats acquis :

2 cas de myasthénie réfractaire ont été traités avec succès par le Rituximab (41-42).

Nous avons également récemment traité trois patients myasthéniques avec ce médicament, avec de bons résultats pour les 2 premiers (poster CA170, 53ème congrès de la SNFMI, Nancy 14-17 juin 2006), le 3^ème^ est en cours de traitement.

# 3 OBJECTIFS DE LA RECHERCHE

## 3.1 Hypothèses cliniques

Evaluer dans une étude pilote, ouverte, prospective, multicentrique, de phase II, l’efficacité du Rituximab pour le traitement de patients avec une myopathie inflammatoire associée à des auto-anticorps spécifiques ou de patients atteints de myasthénie généralisée, réfractaires aux traitements conventionnels et ainsi menacé dans leur pronostic fonctionnel voire vital.

## 3.2. Objectifs

Objectif principal :

Evaluer l’efficacité du Rituximab dans le traitement de patients avec une myopathie inflammatoire associée à des auto-anticorps spécifiques ou de patients atteints de myasthénie généralisée, réfractaires aux traitements conventionnels**.**

Concernant les patients avec une **myopathie inflammatoire**, une amélioration, pour un sujet donné, de la force musculaire, définie par au moins 2 points gagnés sur l’échelle de Kendall dans au moins 2 groupes musculaires différents (soit un gain d’au moins 4 points) (18) (voir annexe 1), au 12ème mois, est l’objectif d’efficacité retenu.

Si ce traitement est un succès pour au moins 12 patients (6 avec des AAC anti-synthétases, ­6 avec des AAC anti-SRP) il sera alors possible de conclure que le taux de réponse est supérieur à 25% (inférieur à 90%, intervalle de confiance pour la réponse observée à 50%).

Concernant les patients atteints de **myasthénie généralisée**, une amélioration, pour un sujet donné, de la force musculaire, définie par une augmentation d’au moins 20 points du score MMG (47, 48) (voir annexe 2) au 12^ème^ mois, est l’objectif d’efficacité retenu.

Si ce traitement est un succès pour au moins 6 patients, il sera alors possible de conclure que le taux de réponse est supérieur à 25% (inférieur à 90%, intervalle de confiance pour la réponse observée à 50%).

Objectifs secondaires :

**Myosites**

- - Une amélioration de la force musculaire, définie sur l’échelle de Kendall par au moins 2 points gagnés dans 2 groupes musculaires différents (soit un gain d’au moins 4 points) (18) au jour 21, au 6^ème^ mois et à M18.
  - Une amélioration de la qualité de vie (auto questionnaire SF 36).
  - Une décroissance du taux des CPK.
  - Une décroissance du taux des auto-anticorps
  - Une amélioration de l’activité extra-musculaire de la maladie, comme l’atteinte pulmonaire sur les EFR.
  - Une diminution des doses ou de la quantité des traitements immuno-modulateurs associés.

**Myasthénies généralisées**

- Une amélioration de la force musculaire, définie par une augmentation d’au moins 20 points du score MMG (47, 48) au jour 21, au mois 6 et à M18.

- Une amélioration du statut post interventionnel MGFA (45).

- Une amélioration du score ADLS

- - Une amélioration de la qualité de vie (auto questionnaire SF 36).

- Une diminution du nombre des exacerbations cliniques qui nécessitent soit une hospitalisation ou un changement dans les doses de corticoïdes.

- Une diminution des doses des corticoïdes et des traitements associés

- Une décroissance du taux des auto-anticorps.

- Une amélioration de la capacité vitale

## 3.3. Bénéfices / risques

Les patients devant être inclus dans l’étude (cf. critères d’inclusions) ont leur pronostic vital engagé du fait 1) de la gravité de leurs maladies (myosite ou myasthénie) qui touchent la musculature oropharyngée et respiratoire, et 2) de la résistance aux différents immunomodulateurs testés. Le rituximab - dont l’efficacité va être testée ici – est utilisé en thérapie de sauvetage. Dans notre expérience, l’efficacité du rituximab ne se manifeste qu’au bout de 8 à 16 semaines. Face à ces patients gravement atteints et pour couvrir la phase « silencieuse » du rituximab, il ne serait pas éthique de stopper tout traitement immunomodulateur concomitant. C’est pourquoi, à la discrétion de l’investigateur, les traitements immunomodulateurs associés sont autorisés et même recommandés. Ainsi, le maintien des corticoïdes en association avec le methotrexate, le cyclophosphamide, l’azathioprine, le mycophenolate mofetil ou des immunoglobulines polyvalentes est recommandé. En revanche, on tentera de limiter les traitements associés : on pourra maintenir la corticothérapie et l’un des traitements immunosuppresseurs sus cités (prescrit si possible depuis plus de 6 mois), auquel on ajoutera le rituximab, mais on évitera les associations multiples. La possibilité d’effets indésirables graves a été prise en compte par les investigateurs après notamment des échanges avec le laboratoire Roche qui fournira le rituximab. La balance bénéfice risque penche en faveur de ces associations. Néanmoins, un **comité** **de surveillance indépendant** (cf. § 8 aspects réglementaires) chargé de la surveillance de tous les effets indésirables se réunira après l’inclusion des 4 premiers patients. Il aura à prendre la décision de la poursuite de l’essai. Un des principaux bénéfices attendus en cas d’efficacité du rituximab, outre l’amélioration clinique des patients, sera de pouvoir alléger progressivement la charge des traitements associés chez ces patients initialement en impasse thérapeutique.

# 4 CONCEPTION DE LA RECHERCHE

## 4.1. Critères d’évaluation

**Myosites**

#### Critère principal :

L’évolution de la force musculaire à 12 mois, évaluée par les scores sur l’échelle de Kendall des différents groupes musculaires (18) (voir annexe 1), est le critère principal : c’est, pour un sujet donné, la différence entre les scores de chaque groupe musculaire sur l’échelle de Kendall à M12 et les scores à J0.

#### Critères secondaires :

- - force musculaire définie sur l’échelle de Kendall (voir annexe 1) à J21, M6,5 (+/-8 jours) et M18
  - qualité de vie (auto questionnaire SF 36) (voir annexe 3)
  - taux des CPK.
  - taux des auto-anticorps
  - activité extra-musculaire de la maladie, comme l’atteinte pulmonaire sur les EFR.
  - doses ou quantité des traitements immuno-modulateurs associés.

Critères d’évaluation de la tolérance :

- - recueil des évènements intercurrents
  - prise de la pression artérielle, fréquence cardiaque
  - numération sanguine, enzymes hépatiques, etc, pour la surveillance biologique du traitement

**Myasthénies généralisées**

#### Critère principal :

L’évolution de la force musculaire à 12 mois par le score MMG (47, 48) (voir annexe 2), est le critère principal : c’est, pour un sujet donné, la différence entre son score MMG à M12 et son score à J0.

#### Critères secondaires :

- force musculaire définie par le score MMG (47, 48) au jour 21, au mois 6 et à M18.

- statut post interventionnel MGFA (45).

- score ADLS

- - qualité de vie (auto questionnaire SF 36) (voir annexe 3)

- nombre des exacerbations cliniques qui nécessitent soit une hospitalisation ou un changement dans les doses de corticoïdes.

- doses des corticoïdes et des traitements associés

- taux des auto-anticorps.

- capacité vitale

Critères d’évaluation de la tolérance :

- - recueil des évènements intercurrents
  - prise de la pression artérielle, fréquence cardiaque
  - numération sanguine, enzymes hépatiques, etc, pour la surveillance biologique du traitement

## 4.2. Description de la méthodologie

Il s’agit d’une une étude pilote, ouverte, prospective, multicentrique, de phase II.

36 patients au total seront inclus répartis comme suit : 24 patients présentant une myosite (12 atteints de myosite avec anti synthétase et 12 atteints de myosite avec anti SRP) et 12 patients atteints d’une myasthénie généralisée.

## 4.3. Durée de participation et description de la chronologie du suivi des patients

## La durée prévisible des inclusions sera de 2 3 ans. La durée de participation des patients sera de 18 mois et la durée totale de l’étude de 4 5 ans. Les patients ne pourront pas participer à une autre recherche biomédicale pendant les 18 mois de participation à l’étude.

### Visite de pré-inclusion (S-4 à S-1) –Hospitalisation de jour

Procédure de diagnostic : les patients ayant une myopathie inflammatoire potentielle associée à des auto-anticorps spécifiques ou une myasthénie généralisée auront été informés de l’étude au préalable. Après signature du consentement éclairé, ils seront pré-inclus et subiront la procédure de diagnostic suivante afin de confirmer ou d’infirmer leur inclusion :

**Myosites**

1. Examen clinique avec réalisation du testing selon l’échelle de Kendall (18)
2. Echocardiographie
3. EMG
4. IRM musculaire.
5. Scanner thoracique et EFR.
6. Scanner corps entier et adjonction en fonction du contexte clinique d’examens endoscopiques.
7. Examens biologiques usuels (NFS, ionogramme sanguin, bilan hépatique complet, CRP, CPK) et dosage pondéral des immonoglobulines (IgG et IgM).
8. Sérologie pour le VIH, l’HBV, l’HCV, l’HTLV1
9. Réalisation d’une biopsie musculaire, si elle n’avait pas été faite auparavant
10. Examens biologiques propres aux études ancillaires.

**Myasthénies généralisées**

1. Examen clinique avec réalisation du testing MMG et MGFA (44, 45).
2. Echocardiographie
3. EMG.
4. EFR.
5. Scanner thoracique (recherche d’un thymome).
6. Examens biologiques usuels (NFS, ionogramme sanguin, bilan hépatique complet, CRP, CPK) et dosage pondéral des immonoglobulines (IgG et IgM).
7. Sérologie pour le VIH, l’HBV, l’HCV, l’HTLV1
8. Examens biologiques propres aux études ancillaires

### Période d’inclusion : Jour – 1(J-1) au jour + 2 (J2).

### Myosites et myasthénies

**Jour – 1 (J-1)** :

Examen clinique avec réalisation d’un testing musculaire et examens de biologie standard (NFS, ionogramme sanguin, bilan hépatique complet (gamma GT ; phosphatase alcaline, ASAT/ALAT, Bilirubine), CPK, ß-hCG pour les femmes) et propres aux études ancillaires ; qualité de vie, capacité vitale pour les myasthénies.

**Jour 0 (JO) à Jour 2 (J2)** :

Perfusion de la première dose de rituximab (1 000 mg) après réalisation des mesures préventives associées, voir détail en annexe 2.

Après la première injection de rituximab : examens cliniques avec prise de la pression artérielle, la fréquence respiratoire, de la saturation en oxygène au doigt, de la température toutes les 15 minutes durant les 2 premières heures, après le début de la perfusion, puis toutes les 2 heures pendant les 8 heures suivantes et finalement 3 fois par jour jusqu’à J2. (Se référer à la fiche infirmière).

Recueil des évènements intercurrents et des médications associées

### Période de suivi : Jours 7, 14, 21 et mois 6,5 (+/-8 jours), 12 et 18 :

**J7 :**

Examen clinique et examens biologiques standards (NFS, ionogramme sanguin, bilan hépatique complet, CPK)

Recueil des évènements intercurrents et des médications associées.

**J14 :**

Examen clinique et examens biologiques standards (NFS, ionogramme sanguin, bilan hépatique complet, CPK) avant la perfusion.

Perfusion de la deuxième dose de rituximab (1 000 mg) après réalisation des mesures préventives associées.

Après l’injection de rituximab : examens cliniques avec prise de la pression artérielle, la fréquence respiratoire, de la saturation en oxygène au doigt, de la température toutes les 15 minutes durant les 2 premières heures, après le début de la perfusion, puis toutes les 2 heures pendant les 8 heures suivantes (Se référer à la fiche infirmière).

Recueil des évènements intercurrents et des médications associées

**J21**:

Examen clinique avec testing neuro-musculaire et examens biologiques standards (NFS, ionogramme sanguin, bilan hépatique complet, CPK) et propres aux études ancillaires.

Recueil des évènements intercurrents et des médications associées.

**M 6,5 (+/-8 jours)** :

Examen clinique avec testing neuro-musculaire (fait avant la perfusion), dosage ß-hCG, examens biologiques standards (NFS, ionogramme sanguin, bilan hépatique complet, CPK) et propres aux études ancillaires.

Perfusion d’une dose de maintenance de rituximab (1 000 mg).

Après l’injection de rituximab : examens cliniques avec prise de la pression artérielle, la fréquence respiratoire, de la saturation en oxygène au doigt, de la température toutes les 15 minutes durant les 2 premières heures, après le début de la perfusion, puis toutes les 2 heures pendant les 8 heures suivantes et finalement 3 fois par jour pendant 48h (Se référer à la fiche infirmière).

Recueil des évènements intercurrents et des médications associées.

**M12 (+/-8 jours)** :

Examen clinique avec testing neuro-musculaire et examens biologiques standards (NFS, ionogramme sanguin, bilan hépatique complet, CPK) et propres aux études ancillaires.

Évaluation neuromusculaire. EMG et IRM musculaire (Myosite seule)

EFR

Qualité de vie

Recueil des évènements intercurrents et des médications associées

**M18 (+/-8 jours)** **: fin du suivi**

Dernier examen clinique avec testing neuro-musculaire et examens biologiques standards (NFS, ionogramme sanguin, bilan hépatique complet, CPK) et propres aux études ancillaires.

Recueil des évènements intercurrents et des médications associées

### Schéma de l’étude :

|  | S-4 à S-1 | J-1 | J0 | J1 | J7 | J14 | J21 | M6 | M12 | M18 |
| --- | --- | --- | --- | --- | --- | --- | --- | --- | --- | --- |
| Procédure diagnostique (avec ou sans biopsie musculaire) | ⦁ |  |  |  |  |  |  |  |  |  |
| Signature du consentement | ⦁ |  |  |  |  |  |  |  |  |  |
| Rituximab (1000 mg) |  |  | ⦁ |  |  | ⦁ |  | ⦁ |  |  |
| Examen clinique | ⦁ | ⦁ | ⦁ | ⦁ | ⦁ | ⦁ | ⦁ | ⦁ | ⦁ | ⦁ |
| Testing neuro-musculaire | ⦁ | ⦁ |  |  |  |  | ⦁ | ⦁ | ⦁ | ⦁ |
| Qualité de vie (SF 36) |  | ⦁ |  |  |  |  |  |  | ⦁ |  |
| CV (myasthénie) |  | ⦁ |  |  |  |  |  |  | ⦁ |  |
| Biologie standard (CPK …) | ⦁ | ⦁ |  |  | ⦁ | ⦁ | ⦁ | ⦁ | ⦁ | ⦁ |
| ß-hCG (pour les femmes) |  | ⦁ |  |  |  |  |  | ⦁ |  |  |
| Evts intercurrents |  |  | ⦁ | ⦁ | ⦁ | ⦁ | ⦁ | ⦁ | ⦁ | ⦁ |
| Etudes ancillaires | ⦁ | ⦁ |  |  |  |  | ⦁ | ⦁ | ⦁ | ⦁ |

## 4.4. Règles d’arrêt définitif ou temporaire

***Sortie d'essai***

Un patient pourra sortir de l’étude sur sa propre décision (retrait de consentement du patient).

Dans la mesure du possible, une évaluation clinique et biologique au moment de la sortie (dans le mois qui suit le retrait de consentement) sera effectuée. Toute sortie d'essai ne sera pas remplacée.

Dans tous ces cas, la raison motivant la sortie d’essai sera notifiée dans le cahier d’observation.

***Interruption de traitement***

Le traitement sera interrompu pour les raisons suivantes :

- problème de tolérance tel que la poursuite du traitement s’avère impossible pour la

sécurité du patient,

- effets secondaires gênants,

- grossesse avant la 1^ère^ injection ou en cours d’étude ou toute autre raison qui rendrait le suivi impossible,

Dans tous ces cas, la raison de l'interruption du traitement sera notifiée dans le cahier d’observation.

De plus, même si le patient ne reçoit plus le traitement, il ne sera pas considéré comme sorti d'essai. Il sera suivi et évalué au terme des 18 mois.

En cas de patient ***perdu de vue***, tout sera tenté pour obtenir des nouvelles du patient (par le médecin traitant ou la famille si besoin) et si possible un rendez-vous pour consultation.

# 5 Sélection et exclusion des personnes de la recherche

## Myosites

## Critères d’inclusion :

### - hommes ou femmes de 18 ans à 70 ans ;

- pour les femmes en age de procréer, utilisation obligatoire d’une méthode contraceptive efficace pendant toute la durée de participation à l’essai, et jusqu’à un an après la dernière perfusion de rituximab.

### - myopathies inflammatoires idiopathiques définie par :

1 – Myosite comme définie par le 119^ème^ ENMC (18) :

a) Myopathie proximale avec faiblesse musculaire.

Il faut que les points a, b et c soient présents.

b) Début sub-aigu ou plus lentement progressif chez des patients de plus de 18 ans.

c) Nécrose et régénération musculaire et/ou présences d’infiltrats inflammatoires sur la biopsie musculaire.

d) Syndrome myogène à l’EMG (critère optionnel)

2 – Présence d’auto-anticorps spécifiques : anti-synthétases (anti-JO-1, anti-PL-7 ou anti-PL-12) ou anti-SRP.

### - Réfractaires aux traitements conventionnels

La résistance aux traitements conventionnels est définie par une réponse inadéquate ou des effets secondaires intolérables avec les traitements conventionnels.

Ces traitements comprennent tout d’abord les corticostéroïdes qui doivent avoir été utilisés pendant plus de 6 mois de façon continue. Mais aussi, d’autres immunosuppresseurs dont la dispensation ne doit pas avoir été modifiée (hormis de simples ajustements de doses) dans les 3 mois précédant l’inclusion. Il s’agit de l’azathioprine, le méthotrexate, le mycophenolate mofetil, le cyclophosphamide, la ciclosporine, les immunoglobulines polyvalentes, et/ou les échanges plasmatiques. Ainsi, les corticostéroides et deux au moins de ces drogues ou des ces approches thérapeutiques (utilisées séquentiellement ou en association) doivent avoir été testées sans succès avant l’inclusion, selon les délais rapportés plus haut. Une réponse inadéquate est définie comme l’absence d’amélioration et/ou la dégradation des paramètres évalués (force musculaire, capacité vitale, augmentation des CPK, etc.), malgré ces traitements conventionnels, entraînant le prescripteur à modifier ou à réintroduire d’autres traitements..

## Critères de non-inclusion :

1. Autres pathologies musculaires comme :
   1. La myosite à inclusions.
   2. La myofasciite à macrophages.
   3. Les myopathies héréditaires.
2. Myopathies inflammatoires secondaires à d’autres connectivites :
   1. Sclérodermie systémique (selon les critères de la ARA et/ou de LEROY et MEDSGER).
   2. Syndrome de Gougerot Sjögren (critères européens).
   3. Lupus érythémateux systémique (critères ACR).
   4. Polyarthrite rhumatoïde (critères ACR).
   5. Connectivite mixte.
3. Cancers ou myosites associées aux cancers (22)
4. Age inférieur à 18 ans et supérieur à 70 ans
5. Absence de signature du consentement éclairé
6. Patient non affilié au régime de sécurité sociale
7. Antécédent de réaction allergique sévère ou de réaction anaphylactique à un anticorps monoclonal murin ou humanisé, ou hypersensibilité connue à l’un des composant du rituximab.
8. Antécédent de réaction allergique sévère ou de réaction anaphylactique à la methylprednisolone.
9. Affection cardiaque ou pulmonaire sévère (à l’appréciation de l’investigateur : à documenter dans le CRF).
10. Présence d’une maladie concomitante non controlée, comme une affection du système nerveux central, rénale, hépatique, endocrinienne ou gastrointestinale, qui selon l’appréciation de l’investigateur ne doit pas permettre l’inclusion du patient.
11. Infection active de toute nature. Antécédent d’infection sévère récidivante ou chronique. Antécédent d’infection profonde à type de fasciite, abcés, ostéomyélite au cours des 52 semaines précédantes.
12. Syndrome d’immunodéficience, y compris la séropositivité pour le VIH.
13. Intervention chirurgicale programmée dans l’année suivant l’inclusion.
14. Vaccination de toute nature au cours des 28 jours précédants (il est recommandé de bien peser l’indication d’une vaccination (par exemple antigrippale pour un patient atteint d’une affection neuromusculaire) avant le traitement par rituximab et d’en évaluer ensuite soigneusement l’efficacité (qui peut être totalement perdue du fait même du mécanisme d’action du rituximab). En d’autres termes, il est recommandé d’anticiper la nécessité de vacciner les patients bien avant les 28 jours antèrieurs à l’injection du rituximab). L’usage de vaccin vivant atténué est prohibée durant toute la durée de l’étude (et au cours des 28 jours précédents).
15. Traitement avec tout agent expérimental dans un délai précédant l'inclusion inférieur à 28 jours ou à 5 demi-vies du médicament considéré (le plus long de ces deux délais est à prendre en compte).
16. Traitement préalable (dans le mois précédant l’inclusion) par une thérapie de déplétion cellulaire, de quelque nature que ce soit, comme par exemple CAMPATH, anti CD4, anti CD5, anti CD3, anti CD19, anti CD11a, anti CD22, anti-Blys /BA.
17. Positivité des ß-hCG sériques mesurées au screening ou tout autre test de grossesse positif effectué avant la première injection de rituximab.
18. Grossesse ou allaitement ou tentative de grossesse au cours de l’étude.

19. Test positif à l’**hépatite B** (à type d’antigène Hbs positif et /ou  PCR HBV positif).

Néanmoins, les patients présentant une hépatite B ancienne et guérie (AgHBS négatifs,  Ac anti-Hbc +/-, Ac anti-Hbs+) sont incluables avec une surveillance spécifique tous les 3 mois de l’Ag  Hbs et de la PCR HBV et  6 mois après la fin de l’Etude ou l’**hépatite C**.

20. Hémoglobine < 8.0 g/dL

21. Polynucléaire neutrophile <1 ,5 X 10 ³/µL.

22. Niveaux des IgG et/ou des IgM < 5.0 et 0 .40 mg /mL, respectivement.

**Myasthénies généralisées**

## Critères d’inclusion :

### - hommes ou femmes de plus de 18 ans à 70 ans

- pour les femmes en age de procréer, utilisation obligatoire d’une méthode contraceptive efficace pendant toute la durée de participation à l’essai, et jusqu’à un an après la dernière perfusion de rituximab.

### - Myasthénie généralisée définie par :

Myasthénie généralisée séropositive comme définie par la TEXAS CLINICAL CLASSIFICATION SYSTEM (43), à un stade clinique > à la classe III (IVa, IVb ou V), c'est-à-dire, avec une faiblesse sévère affectant les racines ou la musculature axiale (classe IVa), ou l’atteinte bulbaire et respiratoire (classe IVb), ou nécessitant une ventilation assistée (classe V).

1. Faiblesse sévère (classes IVa, IVb ou V) des muscles extra-oculaires quantifiée sur le score de myasthénie (MMS) dont la reproductibilité inter et intra-observateur a été démontrée (44).
2. Présence des auto-anticorps spécifiques : anti-RAch.

### - Réfractaires aux traitements conventionnels :

La résistance aux traitements conventionnels est définie par une réponse inadéquate ou des effets secondaires intolérables avec les traitements conventionnels.

Ces traitements comprennent tout d’abord les corticostéroïdes qui doivent avoir été utilisés pendant plus d’un an de façon continue. Mais aussi, d’autres immunosuppresseurs dont la dispensation ne doit pas avoir été modifiée (hormis de simples ajustements de doses) dans les 6 mois précédant l’inclusion. Il s’agit de l’azathioprine, du mycophenolate mofetil, du methotrexate, du cyclophosphamide, de la ciclosporine, des immunoglobulines polyvalentes et/ou des échanges plasmatiques. Ainsi, les corticostéroides et deux au moins de ces drogues ou des ces approches thérapeutiques (utilisées en association) doivent avoir été testées sans succès avant l’inclusion, selon les délais rapportés plus haut. Une réponse inadéquate est définie comme l’absence d’amélioration et/ou la dégradation des paramètres évalués (force musculaire, capacité vitale, etc), malgré ces traitements conventionnels, entraînant le prescripteur à modifier ou à réintroduire d’autres traitements.

## Critères de non-inclusion :

- 1. Autres syndromes myasthéniques, comme :
     - - Myasthénies non généralisées (oculaires).
       - Syndrome de Lambert Eaton.
       - Myasthénies associées à un thymome malin.
       - Syndrome myasthénique héréditaire.
  2. Age inférieur à 18 ans et supérieur à 70 ans
  3. Absence de signature du consentement éclairé
  4. Patient non affilié au régime de sécurité sociale
  5. Antécédent de réaction allergique sévère ou de réaction anaphylactique à un anticorps monoclonal murin ou humanisé, ou hypersensibilité connue à l’un des composant du rituximab.
  6. Antécédent de réaction allergique sévère ou de réaction anaphylactique à la methylprednisolone.
  7. Affection cardiaque ou pulmonaire sévère (à l’appréciation de l’investigateur: à documenter dans le CRF).
  8. Présence d’une maladie concomitante non controlée, comme une affection du système nerveux central, rénale, hépatique, endocrinienne ou gastrointestinale, qui selon l’appréciation de l’investigateur ne doit pas permettre l’inclusion du patient.
  9. Infection active de toute nature. Antécédent d’infection sévère récidivante ou chronique. Antécédent d’infection profonde à type de fasciite, abcés, ostéomyélite au cours des 52 semaines précédantes.
  10. Syndrome d’immunodéficience, y compris la séropositivité pour le VIH.
  11. Intervention chirurgicale programmée dans l’année suivant la possible inclusion.
  12. Vaccination de toute nature au cours des 28 jours précédants (il est recommandé de bien peser l’indication d’une vaccination (par exemple antigrippale pour un patient atteint d’une affection neuromusculaire) avant le traitement par rituximab et d’en évaluer ensuite soigneusement l’efficacité (qui peut être totalement perdue du fait même du mécanisme d’action du rituximab). En d’autres termes, il est recommandé d’anticiper la nécessité de vacciner les patients bien avant les 28 jours antèrieurs à l’injection du rituximab). L’usage de vaccin vivant atténué est prohibée durant toute la durée de l’étude (et au cours des 28 jours précédents).
  13. Traitement avec tout agent expérimental dans un délai précédant l'inclusion inférieur à 28 jours ou à 5 demi-vies du médicament considéré (le plus long de ces deux délais est à prendre en compte).
  14. Traitement préalable (dans le mois précédant l’inclusion) par une thérapie de déplétion cellulaire, de quelque nature que ce soit, comme par exemple CAMPATH, anti CD4, anti CD5, anti CD3, anti CD19, anti CD11a, anti CD22, anti-Blys /BA.
  15. Positivité des bêtahCG sériques mesurées au screening ou tout autre test de grossesse positif effectué avant la première injection de rituximab.
  16. Grossesse ou allaitement ou tentative de grossesse au cours de l’étude.

17.Test positif à l’**hépatite B** (à type d’antigène Hbs positif et /ou  PCR HBV positif).

Néanmoins, les patients présentant une hépatite B ancienne et guérie (AgHBS négatifs,  Ac anti-Hbc +/-, Ac anti-Hbs+) sont incluables avec une surveillance spécifique tous les 3 mois de l’Ag  Hbs et de la PCR HBV et  6 mois après la fin de l’Etude ou l’**hépatite C**.

18.Hémoglobine < 8.0 g/dL

19.Polynucléaire neutrophile <1 ,5 X 10 ³/µL.

20.Niveaux des IgG et/ou des IgM < 5.0 et 0 .40 mg /mL, respectivement.

## 6 Traitement administré aux personnes qui se prêtent à la recherche

## 6.1 Description des traitements nécessaires à la réalisation de la recherche

Le rituximab est un anticorps monoclonal chimérique murin/humain obtenu par génie génétique ; il s'agit d'une immunoglobuline glycosylée associant d'une part les régions constantes d’une IgG1 humaine et d'autre part les régions variables des chaînes légères et lourdes d'origine murine.

Un flacon à usage unique contient 500 mg/50 ml de rituximab.

Chaque ml de solution contient 10 mg de rituximab.

La solution de MabThera préparée doit être administrée en perfusion intraveineuse par une voie spécifique. La solution diluée pour perfusion I.V. ne doit pas être injectée rapidement ni en bolus.

## 6.2. Principaux effets indésirables du rituximab (Mabthera®) :

L’administration de rituximab (Mabthera®), par voie intraveineuse, peut entraîner un certain nombre d’effets indésirables. L’un de ces effets secondaires, survenant particulièrement pendant les deux premières heures suivant la première perfusion, se caractérise par de la fièvre, des frissons et des tremblements, parfois associés à une éruption urticarienne, voire à une baisse de la tension artérielle et au pire des difficultés respiratoires. Une prémédication (voir plus bas) visant à prévenir ces manifestations sera systématiquement administrée lors des injections du médicament. Par ailleurs, afin de minimiser ces réactions, des vitesses progressivement croissantes d’injection du rituximab devront être suivies lors de son administration (cf. annexe 16.6 « procédure d’administration intraveineuse du Mabthéra »).

Les autres effets indésirables possibles sont : des douleurs (abdomen, thorax, muscles, articulations, points de perfusion), des sensations de malaise, des modifications de la tension artérielle.

Tout comme les immunosuppresseurs et les corticoïdes, le rituximab (Mabthera®) expose lui aussi au risque infectieux, avec un effet potentiellement additif du fait des expositions antérieures et concomitantes à des traitements immunorégulateurs. Notamment, le risque d’infections virales graves est documenté, qu’il s’agisse de primo-infection ou de réactivations (par exemple à EBV, VZV…), ou d’exacerbations (par exemple au virus JC). En effet, 3 cas de leucoencéphalopathie multifocale progressive (LEMP), ont été rapportés. Cette infection a été observée au cours de lupus et de vascularite, où le traitement comporte aussi d’autres immunosuppresseurs. A ce jour, la relation causale entre le rituximab (Mabthera®) et la survenue de leucoencéphalopathie multifocale progressive, n’est pas établie. Néanmoins, l’apparition de symptômes neurologiques centraux devra faire évoquer le diagnostic de LEMP qui devra être étayé par la réalisation d’une IRM cérébrale ± une ponction lombaire avec recherche du virus JC par PCR.

## 6.3. Schéma thérapeutique

### Dose de Rituximab :

La dose préconisée par le laboratoire Roche, est celle retenue pour le traitement de la polyarthrite rhumatoïde (2) : c’est-à-dire, 2 injections de 1 000 mg de Rituximab à deux semaines d’intervalle.

Un traitement de maintenance afin de minimiser le risque de rechute (comme pour les lymphomes (26)) sera réalisé : une injection supplémentaire de 1 000 mg de Rituximab au 6^ème^ mois après la précédente injection (M6,5 (+/-8 jours)).

### Prémédication :

Corticoïdes : tous les patients recevront 100 mg de Methylprednisolone en iv lente, 30 minutes avant la perfusion du Rituximab afin d’en réduire les effets secondaires potentiels.

Antipyrétiques : il est recommandé que chaque patient soit prémédiqué par 1 g (iv ou per os) de Paracetamol, 30 à 60 minutes avant la perfusion du Rituximab afin d’en réduire les effets secondaires potentiels.

Antihistaminiques : il est recommandé que chaque patient soit prémédiqué par un antihistaminique H1 (per os, par ex Ceftirizine, ZYRTEC : 1 cp à 10 mg), 30 à 60 minutes avant la perfusion du Rituximab afin d’en réduire les effets secondaires potentiels.

### Traitements concomitants : en fonction du contexte :

1. les échanges plasmatiques avant l’injection de Rituximab peuvent être autorisés.
2. Une corticothérapie concomitante est autorisée, jusqu’à une dose de 1 mg/kilo/jour.
3. Les bolus de Methylprednisolone sont autorisés.
4. Le maintien d’un traitement immunosuppresseur (methotrexate, cyclophosphamide, azathioprine, mycophenolate mofetil) ou des immunoglobulines polyvalentes est recommandé. En revanche, on tentera de limiter les traitements associés : on pourra maintenir la corticothérapie et l’un des traitements immunosuppresseurs sus cités (prescrit si possible depuis plus de 6 mois), auquel on ajoutera le rituximab, mais on évitera les associations multiples.

## Evaluation de la toxicité du Rituximab

Dans l’essai randomisé au cours de la polyarthrite rhumatoïde, peu d’effets secondaires sérieux ont été observés (une hypotension, une pneumonie, une septicémie à staphylocoque et une insuffisance rénale) (2).

L’évaluation de la tolérance sera réalisée avec les critères cliniques et biologiques habituels de surveillance :

1. Paramètres cliniques : l’examen clinique avant et après l’injection de Rituximab comprendra la mesure de la tension artérielle, la fréquence respiratoire, la saturation en oxygène au doigt et de la température, toutes les 15 minutes pendant les 2 premières heures puis toutes les 2 heures durant les 8 heures suivantes et finalement 3 fois par jour pendant les 2 jours suivant la première injection, et 8 heures pour la 2^e^ injection. Une fois par jour, un examen clinique complet viendra compléter la mesure de ces paramètres.
2. Paramètres biologiques : les paramètres biologiques usuels (NFS, natrémie, kaliémie, chlorémie, créatinémie, niveau de CPK, bilirubine, ALAT, ASAT, gamma GT, LDH, CRP) seront réalisés avant chaque injection de Rituximab.
3. Paramètres immunologiques : l’immuno-phénotypage incluant les marqueurs comme : le CD19, le CD20, le CD4, le CD25, le CD45 RO, le CD69, le CTLA-4, le CD40L, le CD8 seront réalisés au même moment.
4. La titration des auto-anticorps sera réalisée au même moment.

## Règles d’arrêt (transitoires ou définitives) du Rituximab

En cas de survenue de fièvre, de frissons, baisse de la tension artérielle ou de la saturation ou de gêne respiratoire, la perfusion sera immédiatement arrêtée et ne sera reprise qu’après accord médical. Le médecin appréciera alors le degré de gravité.

En cas d’évènement grave (au sens défini plus bas) qui peut être une hypotension sévère, une dyspnée sévère par bronchospasme, une infections virales graves (primo-infection, exacerbation (par exemple à EBV, VZV…) ou réactivation (par exemple LEMP), le rituximab devra être définitivement arrêté. Le patient reste alors dans l’étude et poursuit le suivi prévu (cf. § 4.4 Règles d’arrêt définitif ou temporaire).

En cas de réactions légères à modérées listés ci après :

| **Débit** | **Fièvre** | **Frissons** | **Congestion muqueuse Oedème** | **Chute de la PA systolique** |
| --- | --- | --- | --- | --- |
| **Diminuer le débit de 50 % en**  **cas d’apparition de l’un des symptômes** | > 38.5°C | modéré | Modéré | > 30 mm Hg |

Lorsque les symptômes présentés disparaissent, la perfusion peut reprendre mais à un débit de 50% inférieur à celui qui existait lors de l’apparition des symptômes. **La surveillance après l’incident doit reprendre toutes les 15 minutes pour la première heure puis toutes les heures.**

# 7 Statistiques

## 7.1. Plan d’analyse statistique et justification du nombre de patients à inclure :

**Myosites**

Douze patients avec une myopathie inflammatoire associée à des anti-synthétases (JO-1, PL-7 ou PL-12) et 12 patients avec une myopathie inflammatoire associée aux anti-SRP seront inclus dans l’étude.

Pour chaque groupe de patients (anti-synthétases et anti SRP), si une efficacité est observée chez au moins 6 patients, il sera possible de conclure que le taux de réponse est au-dessus de 25 % (borne inférieure à 90 % de l’intervalle de confiance pour un taux de réponse observée de 50 %).

L’efficacité est déterminée, pour un sujet donné, par une amélioration de la force musculaire définie par au moins 2 points gagnés sur l’échelle de Kendall dans au moins 2 groupes musculaires différents (soit un gain d’au moins 4 points) (18) (voir annexe 1), au 12ème mois.

**Myasthénies**

Douze patients avec une myasthénie avec anticorps anti-RAch, généralisée et réfractaire seront inclus dans l’étude.

Si un succès est observé chez au moins 6 patients, il sera possible de conclure que le taux de réponses est supérieur à 25 % (pour une borne inférieure de 90% de l’intervalle de confiance d’un taux de réponses observé de 50 %).

L’efficacité est déterminée, pour un sujet donné, par une amélioration de la force musculaire définie une augmentation d’au moins 20 points du score MMG (47, 48) au 12^ème^ mois.

## 7.2. Analyses statistiques

L’analyse statistique sera réalisée après vérification de toutes les données et comportera :

- - la description de la population des patients,
  - la description des critères d’efficacité (clinique et biologique),
  - la description des critères de tolérance (clinique et biologique),
  - la description de la réponse immune.

Les critères principaux et secondaires d'évolution des scores de testing musculaire, d'évolution de la qualité de vie, d'évolution des CPK et du titre des AAC, etc, seront évalués par la variation individuelle de la mesure par rapport à la mesure faite à J0.

Des tests statistiques seront réalisés sur les données individuelles en utilisant les pourcentages pour des variables qualitatives et les médianes (rang interquartile) pour les variables quantitatives.

Les analyses statistiques seront réalisées au sein de l’unité Inserm U 720 sous la responsabilité du Dr D Costagliola.

# 8. Aspects réglementaires :

## 8.1. Méthodes et calendrier prévus pour mesurer, recueillir et analyser les paramètres d'évaluation de la sécurité

## 8.1.1.Comité de pilotage

Il sera constitué des initiateurs cliniciens du projet, du biostatisticien en charge du projet, des représentants du promoteur et de l’URC nommés pour cette recherche.

Il définira l'organisation générale et le déroulement de la recherche et coordonnera les informations.

Il déterminera initialement la méthodologie et décidera en cours de recherche des conduites à tenir dans les cas imprévus, surveillera le déroulement de la recherche en particulier sur le plan de la tolérance et des évènements indésirables.

## 8.1.2. Comité de surveillance indépendant

Un comité de surveillance indépendant de l’étude sera mis en place.

Sa composition sera définie au début de l’étude ; il sera constitué de personnes extérieures à la recherche dont au moins un clinicien spécialiste de la pathologie étudiée et un pharmacologue/pharmacovigilant.

Ce comité a une fonction consultative et décisionnelle lorsque le promoteur fait appel à lui sur des points médicaux tels la tolérance et les événements indésirables.

Ce comité de surveillance se réunira afin d’apprécier les effets secondaires rencontrés chez les 4 premiers patients bénéficiant de l’association azathioprine et rituximab après 6 mois de traitement, ainsi qu’après les 4 premiers patients recevant l’association mycophenolate mofétil et rituximab.

L’étude pourra être interrompue à tout moment, sur la décision du comité de surveillance, en particulier devant la survenue à une plus grande fréquence que prévue d’effets indésirables graves (voir plus bas) après les injections du rituximab.

## 8.2. Description des paramètres d’évaluation de la sécurité

#### Evènement indésirable

Toute manifestation nocive survenant chez une personne qui se prête à une recherche biomédicale que cette manifestation soit liée ou non à la recherche ou au produit sur lequel porte cette recherche.

#### Effet indésirable d’un médicament expérimental

Toute réaction nocive et non désirée à un médicament expérimental quelle que soit la dose administrée

#### Evènement ou effet indésirable grave

Tout évènement ou effet indésirable qui entraîne la mort, met en danger la vie de la personne qui se prête à la recherche, nécessite une hospitalisation ou la prolongation de l’hospitalisation, provoque une incapacité ou un handicap importants ou durables, ou bien se traduit par une anomalie ou une malformation congénitale, et s’agissant du médicament, quelle que soit la dose administrée.

#### Effet indésirable inattendu d’un médicament expérimental

Tout effet indésirable dont la nature, la sévérité ou l’évolution ne concorde pas avec les informations figurant dans le résumé des caractéristiques du produit lorsque le médicament est autorisé, et dans la brochure pour l’investigateur lorsqu’il n’est pas autorisé.

#### Fait nouveau

Toute nouvelle donnée de sécurité, pouvant conduire à une réévaluation du rapport des bénéfices et des risques de la recherche ou du médicament expérimental, ou qui pourrait être suffisant pour envisager des modifications dans l’administration du médicament expérimental, dans la conduite de la recherche.

## 8.3. Procédures mises en place en vue de l'enregistrement et de la notification des évènements indésirables

## 8.3.1 Evènements indésirables non graves :

Tout événement indésirable - non grave suivant la définition précédente - observé lors de la recherche et dans ses suites devra être reporté dans le cahier d’observation dans la section prévue à cet effet.

Un seul évènement doit être reporté par item. L’évènement peut correspondre à un symptôme, un diagnostic ou à un résultat d’examen complémentaire jugé significatif. Tous les éléments cliniques ou para-cliniques permettant de décrire au mieux l’évènement correspondant doivent être reportés.

## 8.3.2 Evènements indésirables graves (EIG) :

Les investigateurs doivent notifier **immédiatement** au promoteur AP-HP les évènements indésirables graves tels que définis ci-dessus.

**L'investigateur complète les formulaires d’évènements indésirables graves (du cahier d'observation de la recherche) et les envoie au DRCD par fax au 01 44 84 17 99 et ce, dans les 48 heures** (après si possible un appel téléphonique immédiat au 01 44 84 17 23 en cas de décès ou d’une menace vitale).

L’investigateur doit également informer l’URC en charge de la recherche de la survenue de l’EIG.

Pour chaque évènement indésirable grave, **l’investigateur devra émettre un avis sur le lien de causalité de l’évènement avec chaque médicament expérimental et les autres traitements éventuels.**

L’obtention d’informations relatives à la description et l’évaluation d’un évènement indésirable peuvent ne pas être possibles dans le temps imparti pour la déclaration initiale.

**Aussi, l'évolution clinique ainsi que les résultats des éventuels bilans cliniques et des examens diagnostiques et/ou de laboratoire, ou toute autre information permettant une analyse adéquate du lien de causalité seront rapportés :**

- **soit sur la déclaration initiale d’EIG s’ils sont immédiatement disponibles,**
- **soit ultérieurement et le plus rapidement possible, en envoyant par fax une nouvelle déclaration d’EIG complétée (et en précisant qu’il s’agit d’un suivi d’EIG déclaré et le numéro de suivi).**

Toutes les déclarations faites par les investigateurs devront identifier chaque sujet participant à la recherche **par un numéro de code unique** attribué à chacun d’entre eux.

**En cas de décès notifié** d’un sujet participant à la recherche, **l’investigateur communiquera au promoteur tous les renseignements complémentaires demandés** (compte-rendu d’hospitalisation, résultats d’autopsie…).

Tout fait nouveau survenu dans la recherche ou dans le contexte de la recherche, provenant de données de la littérature ou de recherches en cours, devra être notifié au promoteur.

#### *- Déclaration des évènements indésirables graves aux Autorités de Santé*

Elle sera assurée par le Pôle de Pharmacovigilance du DRCD, après évaluation de la gravité de l’évènement indésirable, du lien de causalité avec chaque médicament expérimental et les autres traitements éventuels ainsi que du caractère inattendu des effets indésirables.

Toutes les suspicions d’effet indésirable grave inattendu seront déclarées par le promoteur aux autorités compétentes dans les délais légaux.

**Toute donnée de sécurité ou tout fait nouveau qui pourrait modifier significativement l’évaluation du rapport des bénéfices et des risques d’un médicament expérimental, ou de la recherche, ou qui pourrait conduire à envisager des modifications concernant l’administration du médicament ou la conduite de la recherche, sera transmise par le promoteur aux autorités compétentes, au Comité de Protection des Personnes et aux investigateurs de la recherche.** Par exemple :

a) toute augmentation cliniquement significative de la fréquence d’apparition d’un effet indésirable grave attendu ;

b) des suspicions d’effet indésirable grave inattendu survenus chez des participants ayant terminé l’essai et qui sont notifiés par l’investigateur au promoteur, ainsi que des rapports de suivi éventuels ;

c) tout fait nouveau concernant le déroulement de l’essai clinique ou le développement du médicament, lorsque ce fait nouveau est susceptible de porter atteinte à la sécurité des participants. A titre d’exemple :

- un événement indésirable grave susceptible d’être lié aux investigations et aux procédures de diagnostic de l’essai et qui pourrait modifier le déroulement de cet essai,
- un risque significatif pour la population de l’essai comme par exemple un manque d’efficacité du médicament utilisé dans le traitement d’une maladie mettant en jeu le pronostic vital,
- des résultats significatifs de sécurité issus d’une étude menée chez l’animal récemment terminée (telle qu’une étude de carcinogénicité),
- un arrêt anticipé ou une interruption temporaire pour des raisons de sécurité d’un essai conduit avec le même médicament dans un autre pays,
- un effet indésirable grave inattendu lié à un médicament non expérimental nécessaire à la réalisation de l’essai (ex : « challenge agents », traitement de secours)

d) les recommandations du comité de surveillance indépendant, le cas échéant, si elles sont pertinentes pour la sécurité des personnes,

e) tout effet indésirable grave inattendu transmis au promoteur par un autre promoteur d’un essai clinique mené dans un pays tiers portant sur le même médicament.

## 8.4. Modalités et durée du suivi des personnes suite à la survenue d'évènements indésirables

Tout patient présentant un évènement indésirable doit être suivi jusqu’à la résolution ou la stabilisation de celui-ci.

- Si l’évènement n’est pas grave, l’évolution en sera notée sur la page correspondante du cahier d’observation à la section prévue à cet effet.
- Si l’évènement est grave, un suivi d’EIG sera envoyé au DRCD.

# 9 DROIT D’ACCES AUX DONNEES ET DOCUMENTS SOURCE

Les personnes ayant un accès direct conformément aux dispositions législatives et réglementaires en vigueur, notamment les articles L.1121-3 et R.5121-13 du code de la santé publique (par exemple, les investigateurs, les personnes chargées du contrôle de qualité, les moniteurs, les assistants de recherche clinique, les auditeurs et toutes personnes appelées à collaborer aux essais) prennent toutes les précautions nécessaires en vue d'assurer la confidentialité des informations relatives aux médicaments expérimentaux, aux essais, aux personnes qui s'y prêtent et notamment en ce qui concerne leur identité ainsi qu’aux résultats obtenus. Les données collectées par ces personnes au cours des contrôles de qualité ou des audits sont alors rendues anonymes.

# 10 CONTROLE ET ASSURANCE DE LA QUALITE

**La recherche sera encadrée selon les procédures opératoires standard du promoteur.**

Le déroulement de la recherche dans les centres investigateurs et la prise en charge des sujets sera faite conformément à la déclaration d’Helsinki et les Bonnes Pratiques en vigueur.

## 10.1 Procédures de monitoring

Cette recherche est classée risque D selon les procédures du promoteur.

Les ARC représentants du promoteur effectueront des visites des centres investigateurs au rythme correspondant au schéma de suivi des patients dans le protocole, aux inclusions dans les différents centres et au niveau de risque qui a été attribué à la recherche.

- Visite d‘ouverture de chaque centre : avant inclusion, pour une mise en place du protocole et prise de connaissance avec les différents intervenants de la recherche biomédicale.

- Lors des visites suivantes, les cahiers d'observation seront revus au fur et à mesure de l'état d'avancement de la recherche par les ARC. L'investigateur principal de chaque centre ainsi que les autres investigateurs qui incluent ou assurent le suivi des personnes participant à la recherche s’engagent à recevoir les ARC à intervalles réguliers.

Lors de ces visites sur site et en accord avec les Bonnes Pratiques Cliniques, les éléments suivants seront revus :

- Respect du protocole et des procédures définies pour la recherche,
- Vérification des consentements éclairés des patients
- Examen des documents source et confrontation avec les données reportées dans le cahier d’observation quant à l’exactitude, les données manquantes, la cohérence des données selon les règles édictées par les procédures du DRCD.

- Visite de fermeture : récupération des cahiers d’observation, bilan à la pharmacie, documents de la recherche biomédicale, archivage.

## 10.2 Transcription des données dans le cahier d’observation

Toutes les informations requises par le protocole doivent être fournies dans le cahier d’observation et une explication donnée par l’investigateur pour chaque donnée manquante.

Les données devront être transférées dans les cahiers d'observation au fur et à mesure qu'elles sont obtenues qu'il s'agisse de données cliniques ou para-cliniques. Les données devront être copiées de façon nette et lisible à l'encre noire dans ces cahiers (ceci afin de faciliter la duplication et la saisie informatique).

Les données erronées dépistées sur les cahiers d'observation seront clairement barrées et les nouvelles données seront copiées sur le cahier avec les initiales et la date par le membre de l'équipe de l'investigateur qui aura fait la correction.

L'anonymat des sujets sera assuré par un numéro de code et les initiales de la personne qui se prête à la recherche sur tous les documents nécessaires à la recherche, ou par effacement par les moyens appropriés des données nominatives sur les copies des documents source, destinés à la documentation de la recherche.

Les données informatisées sur un fichier seront déclarées à la CNIL selon la procédure adaptée au cas.

# 11. CONSIDERATIONS LEGALES ET ETHIQUES

Le promoteur est défini par la loi 2004-806 du 9 août 2004. Dans cette recherche, l'AP-HP est le promoteur et le Département de la Recherche Clinique et du Développement (DRCD) en assure les missions réglementaires.

*Avant de démarrer la recherche, chaque investigateur fournira au représentant du promoteur de la recherche une copie de son* ***curriculum vitæ personnel daté et signé*** *et comportant son numéro d’inscription à l’ordre des médecins.*

## 11.1 Demande d’autorisation auprès de l’Afssaps

Pour pouvoir démarrer la recherche, l’AP-HP en tant que promoteur doit soumettre un dossier de demande d’autorisation auprès de l'autorité compétente l’Afssaps. L'autorité compétente, définie à l'article L. 1123-12, se prononce au regard de la sécurité des personnes qui se prêtent à une recherche biomédicale, en considérant notamment la sécurité et la qualité des produits utilisés au cours de la recherche conformément, le cas échéant, aux référentiels en vigueur, leur condition d'utilisation et la sécurité des personnes au regard des actes pratiqués et des méthodes utilisées ainsi que les modalités prévues pour le suivi des personnes.

## 11.2 Demande d’avis au Comité de Protection des Personnes

En accord avec l'article L.1123-6 du Code de Santé Publique, le protocole de recherche doit être soumis par le promoteur à un Comité de Protection des Personnes L'avis de ce comité est notifié à l’autorité compétente par le promoteur avant le démarrage de la recherche.

## 11.3 Modifications

Le DRCD doit être informé de tout projet de modification du protocole par l’investigateur coordonnateur.

Les modifications devront être qualifiées en substantielles ou non.

Une modification substantielle est une modification susceptible, d'une manière ou d'une autre, de modifier les garanties apportées aux personnes qui se prêtent à la recherche biomédicale (modification d’un critère d’inclusion, prolongation d’une durée d’inclusion, participation de nouveaux centres,…).

Après le commencement de la recherche, toute modification substantielle de celle-ci à l’initiative du promoteur doit obtenir, préalablement à sa mise en oeuvre, un avis favorable du comité et une autorisation de l’autorité compétente. Dans ce cas, si cela est nécessaire, le comité s’assure qu’un nouveau consentement des personnes participant à la recherche est bien recueilli.

Par ailleurs, toute extension de la recherche (modification profonde du schéma thérapeutique ou des populations incluses, prolongation des traitements et ou des actes thérapeutiques non prévus initialement dans le protocole) devra être considérée comme une nouvelle recherche.

Toute modification substantielle devra faire l’objet par le promoteur après paiement d’une taxe d’une demande d’autorisation auprès de l’Afssaps et/ou d’une demande d’avis du CPP.

## 11.4 Déclaration CNIL

La loi prévoit que la déclaration du fichier informatisé des données personnelles collectées pour la recherche doit être faite avant le début effectif de la recherche.

Une méthodologie de référence spécifique au traitement de données personnelles opéré dans le cadre des recherches biomédicales définies par la loi 2004-806 du 9 août 2004 car entrant dans le champ des articles L.1121-1 et suivants du Code de Santé Publique a été établie par la CNIL en janvier 2006.

Cette méthodologie permet une procédure de déclaration simplifiée lorsque la nature des données recueillies dans la recherche est compatible avec la liste prévue par la CNIL dans son document de référence.

Lorsque le protocole bénéficie d’un contrôle qualité des données par un ARC représentant le promoteur et qu’il entre dans le champ d’application de la procédure simplifiée CNIL, le DRCD en qualité de promoteur demandera au responsable du fichier informatique de s’engager par écrit sur le respect de la méthodologie de référence MR06001 simplifiée.

## 11.5 Note d’information et Consentement éclairé

Le consentement écrit doit être recueilli auprès de toute personne se prêtant à la recherche avant la réalisation de tout acte nécessité par la recherche biomédicale.

*Les patients hospitalisés ou vus lors d’une consultation se verront expliquer et proposer le protocole ; la notice d’information leur sera remise.*

*Après un délai de réflexion, le patient pourra être éventuellement inclus, dès signature du consentement*

## 11.6 Rapport final de la recherche

Le rapport final de la recherche sera écrit en collaboration par le coordonnateur et le biostatisticien pour cette recherche. Ce rapport sera soumis à chacun des investigateurs pour avis. Une fois qu'un consensus aura été obtenu, la version finale devra être avalisée par la signature de chacun des investigateurs et adressée au promoteur dans les meilleurs délais après la fin effective de la recherche. Un rapport rédigé selon le plan de référence de l’autorité compétente doit être transmis à l’autorité compétente ainsi qu’au CPP dans un délai de un an, après la fin de la recherche, s’entendant comme la dernière visite de suivi du dernier sujet inclus. Ce délai est rapporté à 90 jours en cas d’arrêt prématuré de la recherche.

# 12. TRAITEMENT DES DONNEES ET CONSERVATION DES DOCUMENTS ET DES DONNEES RELATIVES A LA RECHERCHE

Les documents d’une recherche entrant dans le cadre de la loi sur les recherches biomédicales doivent être archivés par toutes les parties pendant une durée de 15 ans après la fin de la recherche.

(voir BPC, chapitre 8 : documents essentiels)

cet archivage indexé comporte :

- Les copies de courrier d’autorisation de l’Afssaps et de l’avis obligatoire du CPP
- Les versions successives du protocole (identifiées par le n° de version et la date de version),
- Les courriers de correspondance avec le promoteur,
- Les consentements signés des sujets sous pli cacheté (dans le cas de sujets mineurs signés par les titulaires de l’autorité parentale) avec la liste ou registre d’inclusion en correspondance,
- Le cahier d’observation complété et validé de chaque sujet inclus,
- Toutes les annexes spécifiques à l’étude,
- Le rapport final de l’étude provenant de l’analyse statistique et du contrôle qualité de l’étude (double transmis au promoteur).
- Les certificats d’audit éventuels réalisés au cours de la recherche

La base de données ayant donné lieu à l’analyse statistique doit aussi faire l’objet d’archivage par le responsable de l’analyse (support papier ou informatique).

# 13. ASSURANCE ET ENGAGEMENT SCIENTIFIQUE

## 13.1 Assurance

L'Assistance Publique- Hôpitaux de Paris est le promoteur de cette recherche. En accord avec la loi sur les recherches biomédicales, elle a pris une assurance auprès de la compagnie GERLING KonZern pour toute la durée de la recherche, garantissant sa propre responsabilité civile ainsi que celle de tout intervenant (médecin ou personnel impliqué dans la réalisation de la recherche) (loi n°2004-806, Art L.1121-10 du CSP).

l'Assistance Publique - Hôpitaux de Paris se réserve le droit d'interrompre la recherche à tout moment pour des raisons médicales ou administratives; dans cette éventualité, une notification sera fournie à l'investigateur.

## 13.2. Engagement scientifique

Chaque investigateur s'engagera à respecter les obligations de la loi et à mener la recherche selon les B.P.C., en respectant les termes de la déclaration d'Helsinki en vigueur. Pour ce faire, un exemplaire de **l’engagement scientifique (document type DRCD)** daté et signé **par chaque investigateur** de chaque service clinique d’un centre participant sera remis au représentant du promoteur.

# 14 REGLES RELATIVES A LA PUBLICATION

**L’AP-HP est propriétaire des données et aucune utilisation ou transmission à un tiers ne peut être effectuée sans son accord préalable**.

seront premiers signataires des publications, les personnes ayant réellement participé à l’élaboration du protocole et son déroulement ainsi qu’à la rédaction des résultats.

*Par précaution, un comité d’écriture devrait être constitué et l’ordre des signataires pourra être défini par avance.*

L’Assistance Publique- Hôpitaux de Paris doit être mentionnée comme étant le promoteur de la recherche biomédicale et comme soutien financier le cas échéant. les termes « Assistance Publique- Hôpitaux de Paris » doivent apparaître dans l’adresse des auteurs.

# 15. Références

1. Boye J, Elter T and Engert A. An overview of the current clinical use of the anti-CD20 monoclonal antibody rituximab. Ann Oncol 2003;14:520-35

2. Edwards JC, Szczepanski L, Szechinski J, et al. Efficacy of B-cell-targeted therapy with rituximab in patients with rheumatoid arthritis. N Engl J Med 2004;350:2572-81

3. Stasi R, Pagano A, Stipa E and Amadori S. Rituximab chimeric anti-CD20 monoclonal antibody treatment for adults with chronic idiopathic thrombocytopenic purpura. Blood 2001;98:952-7

4. Gottenberg JE, Guillevin L, Lambotte O, et al. Tolerance and short term efficacy of rituximab in 43 patients with systemic autoimmune diseases. Ann Rheum Dis 2005;64:913-20

5. Keogh KA, Wylam ME, Stone JH and Specks U. Induction of remission by B lymphocyte depletion in eleven patients with refractory antineutrophil cytoplasmic antibody-associated vasculitis. Arthritis Rheum 2005;52:262-8

6. Levine TD. Rituximab in the treatment of dermatomyositis: an open-label pilot study. Arthritis Rheum 2005;52:601-7

7. Bohan A, Peter JB, Bowman RL and Pearson CM. A computer-assisted analysis of 153 patients with polymyositis and dermatomyositis. Medicine (Baltimore) 1977;56:255-86

8. Vincent A, Palace J and Hilton-Jones D. Myasthenia gravis. Lancet 2001;357:2122-8

9. Dalakas MC, Hohlfeld R. Polymyositis and dermatomyositis. Lancet 2003;362:971-82

10. Dalakas MC. The future prospects in the classification, diagnosis and therapies of inflammatory myopathies: a view to the future from the "bench-to-bedside". J Neurol 2004;251:651-7

11. Gajdos P, Chevret S and Toyka K. Intravenous immunoglobulin for myasthenia gravis. Cochrane Database Syst Rev 2003:CD002277

12. Cherin P, Pelletier S, Teixeira A, et al. Results and long-term followup of intravenous immunoglobulin infusions in chronic, refractory polymyositis: an open study with thirty-five adult patients. Arthritis Rheum 2002;46:467-74

13. Romero NB, Braun S, Benveniste O, et al. Phase I study of dystrophin plasmid-based gene therapy in Duchenne/Becker muscular dystrophy. Hum Gene Ther 2004;15:1065-76

14. Klatzmann D, Cherin P, Bensimon G, et al. A phase I/II dose-escalation study of herpes simplex virus type 1 thymidine kinase "suicide" gene therapy for metastatic melanoma. Study Group on Gene Therapy of Metastatic Melanoma. Hum Gene Ther 1998;9:2585-94

15. Benveniste O, Cherin P, Maisonobe T, et al. Severe perturbations of the blood T cell repertoire in polymyositis, but not dermatomyositis patients. J Immunol 2001;167:3521-9

16. Benveniste O, Herson S, Salomon B, et al. Long-term persistence of clonally expanded T cells in patients with polymyositis. Ann Neurol 2004;56:867-72

17. Benveniste O, Farrugia M, Clover L and Vincent A. MuSK antibody positive myasthenia gravis serum modifies NCAM and MURF-1 expression in C2C12 cultures and mouse muscle in vivo. J NeuroImmunol 2005:in press

18. Hoogendijk JE, Amato AA, Lecky BR, et al. 119th ENMC international workshop: trial design in adult idiopathic inflammatory myopathies, with the exception of inclusion body myositis, 10-12 October 2003, Naarden, The Netherlands. Neuromuscul Disord 2004;14:337-45

19. Mathews MB, Bernstein RM. Myositis autoantibody inhibits histidyl-tRNA synthetase: a model for autoimmunity. Nature 1983;304:177-9

20. Bernstein RM, Morgan SH, Chapman J, et al. Anti-Jo-1 antibody: a marker for myositis with interstitial lung disease. Br Med J (Clin Res Ed) 1984;289:151-2

21. Miller T, Al-Lozi MT, Lopate G and Pestronk A. Myopathy with antibodies to the signal recognition particle: clinical and pathological features. J Neurol Neurosurg Psychiatry 2002;73:420-8

22. Troyanov Y, Targoff IN, Tremblay JL, Goulet JR, Raymond Y and Senecal JL. Novel classification of idiopathic inflammatory myopathies based on overlap syndrome features and autoantibodies: analysis of 100 French Canadian patients. Medicine (Baltimore) 2005;84:231-49

23. Mozaffar T, Pestronk A. Myopathy with anti-Jo-1 antibodies: pathology in perimysium and neighbouring muscle fibres. J Neurol Neurosurg Psychiatry 2000;68:472-8

24. Arlet JB, Dimitri D, Pagnoux C, Boyer O, Maisonobe T, Authier FJ, Bloch-Queyrat C, Goulvestre C, Heshmati F, Atassi M, Guillevin L, Herson S, Benveniste O, Mouthon L. Marked efficacy of a therapeutic strategy associating prednisone and plasma exchange followed by rituximab in two patients with refractory myopathy associated with antibodies to the signal recognition particle (SRP). Neuromuscul Disord. 2006 May;16(5):334-6. Epub 2006 Apr 17.

25. Targoff IN, Johnson AE and Miller FW. Antibody to signal recognition particle in polymyositis. Arthritis Rheum 1990;33:1361-70

26. Hainsworth JD, Litchy S, Shaffer DW, Lackey VL, Grimaldi M and Greco FA. Maximizing therapeutic benefit of rituximab: maintenance therapy versus re-treatment at progression in patients with indolent non-Hodgkin's lymphoma--a randomized phase II trial of the Minnie Pearl Cancer Research Network. J Clin Oncol 2005;23:1088-95

27. Osserman KE, Kornfeld P, Cohen E, et al. Studies in myasthenia gravis; review of two hundred eighty-two cases at the Mount Sinai Hospital, New York City. AMA Arch Intern Med 1958;102:72-81

28. Tindall RS, Rollins JA, Phillips JT, Greenlee R, Wells L and Belendiuk G. Preliminary results of a double-blind, randomized, placebo-controlled trial of cyclosporine in myasthenia gravis. N Engl J Med 1987;316:719-24

29. Saperstein DS, Barohn RJ. Management of myasthenia gravis. Seminar in Neurology 2004;24:41-75

30. Palace J, Newsom-Davis J, B L, et al. A randomized, double-blind trial of prednisolone alone or with azathioprine in Myasthenia Gravis. Neurology 1998;50:1778-1783

31. Meriggioli MN, Ciafaloni E, Al-Hayk KA, et al. Mycophenolate mofetil for myasthenia gravis: an analysis of efficacy, safety, and tolerability. Neurology 2003;61:1438-40

32. Tindall RS, Phillips JT, Rollins JA, Wells L and Hall K. A clinical therapeutic trial of cyclosporine in myasthenia gravis. Ann N Y Acad Sci 1993;681:539-51

33. Ciafaloni E, Nikhar NK, Massey JM and Sanders DB. Retrospective analysis of the use of cyclosporine in myasthenia gravis. Neurology 2000;55:448-450

34. Drachman DB, Jones RJ and Brodsky RA. Treatment of refractory myasthenia: "rebooting" with high-dose cyclophosphamide. Ann Neurol 2003;53:29-34

35. Ponseti JM, Azem J, Fort JM, et al. Long-term results of tacrolimus in cyclosporine- and prednisone-dependent myasthenia gravis. Neurology 2005;64:1641-1643

36. Rowin J, Meriggioli MN, Tüzün E, Leurgans S and Christadoss P. Etanercept treatment in corticosteroid-dependent myasthenia gravis. Neurology 2004;63:2390-2392

37. Newsom-Davis J, Pinching AJ, Vincent A and Wilson SG. Function of circulating antibody to acetylcholine receptor in myasthenia gravis: investigation by plasma exchange. Neurology 1978;28:266-72

38. Toyka KV, Drachman DB, Griffin DE, et al. Myasthenia gravis. Study of humoral immune mechanisms by passive transfer to mice. N Engl J Med 1977;296:125-31

39. Engel AG. Myasthenia gravis and myasthenic syndromes. Ann Neurol 1984;16:519-34

40. Patrick J, Lindstrom J. Autoimmune response to acetylcholine receptor. Science 1973;180:871-2

41. Gajra A, Vajpayee N and Grethlein SJ. Response of myasthenia gravis to rituximab in a patient with non-Hodgkin lymphoma. Am J Hematol 2004;77:196-7

42. Wylam ME, Anderson PM, Kuntz NL and Rodriguez V. Successful treatment of refractory myasthenia gravis using rituximab: a pediatric case report. J Pediatr 2003;143:674-7

43. Barohn RJ, McIntire D, Herbelin L, Wolfe GI, Nations S and Bryan WW. Reliability testing of the quantitative myasthenia gravis score. Ann N Y Acad Sci 1998;841:769-72

44. Sharshar T, Chevret S, Mazighi M, et al. Validity and reliability of two muscle strength scores commonly used as endpoints in assessing treatment of myasthenia gravis. J Neurol 2000;247:286-90

45. Jaretzki A, Barohn RJ, Ernstoff RM, et al. Myasthenia gravis. Recommendations for clinical research standards. Neurology 2000;55:16-23

46. Wolfe GI, Herbelin L, Nations SP, Foster B, Bryan WW and Barohn RJ. Myasthenia gravis activities of daily living profile. Neurology 1999;52:1487-9

47. Gajdos P, Chevret S, Clair B, Tranchant C and Chastang C. Clinical trial of plasma exchange and high dose intravenous immunoglobulin in myasthenia gravis. Ann Neurol 1997;48:789-796

48. Gajdos P, Sharshar T and Chevret S. Standards of measurements in myasthenia gravis. Ann N Y Acad Sci 2003;998:445-52
