# Supplementary material for: Efficacy of Rituximab in Refractory Inflammatory Myopathies Associated with Anti- Synthetase Auto-Antibodies: An Open-Label, Phase II Trial
Source: PLoS One. 2015 Nov 5;10(11):e0133702. doi: 10.1371/journal.pone.0133702 (PMC4634756; doi:10.1371/journal.pone.0133702)
Supplement: S2 Appendix — (DOC) [file pone.0133702.s002.doc]

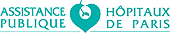


**Rituximab for treating refractory inflammatory myopathies with specific autoantibodies and generalised resistant myasthenia. "FORCE"**

1-Synopsis

[Study design 11](#__RefHeading___Toc402869984)

[2- SCIENTIFIC JUSTIFICATION AND GENERAL DESCRIPTION OF THE STUDY: 12](#__RefHeading___Toc402869985)

[2.1 Overview 12](#__RefHeading___Toc402869986)

[2.2 Information about the coordinating investigator's team and the other centres 13](#__RefHeading___Toc402869987)

[2.3 Description of the pathologies 13](#__RefHeading___Toc402869988)

[Inflammatory myopathies 13](#__RefHeading___Toc402869989)

[Resistant inflammatory myopathies associated with specific autoantibodies. 13](#__RefHeading___Toc402869990)

[Primary inflammatory myopathies: 13](#__RefHeading___Toc402869991)

[Secondary inflammatory myopathies: 15](#__RefHeading___Toc402869992)

[Results obtained: 15](#__RefHeading___Toc402869993)

[Generalised myasthenia 15](#__RefHeading___Toc402869994)

[Results obtained: 16](#__RefHeading___Toc402869995)

[3 OBJECTIVES OF THE STUDY 17](#__RefHeading___Toc402869996)

[3.1 Clinical hypotheses 17](#__RefHeading___Toc402869997)

[3.2. Objectives 17](#__RefHeading___Toc402869998)

[3.3. Benefits/Risks 18](#__RefHeading___Toc402869999)

[4 PLAN FOR THE STUDY 18](#__RefHeading___Toc402870000)

[4.1. Assessment criteria 18](#__RefHeading___Toc402870001)

[4.2. Description of the methodology 20](#__RefHeading___Toc402870002)

[4.3. Length of participation and description of the chronology of patient monitoring 20](#__RefHeading___Toc402870003)

[The expected inclusion period will be 2 3 years. The patients will participate for a period of 18 months and the total study period will be 4 5 years. Patients may not participate in any other biomedical study during the 18 months of their participation in this study. 20](#__RefHeading___Toc402870004)

[Pre-inclusion visit (W-4 to W-1) –Outpatient hospitalisation 20](#__RefHeading___Toc402870005)

[Inclusion period: Day – 1(D-1) to day + 2 (D2). 21](#__RefHeading___Toc402870006)

[Myositides and myasthenias 21](#__RefHeading___Toc402870007)

[Monitoring period: Days 7, 14, 21 and month 6.5 (+/- 8 days), 12 and 18: 21](#__RefHeading___Toc402870008)

[Study design: 22](#__RefHeading___Toc402870009)

[4.4. Rules for definitively or temporarily stopping the study 23](#__RefHeading___Toc402870010)

[5 Selection and exclusion of study subjects 23](#__RefHeading___Toc402870011)

[Myositides 23](#__RefHeading___Toc402870012)

[Inclusion criteria: 23](#__RefHeading___Toc402870013)

[- men and women aged between 18 and 70 23](#__RefHeading___Toc402870014)

[- idiopathic inflammatory myopathies defined by: 23](#__RefHeading___Toc402870015)

[- Resistant to conventional treatments 24](#__RefHeading___Toc402870016)

[Non-inclusion criteria: 24](#__RefHeading___Toc402870017)

[Inclusion criteria: 25](#__RefHeading___Toc402870018)

[- men and women aged between 18 and 70 25](#__RefHeading___Toc402870019)

[- Generalised myasthenia defined by: 25](#__RefHeading___Toc402870020)

[- Resistant to conventional treatments: 26](#__RefHeading___Toc402870021)

[Non-inclusion criteria: 26](#__RefHeading___Toc402870022)

[6 Treatment administered to study participants 27](#__RefHeading___Toc402870023)

[6.1 Description of the treatments required to conduct the research 27](#__RefHeading___Toc402870024)

[6.2. Primary adverse reactions to rituximab (Mabthera®): 27](#__RefHeading___Toc402870025)

[6.3. Treatment regimen 28](#__RefHeading___Toc402870026)

[Rituximab dose: 28](#__RefHeading___Toc402870027)

[Premedication: 28](#__RefHeading___Toc402870028)

[Concomitant treatments: depending on the context: 28](#__RefHeading___Toc402870029)

[Assessment of Rituximab's toxicity 29](#__RefHeading___Toc402870030)

[Rules for stopping Rituximab (temporarily or permanently) 29](#__RefHeading___Toc402870031)

[7 Statistics 30](#__RefHeading___Toc402870032)

[7.1. Statistical analysis plan and justification for the number of patients to include: 30](#__RefHeading___Toc402870033)

[7.2. Statistical analyses 30](#__RefHeading___Toc402870034)

[8. Regulatory aspects: 31](#__RefHeading___Toc402870035)

[8.1. Anticipated methods and timetable for measuring, collecting and analysing the parameters for assessing safety 31](#__RefHeading___Toc402870036)

[8.1.1.Steering Committee 31](#__RefHeading___Toc402870037)

[8.1.2. Independent Supervisory Committee 31](#__RefHeading___Toc402870038)

[8.2. Description of parameters for assessing safety 32](#__RefHeading___Toc402870039)

[8.3. Procedures in place for recording and reporting adverse events 32](#__RefHeading___Toc402870040)

[8.3.1 Non-serious adverse events 32](#__RefHeading___Toc402870041)

[8.3.2 Serious adverse events (SAE): 33](#__RefHeading___Toc402870042)

[8.4. Procedures for and duration of participant monitoring after adverse events have occurred 34](#__RefHeading___Toc402870043)

[9 RIGHT TO ACCESS SOURCE DATA AND DOCUMENTS 34](#__RefHeading___Toc402870044)

[10 QUALITY CONTROL AND ASSURANCE 35](#__RefHeading___Toc402870045)

[10.1 Monitoring procedures 35](#__RefHeading___Toc402870046)

[10.2 Transcription of the data in the case report form 35](#__RefHeading___Toc402870047)

[11. LEGAL AND ETHICAL CONSIDERATIONS 36](#__RefHeading___Toc402870048)

[11.1 Request for authorisation from AFSSAPS 36](#__RefHeading___Toc402870049)

[11.2 Request for an Opinion from the Comité de Protection des Personnes (ethical review board) 36](#__RefHeading___Toc402870050)

[11.3 Modifications 37](#__RefHeading___Toc402870051)

[11.4 CNIL declaration 37](#__RefHeading___Toc402870052)

[11.5 Information sheet and Informed consent 38](#__RefHeading___Toc402870053)

[11.6 Final study report 38](#__RefHeading___Toc402870054)

[12. DATA PROCESSING AND STORAGE OF RESEARCH DOCUMENTS AND DATA 38](#__RefHeading___Toc402870055)

[13. INSURANCE AND SCIENTIFIC COMMITMENT 39](#__RefHeading___Toc402870056)

[13.1 Insurance 39](#__RefHeading___Toc402870057)

[13.2. Scientific commitment 39](#__RefHeading___Toc402870058)

[14 PUBLICATION RULES 39](#__RefHeading___Toc402870059)

[15. Références 41](#__RefHeading___Toc402870060)

1- SYNOPSIS

Rituximab (B-lymphocyte depletion anti-CD20 monoclonal antibody) for treating resistant inflammatory myopathies (IMs) with specific autoantibodies and generalised resistant myasthenia. "FORCE" / P 05 12 04

**Coordinating Investigator:** Prof Olivier Benveniste, Internal Medicine Department, Hôpital Pitié Salpêtrière, Paris

Other centres: Hôpital Pitié-Salpêtrière (Internal Medicine 2, Rheumatology, Myology Institute, Neurological Life Support), Hôpital Cochin, Garches, Hôpital St Louis, Hôpital K Bicêtre, CH Lyon-Sud, Lille, Nice, Rouen, Marseille

**Treatment studied:** Rituximab 1000 mg, twice (on days 0 and 14), followed by a single injection (1000 mg) 6 months after the second injection (month 6.5 +/- 8 days).

**Primary study objective:** To assess the efficacy of rituximab in treating patients with Inflammatory Myopathies associated with specific autoantibodies (AAB) (anti-synthetase or anti-SRP), or with myasthenias (with anti-AChR), and resistant to conventional treatments.

**Selection criteria:**

Patients of both genders, aged between 18 and 70, suffering from primary IMs (defined according to the definition of the "119^th^ European Neuromuscular Centre workshop”) or from generalised myasthenias (defined according to the "Texas Clinical Classification System”) associated with the presence of specific AABs (anti-synthetase (JO1, PL7 or PL12) or anti-SRP for IMs and anti-AChR for myasthenias) and resistant to conventional treatments.

**Number of patients:**36 patients:

- 24 patients presenting a primary IM (12 with anti-synthetase AABs, 12 with anti-SRP)

- 12 with generalised myasthenia

**Study period**: The study period is 5 years: inclusion period of 3 years and monitoring or participation for 18 months (36 + 18 = 54 months). The remaining 6 months are included for statistical analysis.

**Participation period**: The participation period is 18 months.

**Method:** open, prospective, multi-centre, phase II pilot study

**Assessment criteria:**

Primary: Muscle testing (Kendall scale for IMs and score for myasthenias) at M12

Secondary: Muscle testing at D21, M6.5 and M18

Quality of life (SF 36 quality of life questionnaire)

Evolution in CPK level and in the AAB titer

Tolerance: adverse events

**Results expected:**

If the treatment is successful for at least 6 patients per group (i.e. 6 patients presenting an IM with anti-synthetases, 6 presenting an IM with anti-SRP and 6 presenting generalised myasthenia), it will be possible to conclude that the response rate is higher than 25% (lower than 90%, confidence interval for the observed response at 50%).

**Procedure for the trial:**

W-4 to W-1: diagnosis procedure, signature of the consent form, clinical exam with tests, biological analysis.

D-1: clinical exam with testing, quality of life, vital capacity for myasthenias, biological analysis with ßhCG measurement (for women)

D0: 1^st^ rituximab dose, biological analysis, clinical exam, tolerance

D1: clinical exam, tolerance

D7: biological analysis, clinical exam, tolerance

D14: 2^nd^ rituximab dose, biological analysis, clinical exam, tolerance

D21: clinical exam with testing, biological analysis, tolerance

M6.5: 3^rd^ rituximab dose, clinical exam with testing, biological analysis with ßhCG measurement (for women), tolerance

M12: clinical exam with testing, biological analysis, tolerance, quality of life, vital capacity for myasthenias

M18 clinical exam with testing, biological analysis, tolerance

The proposed ancillary studies will require patients to participate at W-4 to W-1, D-1, D21, M6.5, M12 and M18.

Study design

|  | W-4 to W-1 | D-1 | D0 | D1 | D7 | D14 | D21 | M6 | M12 | M18 |
| --- | --- | --- | --- | --- | --- | --- | --- | --- | --- | --- |
| Diagnostic procedure (with or without muscle biopsy) | ⦁ |  |  |  |  |  |  |  |  |  |
| Signature of the consent form | ⦁ |  |  |  |  |  |  |  |  |  |
| Rituximab (1000 mg) |  |  | ⦁ |  |  | ⦁ |  | ⦁ |  |  |
| Clinical exam | ⦁ | ⦁ | ⦁ | ⦁ | ⦁ | ⦁ | ⦁ | ⦁ | ⦁ | ⦁ |
| Neuro-muscular testing | ⦁ | ⦁ |  |  |  |  | ⦁ | ⦁ | ⦁ | ⦁ |
| Quality of life (SF 36) |  | ⦁ |  |  |  |  |  |  | ⦁ |  |
| VC (myasthenia) |  | ⦁ |  |  |  |  |  |  | ⦁ |  |
| Standard biological tests (CPK, etc.) | ⦁ | ⦁ |  |  | ⦁ | ⦁ | ⦁ | ⦁ | ⦁ | ⦁ |
| ßhCG (for women) |  | ⦁ |  |  |  |  |  | ⦁ |  |  |
| Intercurrent evts |  |  | ⦁ | ⦁ | ⦁ | ⦁ | ⦁ | ⦁ | ⦁ | ⦁ |
| Ancillary studies | ⦁ | ⦁ |  |  |  |  | ⦁ | ⦁ | ⦁ | ⦁ |

# SCIENTIFIC JUSTIFICATION AND GENERAL DESCRIPTION OF THE STUDY:

## 2.1 Overview

Rituximab is a chimeric monoclonal antibody specific for human CD20, which targets B lymphocytes. It was first developed (and obtained MA (market authorisation)) for the treatment of B lymphoma (1). For this indication, more than 300,000 patients have already received this drug, with very good tolerance (1).

More recently, new trials have suggested that this molecule is of interest in treating humorally mediated autoimmune diseases. The pivotal role of B cells has been shown not only for the secretion of autoantibodies or the presentation of autoantigens, but also for producing pro-inflammatory cytokines and regulating the functions of the dendritic cells (2). Rituximab could therefore be considered as a new biotherapy for autoimmune diseases, particularly for patients who are resistant to conventional treatments.

At this time, a few teams have already started using it to treat, for example, rheumatoid arthritis that is resistant to corticosteroids and conventional immunosuppressive agents (2) in a prospective, controlled, randomised study of 161 patients, or in open studies on other autoimmune diseases, such as: 25 patients with idiopathic thrombocytopenic purpura (3), 13 patients with lupus erythematosus (4), 11 patients with systemic vasculitis (5), 6 patients with primary Sjögren's syndrome (4) and 6 patients with dermatomyositis (6). Efficacy higher than 70% is observed (2, 4, 6), underlining the benefit of this biotherapy. Rituximab has also recently received AM for indications of rheumatoid arthritis.

As a reference centre for neuromuscular diseases, we recruit a large number of patients with inflammatory myopathies and generalised myasthenias. Although the physiopathology of these two diseases is different (see below), their treatment plans are very similar.

In the severe forms of these two diseases, mortality can reach 70% without an effective treatment (7, 8). The normal treatments for these two diseases are immunosuppressive agents, starting off with corticosteroids. However, more than 70% of patients treated with corticosteroids present an incomplete response. In fact, for 10% to 30% of these patients, there is no response at all (8, 9).

Treatment with corticosteroids is also associated with significant side effects, such as excess weight, glucose intolerance, cataracts, osteoporosis, myopathy caused by corticosteroids and delayed growth in children.

In patients who present an incomplete response or significant side effects from corticosteroids, the rule is to add immunosuppressive agents such as azathioprine, methotrexate, cyclophosphamide and/or cyclosporine A. The efficacy of these molecules has been shown in clinical trials with a limited number of patients, and with efficacy that is sometimes moderate (8, 10). Intravenous immunoglobulins or plasma exchanges can also offer a beneficial effect in these resistant forms of diseases, although their efficacy is short lived (11, 12).

Because some patients maintain resistance to all these treatments, and as some of them go on to develop side effects, new therapeutic approaches are necessary.

**Therefore, we are proposing an open, prospective, multi-centre, phase II pilot study to assess the efficacy of Rituximab for treating patients with inflammatory myopathy associated with specific antibodies or patients suffering from generalised myasthenia, and who are resistant to the conventional treatments.**

This first stage is necessary before a phase III trial, comparing corticosteroids to Rituximab in these indications.

## 2.2 Information about the coordinating investigator's team and the other centres

The Internal Medicine 1 Department (Professor Serge Herson) is associated with the Myology Institute at Pitié-Salpétrière (Professor Bruno Eymard) and is certified as a Reference centre for rare neuromuscular diseases.

At this centre, more than 300 patients with inflammatory myopathy and 100 with generalised myasthenia are being monitored. In addition, the Internal Medicine Department includes the gene therapy centre, featuring a P2-level confinement chamber and an adjacent P2 laboratory (managed by Professor David Klatzmann).

Biotherapy trials have already been carried out in this location (13, 14).

The coordinating investigator (Olivier Benveniste) works as a clinician in both departments. He also developed a research topic on inflammatory myopathies in David Klatzmann's laboratory at Pitié-Salpétrière (15, 16) and has carried out research on myasthenia at Oxford in the laboratory of Angela Vincent (17).

This trial represents the coordinated efforts of investigators from different departments (internal medicine, rheumatology, neurology, myology).

Over the years, these investigators have accumulated additional expertise in the understanding of physiopathology and the development of clinical trials for autoimmune diseases.

This study involves 2 different pathologies (inflammatory myopathies and generalised myasthenia). However, their treatment regimen and monitoring procedures are the same.

## 2.3 Description of the pathologies

## Inflammatory myopathies

## Resistant inflammatory myopathies associated with specific autoantibodies.

Inflammatory myopathies can be primary (idiopathic) or secondary to other connective tissue diseases. But they are both characterised by the occurrence of gradual muscle weakness (which can eventually affect the muscles used in swallowing and the respiratory muscles and thus become life threatening) and by an inflammatory infiltrate on a muscle biopsy.

### Primary inflammatory myopathies:

Primary inflammatory myopathies can be divided into 5 categories, depending on their clinical and immuno-histological aspects (7, 18): 1) dermatomyositis (DM), a microvasculitis mediated by the antibodies characterised by the deposit of C5b9 membrane attack complex in the capillaries of the dermis and the muscle, leading to ischaemic phenomena, 2) polymositis (PM), and 3) inclusion-body myositis (IBM), 2 diseases associated with a hyper-expression of class I HLA on the muscle fibres, resulting in lysis of the muscle fibres by CD8-positive, cytotoxic T-Lymphocytes, directed at an antigen that is as yet unknown, 4) non-specific myositides and 5) immune-mediated neocratising myopathies.

Up to 50% of these patients present autoantibodies. The most commonly found autoantibodies are non-specific untyped antinuclear factors or anti-RNP, anti-SSA, anti-PM/Scl, etc. However, specific autoantibodies associated with inflammatory myopathies are found in 10% to 30% of DMs or PMs and more rarely in IMs. The autoantibodies that most clearly indicate inflammatory myopathies are directed against the amino-acyl-ARNt synthetases which connect each amino-acyl to its transfer RNA during protein synthesis. These are anti-JO-1 (histidyl – ARNt) (19), PL-7 (threonyl - ARNt), PL-12 (Alanine – ARNt), OJ (isoleucil - ARNt), and EJ (glycyl – ARNt) antibodies. They are found in 10% to 30% of cases, most often in patients suffering from polymyositides with mechanic's hands, arthritis, interstitial lung disease, and a Raynaud's phenomenon that constitutes anti-synthetase syndrome (20).

This particular form of inflammatory myopathy is severe, because it is located in the lungs and because it is resistant to corticosteroid treatments.

More recently, other specific autoantibodies, directed against the signal recognition particle (SRP), have been found in 4% to 6% of patients suffering from inflammatory myopathy (21). Again, they represent a specific syndrome, combining a rapidly evolving myopathy, a high CPK level, frequent myocardial damage, a high degree of necrosis and regeneration in the muscle fibres and resistance to corticosteroid treatments (21).

In July 2005, a new classification for inflammatory myopathies was proposed (22), based on the patients' clinico-serological characteristics. The authors suggested classifying patients as having pure PM or pure DM, myositis associated with cancer, or based on overlap syndrome. Overlap syndrome is the most common within the cohort (70.5%). It is clinically defined by the presence of extra-muscular or cutaneous signs (Raynaud syndrome, arthritis, sclerodactyly, mechanic's hands, fever, interstitial lung disease, PAH, pseudo-occlusion syndrome, etc.) and according to serological criteria (presence of anti-synthetase, anti-SRP and anti-nucleoporin antibodies and/or autoantibodies associated with systemic scleroderma).

Again, the anti-synthetase or anti-SRP antibodies are markers of a chronic inflammatory myopathy which is resistant to corticosteroid treatment (22).

Inflammatory myopathies associated with specific, anti-synthetase or anti-SRP autoantibodies are therefore clinically specific in terms of severity and corticosteroid resistance.

Several types of arguments favour humoral mediation:

1. for anti-synthetase syndrome where the muscle biopsy carried out on 11 patients with anti-Jo-1 antibodies is different from that described for PMs/DMs or IMs (23).
   1. it contains areas of fragmentation and perimysial rarefaction of the connective tissue, where the inflammatory cells are predominantly located in the perimysium
   2. most of the inflammatory cells are macrophages and in rare focal isolates in the perimysium, a population containing a few lymphocytes can be found
   3. the anti-JO1 antibodies are detected in the patients' serum although their physiopathological roles are still unknown at this time (23)
2. For the "anti-SRP" syndrome:
   1. There is no argument for T CD8 cytotoxic cells playing a role here, since there is no hyper-expression on the class I HLA muscle fibres and moreover the "immunoscope" analyses have shown a normal lymphocyte repertoire in one of our patients (24), as has been reported during DM, which is a contrast to the severe disturbances in the T lymphocyte repertoire, observed during PM which is therefore a diseased mediated by TCD 8+ lymphocytes (15).
   2. C5b-9 membrane attack complex is deposited both in the endomysial capillaries and in the non-necrotic capillaries in the sarcolemma (24).
   3. The anti-SRP antibodies are detected in the patients' serum although their physiopathological role is still unknown at this time (25).

The summary of these results is thus sufficient grounds to believe that humoral mediation is involved in the physio-pathogenesis of the anti-synthetase and anti-SRP syndrome and therefore suggests that we should treat these patients with Rituximab.

### Secondary inflammatory myopathies:

Moreover, some inflammatory myopathies are secondary to other connective tissue diseases such as scleroderma, Sjögren's syndrome, lupus erythematosus, etc. In this context, myopathy is associated with the specific characteristics of the underlying connective tissue disease (including the presence of specific autoantibodies). Thus, this myopathy could also be suitable for treatment with Rituximab (4) but as this patient population is not homogenous, it is not included in this study.

## Results obtained:

Six patients suffering from DM, 2 of whom had anti-JO1 antibodies (6) and 2 others suffering from PM who also had anti-JO1 antibodies (4) were successfully treated with Rituximab and were recently reported in literature. We also reported 2 other cases of inflammatory myopathies with anti-SRP antibodies where the effect we observed from Rituximab was remarkable (24).

## Generalised myasthenia

Myasthenia is an auto-immune disease associated with anti-acetylcholine-receptor autoantibodies (anti-RAch) in 80% of patients or with anti-muscle-specific kinase antibodies (anti-Musk, 10% of patients) (8). These autoantibodies induce an interruption in neuromuscular transmission. This interruption is characterised by muscle fatigability and a decrement in the EMG after repeated stimulation. In about 20% of patients, it is combined with malignant thymoma which requires surgical ablation.

The most remarkable clinical characteristic is muscle fatigability and weakness. It affects different muscles: most commonly the oculomotor, bulbar, respiratory muscles and the girdles. Severity is variable, ranging from purely ocular, minimal forms to generalised severe forms. Myasthenia is considered generalised when extra-ocular muscles are affected. The percentage of patients with generalised forms ranges from 40% to 60% and up to 10% of patients will present, at a given time during their illness, a severe respiratory attack (called a myasthenic crisis).

Patients with generalised forms, who are untreated or treated ineffectively, are at risk of deterioration that could result in a myasthenic crisis. When such a crisis occurs, life support with a ventilator may be necessary (27). Polyvalent immunoglobulins and plasma exchanges are the treatments for these acute flare ups.

In addition to anti-cholinesterase drugs, patients suffering from generalised forms must be treated throughout with corticosteroids and immunosuppressants (azathioprine, mycophenolate mofetil and even cyclosporine (28-31)). However, between 5% and 10% of patients maintain resistance to these drugs (32-33). Other immunosuppressants can then be tried, such as cyclophosphamide (34), tacrolimus or etanercept (36) but their efficacy has never been demonstrated on the basis of randomised trials. Anti-CD20s have been recently suggested as a long-term treatment for refractory myasthenia (see below).

There are a large number of arguments in favour of the humoral nature of myasthenia.

1. The presence of autoantibodies leads to the disappearance of acetylcholine receptors which reduce the efficacy of neuromuscular transmission, causing muscle weakness and fatigability (8).
2. The transplacental transfer of these autoantibodies in women suffering from myasthenia can result in foetal and/or neonatal myasthenia in their children (8).
3. Plasma exchanges produce a remarkable effect in myasthenia patients (37), even though this improvement is not permanent. The clinical benefit is correlated to the reduction of the anti-RAch antibody titer.
4. Plasma and purified IgG from patients are capable of transferring the disease to mice (38).
5. Immunisation against the acetylcholine receptor leads to a myasthenic syndrome in animals (40).
6. Human IgGs are detected in the motor plates of patients with the disease (39).

All of these arguments make it possible to establish that myasthenia is caused by the presence of anti-RAch autoantibodies. This is sufficient grounds to prove the humoral nature of this disease and motivate us to treat these patients with Rituximab.

## Results obtained:

2 cases of refractory myasthenia were successfully treated with Rituximab (41-42).

Recently, we have also treated three myasthenia patients with this drug, with good results in the first 2 patients (poster CA170, 53rd SNFMI Conference, Nancy 14-17 June 2006), the 3^rd^ is currently being treated.

# 3 OBJECTIVES OF THE STUDY

## 3.1 Clinical hypotheses

To assess in an open, prospective, multi-centre, phase II pilot study, the efficacy of Rituximab for treating patients with inflammatory myopathy associated with specific antibodies or patients suffering from generalised myasthenia, and who are resistant to the conventional treatments and thus facing a threat to their functional prognosis or even to their life prognosis.

## 3.2. Objectives

Primary objective:

To assess the efficacy of Rituximab in treating patients with inflammatory myopathy associated with specific autoantibodies or patients suffering from generalised myasthenia, who are resistant to conventional treatments**.**

For patients with **inflammatory myopathy**, an improvement in muscle strength for a given patient, defined by at least a 2-point gain on the Kendall scale in at least 2 different muscle groups (i.e. a gain of at least 4 points) (18) (see Appendix 1), in the 12th month, is the efficacy objective used.

If the treatment is successful for at least 12 patients (6 patients with anti-synthetase AABs, 6 with anti-SRP AABs), it will be possible to conclude that the response rate is higher than 25% (lower than 90%, confidence interval for the observed response at 50%).

For patients suffering from **generalised myasthenia**, an improvement in muscle strength for a given subject, defined by an increase of at least 20 points in the MMG score (47, 48) (see Appendix 2) in the 12^th^ month, is the efficacy objective used.

If the treatment is successful for at least 6 patients, it will be possible to conclude that the response rate is higher than 25% (lower than 90%, confidence interval for the observed response at 50%).

Secondary objectives:

**Myositides**

- - An improvement in muscle strength, defined on the Kendall scale as at least a 2-point gain in 2 different muscle groups (i.e. a gain of at least 4 points) (18) at day 21, in the 6^th^ month and at M18.
  - An improvement in quality of life (SF 36 self-evaluation).
  - A decrease in CPK levels.
  - A decrease in autoantibody levels.
  - An improvement in the patients' extra-muscular activity, such as lung damage on pulmonary function tests.
  - A reduction in the doses or in the number of related immunomodulatory treatments.

**Generalised myasthenia**

- An improvement in muscle strength, defined by an increase of at least 20 points in the MMG score (47, 48) at day 21, in month 6 and in M18.

- An improvement in MGFA postintervention status (45).

- An improvement in the ADLs score

- - An improvement in quality of life (SF 36 self-evaluation).

- A reduction in the number of clinical exacerbations that require either hospitalisation or a change in corticosteroid doses.

- A reduction in corticosteroid doses and related treatments

- A decrease in autoantibody levels.

- An increase in vital capacity.

## 3.3. Benefits/Risks

The lives of the patients who must be included in the study are at risk due to 1) the severity of their diseases (myositis or myasthenia) which affect the oropharyngeal and respiratory muscles, and 2) resistance to the various immunomodulators tested. Rituximab - the efficacy of which will be tested here - is used as a salvage therapy. In our experience, the efficacy of rituximab is only evident after 8 to 16 weeks. Given these seriously ill patients and to cover rituximab's "silent" phase, it would not be ethical to stop any concomitant immunomodulatory treatment. This is why, at the investigator's discretion, associated immunomodulatory treatments are permitted and indeed recommended. Thus, maintaining corticosteroids in combination with methotrexate, cyclophosphamide, azathioprine, mycophenolate mofetil or polyvalent immunoglobulins is recommended. However, we will try to limit combined treatments: corticosteroid treatment and one of the immunosuppressants listed above can be maintained (prescribed for longer than 6 months, if possible), to which rituximab will be added, but multiple combinations will be avoided. The possibility of severe adverse reactions has been taken into consideration by the investigators, in particular after discussions with the Roche laboratory, which will provide the rituximab. The risk/benefit balance leans in favour of these combined treatments. Nonetheless, an **independent** **supervisory committee** (see § 8 regulatory aspects) tasked with monitoring all adverse reactions will meet after the inclusion of the first 4 patients. This committee will make the decision to proceed with the trial. One of the primary benefits expected if rituximab is effective, in addition to a clinical improvement for patients, will be to be able to gradually lighten the burden of combined treatments in patients who were initially at a therapeutic impasse.

# 4 PLAN FOR THE STUDY

## 4.1. Assessment criteria

**Myositides**

#### Primary criterion:

The evolution in muscle strength at 12 months, assessed by scores on the Kendall scale for different muscle groups (18) (see Appendix 1), is the primary criterion: for a given subject, this is the difference between the scores in each muscle group on the Kendall scale at M12 and the scores at D0.

#### Secondary criteria:

- - muscle strength defined on the Kendall scale (see Appendix 1) at D21, M6.5 (+/- 8 days) and M18
  - quality of life (SF 36 self-evaluation) (see Appendix 3)
  - CPK levels
  - autoantibody levels
  - extra-muscular activity for the disease, such as lung damage on pulmonary function tests.
  - doses or quantities of related immunomodulatory treatments.

Tolerance assessment criteria:

- - recording intercurrent events
  - blood pressure and heart rate readings
  - blood count, liver enzymes, etc. for the biological monitoring of the treatment

**Generalised myasthenia**

#### Primary criterion:

Evolution n muscle strength at 12 months using the MMG score (47, 48) (see Appendix 2), is the primary criterion: for a given subject, this is the difference between the subject's MMG score at M12 and his/her score at D0.

#### Secondary criteria:

- muscle strength defined by MMG score (47, 48) on day 21, at month 6 and at M18.

- MGFA postintervention status (45).

- ADLs score

- - quality of life (SF 36 self-evaluation) (see Appendix 3)

- number of clinical exacerbations that require either hospitalisation or a change in corticosteroid doses.

- corticosteroid doses and related treatments

- autoantibody levels

- vital capacity

Tolerance assessment criteria:

- - recording intercurrent events
  - blood pressure and heart rate readings
  - blood count, liver enzymes, etc. for the biological monitoring of the treatment

## 4.2. Description of the methodology

This is an open, perspective, multi-centre, phase II pilot study.

A total of 36 patients will be included, broken down as follows: 24 patients presenting myositis (12 suffering from myositis with anti-synthetase and 12 suffering from myositis with anti-SRP), and 12 patients suffering from generalised myasthenia.

## 4.3. Length of participation and description of the chronology of patient monitoring

## The expected inclusion period will be 2 3 years. The patients will participate for a period of 18 months and the total study period will be 4 5 years. Patients may not participate in any other biomedical study during the 18 months of their participation in this study.

### Pre-inclusion visit (W-4 to W-1) –Outpatient hospitalisation

Diagnosis procedure: patients with potential inflammatory myopathy associated with specific autoantibodies our generalised myasthenia will have been informed about the study in advance. After signed the informed consent, the patients will be pre-included and will be subject to the follow diagnosis procedure in order to confirm or reverse their inclusion:

**Myositides**

1. Clinical exam with testing according to the Kendall scale (18)
2. Echocardiography
3. EMG
4. MRI of the muscles
5. Chest scan and pulmonary function tests.
6. Full body scan and, depending on the clinical context, additional endoscopic exams.
7. Usual biological exams (CBC, serum electrolytes, full hepatic assessment, CRP, CPK) and immunoglobulin quantitation (IgG and IgM).
8. Blood tests for HIV, HBV, HCV, HTLV1
9. Muscle biopsy, if not done previously
10. Biological exams specific to the ancillary studies

**Generalised myasthenia**

1. Clinical exam with MMG and MGFA testing (44, 45)
2. Echocardiography
3. EMG
4. Pulmonary function tests
5. Chest scan (screening for a thymoma)
6. Usual biological exams (CBC, serum electrolytes, full hepatic assessment, CRP, CPK) and immunoglobulin quantitation (IgG and IgM).
7. Blood tests for HIV, HBV, HCV, HTLV1
8. Biological exams specific to the ancillary studies

### Inclusion period: Day – 1(D-1) to day + 2 (D2).

### Myositides and myasthenias

**Day – 1 (D-1)**:

Clinical exam with muscle testing and standard biological exams (CBC, serum electrolytes, full hepatic assessment (gamma GT, alkaline phosphatase, ASAT/ALAT, Bilirubin), CPK, ßhCG for women) specific to ancillary studies, quality of life, vital capacity for myasthenia.

**Day 0 (DO) to Day 2 (D2)**:

Infusion of the first rituximab dose (1000 mg) after related preventive measures have been completed, see details in Appendix 2.

After the first injection of rituximab: clinical exams including blood pressure, respiratory rate, oxygen saturation (finger), temperature every 15 minutes for the first 2 hours, after the infusion has started, then every 2 hours for the next 8 hours and finally 3 times a day until D2. (See the nurse's sheet).

Recording of intercurrent events and related medications

### Monitoring period: Days 7, 14, 21 and month 6.5 (+/- 8 days), 12 and 18:

**D7:**

Clinical exam and standard biological exams (CBC, serum electrolytes, full hepatic assessment, CPK)

Recording of intercurrent events and related medications.

**D14:**

Clinical exam and standard biological exams (CBC, serum electrolytes, full hepatic assessment, CPK) prior to infusion.

Infusion of the second rituximab dose (1000 mg) after related preventive measures have been completed.

After the injection of rituximab: clinical exams including blood pressure, respiratory rate, oxygen saturation (finger), temperature every 15 minutes for the first 2 hours, after the infusion has started, then every 2 hours for the next 8 hours (see the nurse's sheet).

Recording of intercurrent events and related medications

**D21**:

Clinical exam with neuro-muscular testing and standard biological exams (CBC, serum electrolytes, full hepatic assessment, CPK) specific to ancillary studies.

Recording of intercurrent events and related medications

**M 6.5 (+/-8 days)**:

Clinical exam with neuro-muscular testing (before the infusion), ßhCG levels, standard biological exams (CBC, serum electrolytes, full hepatic assessment, CPK) specific to ancillary studies.

Infusion of a maintenance dose of rituximab (1000 mg)

After the injection of rituximab: clinical exams including blood pressure, respiratory rate, oxygen saturation (finger), temperature every 15 minutes for the first 2 hours, after the infusion has started, then every 2 hours for the next 8 hours finally 3 times a day for 48 hours (see the nurse's sheet).

Recording of intercurrent events and related medications

**M12 (+/-8 days)**:

Clinical exam with neuro-muscular testing and standard biological exams (CBC, serum electrolytes, full hepatic assessment, CPK) specific to ancillary studies.

Neuromuscular assessment: EMG and muscle MRI (Myositis only)

Pulmonary function tests

Quality of life

Recording of intercurrent events and related medications

**M18 (+/-8 days): end of monitoring**

Final clinical exam with neuro-muscular testing and standard biological exams (CBC, serum electrolytes, full hepatic assessment, CPK) specific to ancillary studies.

Recording of intercurrent events and related medications

### Study design:

|  | W-4 to W-1 | D-1 | D0 | D1 | D7 | D14 | D21 | M6 | M12 | M18 |
| --- | --- | --- | --- | --- | --- | --- | --- | --- | --- | --- |
| Diagnostic procedure (with or without muscle biopsy) | ⦁ |  |  |  |  |  |  |  |  |  |
| Signature of the consent form | ⦁ |  |  |  |  |  |  |  |  |  |
| Rituximab (1000 mg) |  |  | ⦁ |  |  | ⦁ |  | ⦁ |  |  |
| Clinical exam | ⦁ | ⦁ | ⦁ | ⦁ | ⦁ | ⦁ | ⦁ | ⦁ | ⦁ | ⦁ |
| Neuro-muscular testing | ⦁ | ⦁ |  |  |  |  | ⦁ | ⦁ | ⦁ | ⦁ |
| Quality of life (SF 36) |  | ⦁ |  |  |  |  |  |  | ⦁ |  |
| VC (myasthenia) |  | ⦁ |  |  |  |  |  |  | ⦁ |  |
| Standard biological tests (CPK, etc.) | ⦁ | ⦁ |  |  | ⦁ | ⦁ | ⦁ | ⦁ | ⦁ | ⦁ |
| ßhCG (for women) |  | ⦁ |  |  |  |  |  | ⦁ |  |  |
| Intercurrent evts |  |  | ⦁ | ⦁ | ⦁ | ⦁ | ⦁ | ⦁ | ⦁ | ⦁ |
| Ancillary studies | ⦁ | ⦁ |  |  |  |  | ⦁ | ⦁ | ⦁ | ⦁ |

## 4.4. Rules for definitively or temporarily stopping the study

***Withdrawal from the trial***

A patient may leave the study at his or her own will (withdrawal of the patient's consent).

Whenever possible, a biological and clinical assessment at the time of the patient's withdrawal (within the month following the withdrawal of consent) will be carried out. Any subjects who leave the trial will not be replaced.

In all cases, the reason for leaving the trial will be noted in the case report form.

***Discontinuing treatment***

Treatment will be discontinued for the following reasons:

- tolerance problem, e.g. if continuing treatment is s threat to

the patient’s safety

- unpleasant side effects

- pregnancy before the 1^st^ injection or during the study or any other reason that makes it impossible to continue

In all cases, the reason for discontinuing treatment will be noted in the case report form.

In addition, even if the patient no longer receives the treatment, he or she will not be considered as having left the trial. The patient will be assessed after the 18 months.

For a patient who is ***lost-to-follow-up***, every effort will be made to obtain updates about the patient (through the general practitioner or the family if necessary), and if possible to schedule a consultation.

# 5 Selection and exclusion of study subjects

## Myositides

## Inclusion criteria:

### - men and women aged between 18 and 70

- for women of reproductive age, use of effective contraception is required for the entire duration of their participation in the trial and for one year after the last rituximab infusion.

### - idiopathic inflammatory myopathies defined by:

1 – Myositis as defined by the 119^th^ ENMC (18):

a) Proximal myopathy with muscle weakness.

Points a, b and c must be present.

b) Subacute onset or more slowly progressive in patients older than 18.

c) Muscle necrosis and regeneration and/or presence of inflammatory infiltrates in the muscle biopsy.

d) Myogenic syndrome on the EMG (optional criteria)

2 – Presence of specific autoantibodies: anti-synthetase (anti-JO-1, anti-PL-7 or anti-PL-12) or anti-SRP.

### - Resistant to conventional treatments

Resistance to conventional treatments is defined by an inadequate response or intolerable side effects with conventional treatments.

These treatments primarily include corticosteroids which must have been used continuously for longer than 6 months. But also, other immunosuppressants for which the prescription must not have been modified (except for adjustments to the doses) within the 3 months preceding inclusion. These are azathioprine, methotrexate, mycophenolate mofetil, cyclophosphamide, cyclosporine, polyvalent immunoglobulins and/or plasma exchanges. Thus, corticosteroids and at least two of these drugs or these therapeutic approaches (used sequentially or in combination) must have been tested successfully before inclusion, in accordance with the time frames indicated above. An inadequate response is defined as the absence of an improvement and/or a deterioration in the assessed parameters (muscle strength, vital capacity, increase in CPKs, etc.), in spite of these conventional treatments, causing the prescriber to modify or to reintroduce other treatments.

## Non-inclusion criteria:

1. Other muscle pathologies such as:
   1. Inclusion-body myositis.
   2. Macrophagic myofasciitis.
   3. Hereditary myopathies.
2. Inflammatory myopathies which are secondary to other connective tissue disorders:
   1. Systemic scleroderma (according to ARA criteria and/or LEROY and MEDSGER criteria).
   2. Sjögren's syndrome (European criteria).
   3. Systemic lupus erythematosus (ACR criteria).
   4. Rheumatoid arthritis (ACR criteria).
   5. Mixed connective tissue disease.
3. Cancers or myositides associated with cancer (22)
4. Under 18 years old or over 70 years old
5. Failure to sign the informed consent form
6. Patient not affiliated with the social security regime
7. History of severe allergic reaction or anaphylactic reaction to a murine monoclonal or humanised antibody, or known hypersensitivity to one of the components of rituximab.
8. History of severe allergic reaction or anaphylactic reaction to methylprednisolone.
9. Severe heart or pulmonary condition (in the opinion of the investigator: to be documented in the CRF).
10. Presence of an uncontrolled concomitant disease, such as a condition affecting the central nervous system, kidneys, or liver, or an endocrine or gastro-intestinal condition, which in the investigator's opinion does not allow the patient to be included.
11. Active infection of any kind. History of recurrent or chronic severe infection. History of deep infection related to fasciitis, abscess, osteomyelitis during the previous 52 weeks.
12. Immunodeficiency syndrome, including seropositivity for HIV.
13. Surgical operation scheduled in the year following inclusion.
14. Any type of vaccination during the previous 28 days (it is advisable to seriously consider the indication of a vaccination (e.g. a flu vaccine for a patient suffering from a neuromuscular condition) prior to treatment with rituximab and to carefully assess the efficacy (which may be completely lost simply due to the action mechanism of rituximab). In other words, the need to vaccinate patients must be anticipated well in advance of 28 days prior to the rituximab injection. The use of a live attenuated vaccine is prohibited for the entire duration of the study (and for the 28 days prior to the study).
15. Treatment with any experimental agent within 28 days of the infusion or at 5 half-lives of the drug in question (the longest of these two time frames is to be taken into account).
16. Previous treatment (within one month of inclusion) with a cell depletion therapy, of any kind whatsoever, such as, for example, CAMPATH, anti CD4, anti CD5, anti CD3, anti CD19, anti CD11a, anti CD22, anti-Blys/BA.
17. Positive serum ßhCG result at the screening or any other positive pregnancy test carried out before the first rituximab injection.
18. Pregnancy or breast feeding or attempting to become pregnant during the study.

19. Positive **hepatitis B** test (positive Hbs antigen and/or positive PCR HBV).

However, patients presenting previous hepatitis that has been cured (AgHBS negative, Ac anti-Hbc +/-, Ac anti-Hbs+) can be included with specific monitoring for Ag  Hbs and PCR HBV every three months and 6 months after the end of the Study or **hepatitis C**.

20. Haemoglobin < 8.0 g/dL

21. Polynuclear neutrophil <1.5 X 10 ³/µL.

22. IgG and/or IgM levels < 5.0 and 0.40 mg /mL, respectively.

**Generalised myasthenia**

## Inclusion criteria:

### - men and women aged between 18 and 70

- for women of reproductive age, use of effective contraception is required for the entire duration of their participation in the trial and for one year after the last rituximab infusion.

### - Generalised myasthenia defined by:

Seropositive generalised myasthenia as defined by the TEXAS CLINICAL CLASSIFICATION SYSTEM (43), at a clinical stage > class III (IVa, IVb or V), i.e., with sever weakness affecting the limbs or the axial muscles (class IVa), or bulbar and respiratory damage (class IVb), or requiring mechanical ventilation (class V).

1. Severe weakness (class IVa, IVb or V) in the extra-ocular muscles quantified in the myasthenic score (MMS) and for which inter-and intra-observer reproducibility has been shown (44).
2. Presence of specific autoantibodies: anti-RAch.

### - Resistant to conventional treatments:

Resistance to conventional treatments is defined by an inadequate response or intolerable side effects with conventional treatments.

These treatments primarily include corticosteroids which must have been used continuously for more than one year. But also, other immunosuppressants for which the prescription must not have been modified (except for adjustments to the doses) within the 6 months preceding inclusion. These are azathioprine, mycophenolate mofetil, methotrexate, cyclophosphamide, cyclosporine, polyvalent immunoglobulins and/or plasma exchanges. Thus, corticosteroids and at least two of these drugs or these therapeutic approaches (used in combination) must have been tested successfully before inclusion, in accordance with the time frames indicated above. An inadequate response is defined as the absence of an improvement and/or a deterioration in the assessed parameters (muscle strength, vital capacity, etc.), in spite of these conventional treatments, causing the prescriber to modify or to reintroduce other treatments.

## Non-inclusion criteria:

- 1. Other myasthenic syndromes, such as:
     - - Non-generalised myasthenias (ocular).
       - Lambert-Eaton syndrome.
       - Myasthenias associated with a malignant thymoma.
       - Hereditary myasthenic syndrome.
  2. Under 18 years old or over 70 years old
  3. Failure to sign the informed consent form
  4. Patient not affiliated with the social security regime
  5. History of severe allergic reaction or anaphylactic reaction to a murine monoclonal or humanised antibody, or known hypersensitivity to one of the components of rituximab.
  6. History of severe allergic reaction or anaphylactic reaction to methylprednisolone.
  7. Severe heart or pulmonary condition (in the opinion of the investigator: to be documented in the CRF).
  8. Presence of an uncontrolled concomitant disease, such as a condition affecting the central nervous system, kidneys, or liver, or an endocrine or gastro-intestinal condition, which in the investigator's opinion does not allow the patient to be included.
  9. Active infection of any kind. History of recurrent or chronic severe infection. History of deep infection related to fasciitis, abscess, osteomyelitis during the previous 52 weeks.
  10. Immunodeficiency syndrome, including seropositivity for HIV.
  11. Surgical operation scheduled in the year following possible inclusion.
  12. Any type of vaccination during the previous 28 days (it is advisable to seriously consider the indication of a vaccination (e.g. a flu vaccine for a patient suffering from a neuromuscular condition) prior to treatment with rituximab and to carefully assess the efficacy (which may be completely lost simply due to the action mechanism of rituximab). In other words, the need to vaccinate patients must be anticipated well in advance of 28 days prior to the rituximab injection. The use of a live attenuated vaccine is prohibited for the entire duration of the study (and for the 28 days prior to the study).
  13. Treatment with any experimental agent within 28 days of the infusion or at 5 half-lives of the drug in question (the longest of these two time frames is to be taken into account).
  14. Previous treatment (within one month of inclusion) with a cell depletion therapy, of any kind whatsoever, such as, for example, CAMPATH, anti CD4, anti CD5, anti CD3, anti CD19, anti CD11a, anti CD22, anti-Blys/BA.
  15. Positive serum beta hCG result at the screening or any other positive pregnancy test carried out before the first rituximab injection.
  16. Pregnancy or breast feeding or attempting to become pregnant during the study.

17.Positive **hepatitis B** test (positive Hbs antigen and/or positive PCR HBV).

However, patients presenting previous hepatitis that has been cured (AgHBS negative, Ac anti-Hbc +/-, Ac anti-Hbs+) can be included with specific monitoring for Ag  Hbs and PCR HBV every three months and 6 months after the end of the Study or **hepatitis C**.

18.Haemoglobin < 8.0 g/dL

19.Polynuclear neutrophil <1.5 X 10 ³/µL.

20.IgG and/or IgM levels < 5.0 and 0.40 mg /mL, respectively.

## 6 Treatment administered to study participants

## 6.1 Description of the treatments required to conduct the research

Rituximab chimeric monoclonal murine/human antibody obtained through genetic engineering. It is a glycosylated immunoglobulin combining the constant regions of a human IgG1as well as the heavy and light chain variable regions of murine origin.

A single-use bottle contains 500 mg/50 ml of rituximab.

Each ml of solution contains 10 mg of rituximab.

The prepared MabThera solution must be administered via intravenous infusion using a specific route. The diluted solution for IV infusion must not be injected rapidly or administered as a bolus.

## 6.2. Primary adverse reactions to rituximab (Mabthera®):

Intravenous administration of (Mabthera®), can cause a certain number of adverse reactions. One of the side effects, that occurs particularly during the first two hours following the first infusion, is characterised by fever, chills and tremors, sometimes combined with an outbreak of uticaria or even a drop in blood pressure and in the worst case difficulty breathing. Premedication (see below) intended to prevent these manifestations will be systematically administered when the medication is injected. Moreover, in order to minimise these reactions, the rituximab injection speeds will be gradually increased as it is administered (see Appendix 16.6, "Procedure for the intravenous administration of Mabthera").

Other possible adverse reactions are: pain (abdomen, chest, muscles, joints, injection sites), sensations of discomfort, changes in blood pressure.

Like immunosuppressants and corticosteroids, rituximab (Mabthera®) also presents a risk of infection, with a potentially addictive effect due to previous and concomitant exposure to immunomodulatory treatments. In particular, the risk of serious viral infections has been documented, whether primary infections or reactivations (e.g., EBV, VZV, etc.) or exacerbations (e.g., of the JC virus). Three cases of progressive multifocal leucoencephalopathy (PML) have been reported. This infection has been observed in lupus and vasculitis, where treatment also includes other immunosuppressants. To date, the causal relationship between rituximab (Mabthera®) and progressive multifocal leucoencephalopathy has not been established. However, the appearance of symptoms in the central nervous system must indicate a diagnosis of PML which must be substantiated by an MRI of the brain ± a lumbar puncture with screening for the JC virus by PCR.

## 6.3. Treatment regimen

### Rituximab dose:

The dose recommended by the Roche laboratory is the one used for treating rheumatoid arthritis (2): i.e., 2 injections of 1000 mg of Rituximab at an interval of 2 weeks.

A maintenance treatment in reorder to minimise the risk of a relapse (as for lymphoma (26)) will be carried out: one additional injection of 1000 mg of Rituximab in the 6^th^ month after the previous injection (M6.5 (+/-8 days)).

### Premedication:

Corticosteroids: all patients will be given 100 mg of Methylprednisolone via slow IV, 30 minutes before the Rituximab injection in order to reduce the potential side effects.

Antipyretics: it is recommended that each patient receive premedication of 1 g (iv or by mouth) of Paracetamol, 30 to 60 minutes prior to the Rituximab infusion in order to reduce the potential side effects.

Antihistamines: it is recommended that each patient receive premedication with an H1 antihistamine (by mouth, e.g. Cetirizine, ZYRTEC: One 10 mg capsule), 30 to 60 minutes before the Rituximab infusion in order to reduce the potential side effects.

### Concomitant treatments: depending on the context:

1. plasma exchanges prior to the Rituximab injection may be authorised.
2. Concomitant corticosteroid treatment is authorised, up to one dose of 1 mg/kilo/day.
3. Bolus doses of Methylprednisolone are authorised.
4. Maintaining a treatment with immunosuppressants (methotrexate, cyclophosphamide, azathioprine, mycophenolate mofetil) or polyvalent immunoglobulins is recommended. However, we will try to limit combined treatments: corticosteroid treatment and one of the immunosuppressants listed above can be maintained (prescribed for longer than 6 months, if possible), to which rituximab will be added, but multiple combinations will be avoided.

## Assessment of Rituximab's toxicity

In the randomised trial during rheumatoid arthritis, few serious side effects were observed (one case of high blood pressure, one case of pneumonia, one case of staphylococcus aureus septicaemia and one case of renal failure) (2).

The tolerance assessment will be carried out according to the usual clinical and biological monitoring criteria:

1. Clinical parameters: the clinical exam before and after the Rituximab injection will include blood pressure, respiratory rate, oxygen saturation (finger) and temperature, every 15 minutes for the first 2 hours then every 2 hours for the next 8 hours and finally 3 times a day for the 2 days following the first injection, and 8 hours for the 2^nd^ injection. These parameters will also include a clinical exam once a day.
2. Biological parameters: the usual biological parameters (CBC, natremia, kalemia, chloremia, creatinemia CPK level, bilirubin, ALAT, ASAT, gamma GT, LDH, CRP) will be carried out before each Rituximab injection.
3. Immunological parameters: immuno-phenotyping including markers such as: CD19, CD20, CD4, CD25, CD45 RO, CD69, CTLA-4, CD40L, CD8 will be carried out at the same time.
4. Autoantibody titration will be carried out at the same time.

## Rules for stopping Rituximab (temporarily or permanently)

In the case of fever, chills, a drop in blood pressure or in saturation or breathing difficulties, the infusion will be stopped immediately and will not be resumed until medical approval has been given. The doctor shall determine the degree of severity.

In the case of a serious event (as defined below) which could be severe hypotension, severe dyspnoea with bronchospasm serious viral infection (primary infection, exacerbation (for example as EBV, VZV, etc.) or reactivation (PML for example), rituximab must be stopped permanently. The patient will remain in the study and will continue to be monitored as scheduled (see § 4.4 Rules for definitively or temporarily stopping the study).

In the case of mild to moderate reactions as listed below:

| **Flow rate** | **Fever** | **Chills** | **Congestion of the mucosa Oedema** | **Drop in systolic BP** |
| --- | --- | --- | --- | --- |
| **Reduce the flow rate by 50% if**  **one of these symptoms develops** | > 38.5°C | moderate | Moderate | > 30 mm Hg |

When the symptoms presented disappear, the infusion can resume but at a flow rate 50% lower than that which was in place before the symptoms appeared. **Monitoring after the incident must be carried out every 15 minutes for the first hour then every hour.**

# 7 Statistics

## 7.1. Statistical analysis plan and justification for the number of patients to include:

**Myositides**

Twelve patients with inflammatory myopathy associated with anti-synthetases (JO-1, PL-7 or PL-12) and 12 patients with inflammatory myopathy associated with anti-SRP will be included in the study.

For each group of patients (anti-synthetase and anti-SRP), if efficacy is observed in at least 6 patients, it will be possible to conclude that the response rate is above 25% (limit lower than 90% of the confidence interval for an observed response rate of 50%).

For a given subject, efficacy is determined by an improvement in muscle strength, defined by at least a 2-point gain on the Kendall scale in at least 2 different muscle groups (i.e. a gain of at least 4 points) (18) (see Appendix 1), in the 12th month.

**Myasthenias**

Twelve patients with generalised and refractory myasthenia with anti-RAch antibodies will be included in the study.

If success is observed in at least 6 patients, it will be possible to conclude that the response rate is above 25% (limit lower than 90% of the confidence interval for an observed response rate of 50%).

For a given subject, efficacy is determined by an improvement in muscle strength defined by at least a 20-point gain in the MMG score (47, 48) in the 12^th^ month.

## 7.2. Statistical analyses

Statistical analysis will be carried out after all the data has been verified and will include:

- - description of the patient population
  - description of the efficacy criteria (clinical and biological)
  - description of the tolerance criteria (clinical and biological)
  - description of the immune response

The primary and secondary criteria for evolution in the muscle testing scores, evolution in the quality of life, evolution in the CPKs and in the AAB titer, etc., will be assessed by the individual variation in the measurement compared to the measurement taken at D0.

Statistical tests will be carried out on the individual data using the percentages for qualitative variables and averages (interquartile range) for the quantitative variables.

The statistical analyses will be carried out within the Inserm U 720 unit under the responsibility of Dr D Costagliola.

# 8. Regulatory aspects:

## 8.1. Anticipated methods and timetable for measuring, collecting and analysing the parameters for assessing safety

## 8.1.1.Steering Committee

The committee will be made up of the clinicians who initiated the project, the biostatistician responsible for the project, representatives of the sponsor and the URC named for this study.

The committee will determine the general organisation and procedure for the study and will coordinate the information.

This includes initial determination of the methodology and during the course of the study, determination of the approach to be adopted in unexpected situations. The committee will also monitor the conduct of the study, in particular relating to tolerance and adverse events.

## 8.1.2. Independent Supervisory Committee

A supervisory committee that is independent of the study will be put in place.

Its composition will be determined at the start of the study. It will be made up of individuals with no connection to the study including at least one clinician specialising in the pathology being studied and a pharmacologist/pharmacovigilance specialist.

This committee consultative and decision-making role in advising the sponsor on medical issues such as tolerance and adverse events.

This supervisory committee will meet in order to assess the side effects seen in the first 4 patients to receive a combination of azathioprine and rituximab after 6 months of treatment, as well as after the first 4 patients to receive a combination of mycophenolate mofetil and rituximab.

The study can be interrupted at any time by decision of the supervisory committee, in particular if serious adverse reactions occur more frequently than expected (see below) after the rituximab injections.

## 8.2. Description of parameters for assessing safety

#### Adverse event

Any untoward medical occurrence in a biomedical research subject, which does not necessarily have a causal relationship with the study or with the product being studied.

#### Adverse reaction to an experimental medication

Any untoward and adverse reaction to an experimental medication regardless of the dose administered

#### Serious adverse event or reaction

Any adverse event or reaction that results in death, threatens the life of the research subject, requires hospitalisation or prolongs hospitalisation, causes a severe or long-term disability or handicap, or results in a congenital abnormality or deformity, and in the case of a medication, regardless of the dose administered.

#### Unexpected adverse reaction to an experimental medication

Any adverse reaction of which the nature, severity or evolution are not consistent with the information provided in the summary of the product characteristics if the medication is authorised, and in the investigator's brochure if the medication is not authorised.

#### New fact

Any new information regarding safety, that could lead to a reassessment of the benefit-risk ratio for the study or experimental medication, or which could be sufficient to consider modifications to the administration of the experimental medication, during the study.

## 8.3. Procedures in place for recording and reporting adverse events

## 8.3.1 Non-serious adverse events

Any adverse event - non serious according to the definition above - observed during the study and during follow-up must be reported in the relevant section of the case report form.

Only one event must be reported per item. The event can be a symptom, a diagnosis or the result of an additional test considered to be significant. All the clinical or para-clinical elements that make it possible to describe the events as accurately possible must be reported.

## 8.3.2 Serious adverse events (SAE):

The investigators must report serious adverse events as defined above to the AP-HP as sponsor **immediately**.

**The investigator completes the serious adverse events forms (in the case report form for the study) and sends them to the DRCD by fax at 01 44 84 17 99 within 48 hours** (if possible, after making a telephone call immediately to 01 44 84 17 23 in the case of death or in a life-threatening situation).

The investigator must also inform the URC responsible for the study that the SAE has occurred.

For each serious adverse event, **the investigator must issue an opinion on the causal relationship between the event and each experimental medication and any other treatments.**

Obtaining information about the description and assessment of an adverse event might be impossible in the time given for the initial declaration.

**Therefore, the clinical evolution and the results of any clinical reports and diagnostic and/or laboratory exams, or any other information making it possible to adequately analyse the casual relationship, will be reported:**

- **either on the initial declaration of the SAE if this is available immediately**
- **or subsequently and as soon as possible, by faxing a new completed SAE declaration (and specifying that this is a follow-up to a reported SAE, including the follow-up number).**

All the declarations made by the investigators must identify each subject participating in the study **with a unique code number** assigned to each participant.

**If the death of a participant is reported**, **the investigator will provide the sponsor and the ethics committee with all additional information requested** (hospitalisation report, autopsy report, etc.).

Any new facts that occur during the study or in the context of the study, originating from data in the literature or from ongoing studies, must be reported to the sponsor.

#### *- Declaring serious adverse events to the Health Authorities*

This is carried out by the Pharmacovigilance Division of the DRCD, after an assessment of the severity of the adverse event, the causal relationship with each experimental medication and any other treatments and the unexpected nature of the adverse events.

The promoter must report all suspected unexpected serious adverse reactions to the competent authorities within the legal time frame.

**The promoter will report any safety data or new fact that could significantly alter the assessment of the benefit-risk ratio for the experimental medication, or for the study, or which could lead to the possibility of altering the administration of the experimental medication or altering the conduct of the study, to the competent authorities, to the Comité de Protection des Personnes (ethical review board) and to the study investigators.** For example:

a) any clinically significant increase in the frequency with which an expected serious adverse reaction appears

b) suspected unexpected serious adverse reactions occurring in patients who have finished the trial and about whom the sponsor is notified by the investigator, who also provides any follow-up reports

c) any new fact relating to the conduct of the clinical trial or the development of the medication, if the new fact is likely to affect participant safety Examples:

- a serious adverse event likely to be related to the investigations and to the trial's diagnostic procedures and which could modify the conduct of this trial
- a significant risk for the trial participants such as ineffectiveness of the medication used in the trial in treating a life-threatening illness
- significant safety results from a recently completed study carried out on animals (such as a carcinogenicity study)
- the premature termination, or temporary interruption, of a trial conducted with the same medication in another country, for safety reasons
- an unexpected serious adverse reaction associated with a non-experimental medication required for carrying out the trial, (e.g.: challenge agents, rescue treatment)

d) recommendations from the independent supervisory committee, if applicable, if they are relevant to the safety of the participants

e) any unexpected serious adverse reaction reported to the sponsor by another sponsor of a trial carried out in a different country but relating to the same medication

## 8.4. Procedures for and duration of participant monitoring after adverse events have occurred

All patients presenting an adverse event must be monitored until the event is resolved or stabilises.

- If the event is not serious, its evolution will be reported on the corresponding page of the relevant section on the case report form
- If the event is serious, SAE follow-up will be sent to the DRCD.

# 9 RIGHT TO ACCESS SOURCE DATA AND DOCUMENTS

People with direct access in accordance with the legislative and regulatory provisions in force, and particularly Articles L.1121-3 and R.5121-13 of the Public Health Code (e.g. investigators, quality control officers, monitors, clinical research assistants, auditors and anyone invited to collaborate in the study) will take all necessary precautions to preserve the confidentiality of information about the experimental medications, the trials, the trial participants and in particular the identity of the participants and the results obtained. The data collected by these people during quality inspections and audits will therefore be kept anonymous.

# 10 QUALITY CONTROL AND ASSURANCE

**Research will be supervised according to the sponsor’s standard procedures.**

The conduct of the study in the investigating centres and management of the subjects will be undertaken in accordance with the Declaration of Helsinki and current Good Clinical Practice.

## 10.1 Monitoring procedures

This study is classified as D risk according to the promoter's procedures.

The CRAs representing the sponsor will visit the investigating centres at a rate corresponding to the patient follow-up plan in the protocol, inclusion in the various centres and the risk level attributed to the research.

- Opening visit of each centre: before inclusion, to set up the protocol and make contact with the various biomedical research agents.

- During subsequent visits, the case report forms will be reviewed by the CRAs as research progresses. Each centre’s principal investigator and the other investigators who will include or monitor the subjects undertake to welcome the CRAs at regular intervals.

During these onsite visits and in keeping with Good Clinical Practice, the following points will be reviewed:

- Adherence to the defined research protocol and procedures
- Verification of the patients' informed consent forms
- Examination of source documents and comparison with the data in the case report forms regarding accuracy, missing data and data consistency according to the rules set out in the procedures of the DRCD

- Closing visit: collection of the case report forms, pharmacy report, biomedical research documents, archiving

## 10.2 Transcription of the data in the case report form

All information required under the protocol will need to be entered onto the case report form and the investigator will need to provide an explanation for all missing data.

All clinical and para-clinical data will need to be transferred to the case report forms as they are obtained. Data will need to be copied onto these forms clearly and legibly, using black ink (to facilitate duplication and computer entry).

If incorrect data are found on a case report form, they will be clearly crossed off and new data will be copied onto the form with the date and the initials of the investigating team’s member who makes the correction.

Anonymity will be preserved by using each subject’s code number and initials on all required research documents and by deleting personal data from copies of source documents intended for research documentation using the appropriate means.

Data on computer files will be declared to the French data protection authority using the appropriate procedure.

# 11. LEGAL AND ETHICAL CONSIDERATIONS

The sponsor is defined by French Act 2004-806 of 9 August 2004. In this study, AP-HP is the sponsor and the Département de la Recherche Clinique et du Développement (Clinical Research and Development Department, DRCD) performs regulatory missions.

*Before starting research, each investigator will give the sponsor’s representative a copy of his/her* ***personal curriculum vitæ, dated and signed,*** *with his/her number of registration in the Order of Physicians.*

## 11.1 Request for authorisation from AFSSAPS

To be able to start the study, AP-HP, as sponsor, has to submit a request for authorisation to the competent authority, AFSSAPS. The competent authority, defined in Article L. 1123-12, will give its opinion as to the safety of the study participants, considering particularly the safety and quality of the products used during the study in accordance, where applicable, with the guidelines in force, their conditions of use and personal safety with regard to the acts performed and the methods and procedures used for participant follow-up.

## 11.2 Request for an Opinion from the Comité de Protection des Personnes (ethical review board)

In accordance with Article L.1123-6 of the Public Health Code, the sponsor has to submit the research protocol to an ethical review board. The board’s opinion is notified by the sponsor to the competent authority before the start of research.

## 11.3 Modifications

The coordinating investigator must inform the DRCD of any plans to modify the protocol.

Modifications must be characterised as substantial or not.

A substantial modification is a modification that is likely, in one way or another, to modify the guarantees provided for the biomedical research subjects (modification of an inclusion criterion, extension of the inclusion period, participation of new centres, etc.).

After the study begins, any sustainable modifications to the study on the sponsor’s initiative have to obtain, prior to their implementation, a favourable opinion from the board and an authorisation from the competent authority. In this case, if necessary, the board ensures that the research subjects have filled out a new consent form.

Furthermore, any extension of research (significant modification of the treatment regimen or the included populations, extension of treatment and/or therapeutic procedures not initially included in the protocol) will be considered new research.

When making any substantial modification, after paying a fee the sponsor must submit a request for authorisation to AFSSAPS and/or a request for an opinion from the ethical review board.

## 11.4 CNIL declaration

The law specifies that the electronic file containing personal data collected for the study must be declared before the effective start of the study.

A reference methodology specific to the processing of personal data carried out as part of biomedical studies defined by French Act 2004-806 of 9 August 2004, because they fall within the scope pf Articles L.1121-1 et seq of the French Public Health Code, was established by CNIL in January 2006.

This methodology simplifies the declaration procedure when the nature of the data collected in the study is compatible with the list provided by CNIL in its reference document.

When the protocol is subject to data quality control carried out by a CRA representing the sponsor and if the protocol falls within the scope of application of the simplified CNIL procedure, the DRCD, as sponsor, will ask the manager of the electronic file to commit in writing to compliance with the MR06001 simplified reference methodology.

## 11.5 Information sheet and Informed consent

Written consent must be collected from all study participants before any procedure required by biomedical research.

*The protocol will be explained and handed to patients who are hospitalised or seen during a consultation. They will also be given the information sheet.*

*After a reflection period, the patient can potentially be included, as soon as the consent form is signed.*

## 11.6 Final study report

The final research report will be written jointly by the study's coordinator and biostatistician. This report will be submitted to each investigator for his/her opinion. Once consensus has been reached, the final version will be signed by each investigator for endorsement and sent to the sponsor as soon as possible after the effective end of research. A report written according to the competent authority’s reference plan will need to be sent to the competent authority and ethical review board within one year after the end of research, which should be understood as the last follow-up visit of the last included subject. This period is shortened to 90 days if research is prematurely stopped.

# 12. DATA PROCESSING AND STORAGE OF RESEARCH DOCUMENTS AND DATA

Documents related to research in the scope of the legislation on biomedical research must be archived by all parties for a 15-year period after the end of research.

(see GCPs, section 8: essential documents)

this indexed archival includes:

- Copies of the AFSSAPS' letter of authorisation and the ethical review board’s mandatory opinion
- Successive versions of the protocol (identified by the version no. and date)
- Letters of correspondence with the sponsor
- The subjects’ signed consent forms in sealed envelopes (signed by those responsible for exercising parental authority in the case of participants who are minors) with the corresponding list or register of inclusion
- Each included subject’s completed and validated case report form
- All specific study annexes
- The final report for the study after statistical analysis and quality control (copy sent to the sponsor)
- Certificates from any audits undertaken during the study

The database used for statistical analysis must also be archived by the analysis manager (paper or electronic format)

# 13. INSURANCE AND SCIENTIFIC COMMITMENT

## 13.1 Insurance

L'Assistance Publique-Hôpitaux de Paris is this study’s sponsor. In accordance with the legislation on biomedical research, it has taken out insurance from the company GERLING KonZern for the full study period, covering its own civil liability and that of any agent (doctor or research staff) (Act no. 2004-806, Art L.1121-10 of the Public Health Code).

L'Assistance Publique-Hôpitaux de Paris reserves the right to interrupt the study at any time for medical or administrative reasons. In this case a notification will be given to the investigator.

## 13.2. Scientific commitment

Each investigator will undertake to comply with the legislation and to carry out the research according to G.C.P, adhering to the Declaration of Helsinki terms in force. To that end, a copy of the **scientific commitment (DRCD-type document),** dated and signed **by each investigator** in each participating centre’s clinical department, will be given to the sponsor’s representative.

# 14 PUBLICATION RULES

**AP-HP is the owner of the data, which cannot be used or disclosed to a third party without its prior approval**.

The first signatories of publications will be the individuals who have actually participated in the protocol's development and implementation and in the preparation of its results.

*As a precaution, a drafting committee must be formed and the order of the signatories can be established in advance.*

L’Assistance Publique-Hôpitaux de Paris must be mentioned as the biomedical research sponsor and as financer where applicable. The terms “Assistance Publique - Hôpitaux de Paris” must appear in the authors’ addresses.

# 15. REfErences

1. Boye J, Elter T and Engert A. An overview of the current clinical use of the anti-CD20 monoclonal antibody rituximab. Ann Oncol 2003;14:520-35

2. Edwards JC, Szczepanski L, Szechinski J, et al. Efficacy of B-cell-targeted therapy with rituximab in patients with rheumatoid arthritis. N Engl J Med 2004;350:2572-81

3. Stasi R, Pagano A, Stipa E and Amadori S. Rituximab chimeric anti-CD20 monoclonal antibody treatment for adults with chronic idiopathic thrombocytopenic purpura. Blood 2001;98:952-7

4. Gottenberg JE, Guillevin L, Lambotte O, et al. Tolerance and short term efficacy of rituximab in 43 patients with systemic autoimmune diseases. Ann Rheum Dis 2005;64:913-20

5. Keogh KA, Wylam ME, Stone JH and Specks U. Induction of remission by B lymphocyte depletion in eleven patients with refractory antineutrophil cytoplasmic antibody-associated vasculitis. Arthritis Rheum 2005;52:262-8

6. Levine TD. Rituximab in the treatment of dermatomyositis: an open-label pilot study. Arthritis Rheum 2005;52:601-7

7. Bohan A, Peter JB, Bowman RL and Pearson CM. A computer-assisted analysis of 153 patients with polymyositis and dermatomyositis. Medicine (Baltimore) 1977;56:255-86

8. Vincent A, Palace J and Hilton-Jones D. Myasthenia gravis. Lancet 2001;357:2122-8

9. Dalakas MC, Hohlfeld R. Polymyositis and dermatomyositis. Lancet 2003;362:971-82

10. Dalakas MC. The future prospects in the classification, diagnosis and therapies of inflammatory myopathies: a view to the future from the "bench-to-bedside". J Neurol 2004;251:651-7

11. Gajdos P, Chevret S and Toyka K. Intravenous immunoglobulin for myasthenia gravis. Cochrane Database Syst Rev 2003:CD002277

12. Cherin P, Pelletier S, Teixeira A, et al. Results and long-term followup of intravenous immunoglobulin infusions in chronic, refractory polymyositis: an open study with thirty-five adult patients. Arthritis Rheum 2002;46:467-74

13. Romero NB, Braun S, Benveniste O, et al. Phase I study of dystrophin plasmid-based gene therapy in Duchenne/Becker muscular dystrophy. Hum Gene Ther 2004;15:1065-76

14. Klatzmann D, Cherin P, Bensimon G, et al. A phase I/II dose-escalation study of herpes simplex virus type 1 thymidine kinase "suicide" gene therapy for metastatic melanoma. Study Group on Gene Therapy of Metastatic Melanoma. Hum Gene Ther 1998;9:2585-94

15. Benveniste O, Cherin P, Maisonobe T, et al. Severe perturbations of the blood T cell repertoire in polymyositis, but not dermatomyositis patients. J Immunol 2001;167:3521-9

16. Benveniste O, Herson S, Salomon B, et al. Long-term persistence of clonally expanded T cells in patients with polymyositis. Ann Neurol 2004;56:867-72

17. Benveniste O, Farrugia M, Clover L and Vincent A. MuSK antibody positive myasthenia gravis serum modifies NCAM and MURF-1 expression in C2C12 cultures and mouse muscle in vivo. J NeuroImmunol 2005:in press

18. Hoogendijk JE, Amato AA, Lecky BR, et al. 119th ENMC international workshop: trial design in adult idiopathic inflammatory myopathies, with the exception of inclusion body myositis, 10-12 October 2003, Naarden, The Netherlands. Neuromuscul Disord 2004;14:337-45

19. Mathews MB, Bernstein RM. Myositis autoantibody inhibits histidyl-tRNA synthetase: a model for autoimmunity. Nature 1983;304:177-9

20. Bernstein RM, Morgan SH, Chapman J, et al. Anti-Jo-1 antibody: a marker for myositis with interstitial lung disease. Br Med J (Clin Res Ed) 1984;289:151-2

21. Miller T, Al-Lozi MT, Lopate G and Pestronk A. Myopathy with antibodies to the signal recognition particle: clinical and pathological features. J Neurol Neurosurg Psychiatry 2002;73:420-8

22. Troyanov Y, Targoff IN, Tremblay JL, Goulet JR, Raymond Y and Senecal JL. Novel classification of idiopathic inflammatory myopathies based on overlap syndrome features and autoantibodies: analysis of 100 French Canadian patients. Medicine (Baltimore) 2005;84:231-49

23. Mozaffar T, Pestronk A. Myopathy with anti-Jo-1 antibodies: pathology in perimysium and neighbouring muscle fibres. J Neurol Neurosurg Psychiatry 2000;68:472-8

24. Arlet JB, Dimitri D, Pagnoux C, Boyer O, Maisonobe T, Authier FJ, Bloch-Queyrat C, Goulvestre C, Heshmati F, Atassi M, Guillevin L, Herson S, Benveniste O, Mouthon L. Marked efficacy of a therapeutic strategy associating prednisone and plasma exchange followed by rituximab in two patients with refractory myopathy associated with antibodies to the signal recognition particle (SRP). Neuromuscul Disord. 2006 May;16(5):334-6. Epub 2006 Apr 17.

25. Targoff IN, Johnson AE and Miller FW. Antibody to signal recognition particle in polymyositis. Arthritis Rheum 1990;33:1361-70

26. Hainsworth JD, Litchy S, Shaffer DW, Lackey VL, Grimaldi M and Greco FA. Maximizing therapeutic benefit of rituximab: maintenance therapy versus re-treatment at progression in patients with indolent non-Hodgkin's lymphoma--a randomized phase II trial of the Minnie Pearl Cancer Research Network. J Clin Oncol 2005;23:1088-95

27. Osserman KE, Kornfeld P, Cohen E, et al. Studies in myasthenia gravis; review of two hundred eighty-two cases at the Mount Sinai Hospital, New York City. AMA Arch Intern Med 1958;102:72-81

28. Tindall RS, Rollins JA, Phillips JT, Greenlee R, Wells L and Belendiuk G. Preliminary results of a double-blind, randomized, placebo-controlled trial of cyclosporine in myasthenia gravis. N Engl J Med 1987;316:719-24

29. Saperstein DS, Barohn RJ. Management of myasthenia gravis. Seminar in Neurology 2004;24:41-75

30. Palace J, Newsom-Davis J, B L, et al. A randomized, double-blind trial of prednisolone alone or with azathioprine in Myasthenia Gravis. Neurology 1998;50:1778-1783

31. Meriggioli MN, Ciafaloni E, Al-Hayk KA, et al. Mycophenolate mofetil for myasthenia gravis: an analysis of efficacy, safety, and tolerability. Neurology 2003;61:1438-40

32. Tindall RS, Phillips JT, Rollins JA, Wells L and Hall K. A clinical therapeutic trial of cyclosporine in myasthenia gravis. Ann N Y Acad Sci 1993;681:539-51

33. Ciafaloni E, Nikhar NK, Massey JM and Sanders DB. Retrospective analysis of the use of cyclosporine in myasthenia gravis. Neurology 2000;55:448-450

34. Drachman DB, Jones RJ and Brodsky RA. Treatment of refractory myasthenia: "rebooting" with high-dose cyclophosphamide. Ann Neurol 2003;53:29-34

35. Ponseti JM, Azem J, Fort JM, et al. Long-term results of tacrolimus in cyclosporine- and prednisone-dependent myasthenia gravis. Neurology 2005;64:1641-1643

36. Rowin J, Meriggioli MN, Tüzün E, Leurgans S and Christadoss P. Etanercept treatment in corticosteroid-dependent myasthenia gravis. Neurology 2004;63:2390-2392

37. Newsom-Davis J, Pinching AJ, Vincent A and Wilson SG. Function of circulating antibody to acetylcholine receptor in myasthenia gravis: investigation by plasma exchange. Neurology 1978;28:266-72

38. Toyka KV, Drachman DB, Griffin DE, et al. Myasthenia gravis. Study of humoral immune mechanisms by passive transfer to mice. N Engl J Med 1977;296:125-31

39. Engel AG. Myasthenia gravis and myasthenic syndromes. Ann Neurol 1984;16:519-34

40. Patrick J, Lindstrom J. Autoimmune response to acetylcholine receptor. Science 1973;180:871-2

41. Gajra A, Vajpayee N and Grethlein SJ. Response of myasthenia gravis to rituximab in a patient with non-Hodgkin lymphoma. Am J Hematol 2004;77:196-7

42. Wylam ME, Anderson PM, Kuntz NL and Rodriguez V. Successful treatment of refractory myasthenia gravis using rituximab: a pediatric case report. J Pediatr 2003;143:674-7

43. Barohn RJ, McIntire D, Herbelin L, Wolfe GI, Nations S and Bryan WW. Reliability testing of the quantitative myasthenia gravis score. Ann N Y Acad Sci 1998;841:769-72

44. Sharshar T, Chevret S, Mazighi M, et al. Validity and reliability of two muscle strength scores commonly used as endpoints in assessing treatment of myasthenia gravis. J Neurol 2000;247:286-90

45. Jaretzki A, Barohn RJ, Ernstoff RM, et al. Myasthenia gravis. Recommendations for clinical research standards. Neurology 2000;55:16-23

46. Wolfe GI, Herbelin L, Nations SP, Foster B, Bryan WW and Barohn RJ. Myasthenia gravis activities of daily living profile. Neurology 1999;52:1487-9

47. Gajdos P, Chevret S, Clair B, Tranchant C and Chastang C. Clinical trial of plasma exchange and high dose intravenous immunoglobulin in myasthenia gravis. Ann Neurol 1997;48:789-796

48. Gajdos P, Sharshar T and Chevret S. Standards of measurements in myasthenia gravis. Ann N Y Acad Sci 2003;998:445-52
